# A negative feedback loop in the GPCR pathway underlies efficient coding of external stimuli

**DOI:** 10.15252/msb.202110514

**Published:** 2022-09-15

**Authors:** Rotem Ruach, Shai Yellinek, Eyal Itskovits, Noa Deshe, Yifat Eliezer, Eduard Bokman, Alon Zaslaver

**Affiliations:** ^1^ Department of Genetics, Silberman Institute of Life Science, Edmond J. Safra Campus The Hebrew University Jerusalem Israel

**Keywords:** calcineurin/TAX‐6, calcium imaging, GPCR signaling, negative feedback, pulsatile response, Neuroscience, Signal Transduction

## Abstract

Efficient navigation based on chemical cues is an essential feature shared by all animals. These cues may be encountered in complex spatiotemporal patterns and with orders of magnitude varying intensities. Nevertheless, sensory neurons accurately extract the relevant information from such perplexing signals. Here, we show how a single sensory neuron in *Caenorhabditis elegans* animals can cell‐autonomously encode complex stimulus patterns composed of instantaneous sharp changes and of slowly changing continuous gradients. This encoding relies on a simple negative feedback in the G‐protein‐coupled receptor (GPCR) signaling pathway in which TAX‐6/Calcineurin plays a key role in mediating the feedback inhibition. This negative feedback supports several important coding features that underlie an efficient navigation strategy, including exact adaptation and adaptation to the magnitude of the gradient's first derivative. A simple mathematical model explains the fine neural dynamics of both wild‐type and *tax‐6* mutant animals, further highlighting how the calcium‐dependent activity of TAX‐6/Calcineurin dictates GPCR inhibition and response dynamics. As GPCRs are ubiquitously expressed in all sensory neurons, this mechanism may be a general solution for efficient cell‐autonomous coding of external stimuli.

## Graphical abstract

A chemosensory neuron in *Caenorhabditis elegans* shows remarkable computational capacities to efficiently encode external stimuli. A TAX‐6‐mediated negative feedback loop within the GPCR signaling pathway underlies these computational capacities.

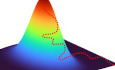

## Introduction

Animals' fitness critically depends on efficient coding of environmental signals. For example, chemical cues allow animals to locate food sources, a mating partner, and avoid possible dangers. In nature, chemical cues often form complex spatiotemporal patterns. To support efficient navigation based on chemical cues (a process known as chemotaxis), animals need to accurately extract the relevant signals and robustly relay this information to subsequent neural layers.

There are two major modes by which animals may experience chemical cues. In rapidly changing environments (e.g., wind carrying plumes or abrupt flow changes), animals will sense instantaneous steep changes in stimulus concentration resembling a step‐like function of the stimulus (Murlis *et al*, 1992). Alternatively, in enclosed and turbulent‐free environments, where diffusion processes dominate, smooth chemical gradients will be formed. Under these conditions, animals will typically experience a gradual change in stimulus concentration. In both cases, stimulus concentrations may vary across several orders of magnitude imposing a great challenge for animals to reliably detect and follow stimulus changes (van As *et al*, 1985; Shirley *et al*, 1987; Gaudry *et al*, 2012; Sourjik & Wingreen, 2012; Levy & Bargmann, 2020).

To efficiently chemotax in complex varying environments, living organisms employ at least two sensory‐coding principles: exact adaptation and logarithmic coding. Exact adaptation implies that the sensory response to a stimulus is transient, and following an initial change, the response resumes to its baseline levels. This way, the sensory system becomes idle to respond upon encountering impending changes in stimulus levels (Berg & Tedesco, 1975; Zufall, 2000; Ferrell, 2016; Tu & Rappel, 2018). Logarithmic coding, also known as the Weber–Fechner law, is a hallmark of sensory systems found across different organisms and sensory modalities (Fechner, 1948; Laughlin, 1989). It dictates that perception depends on the ratio between stimulus change and the background level, thus effectively coding a logarithmic scale of the stimulus. This allows sensory systems to rescale their responses across several orders of magnitude of the signal (Lazova *et al*, 2011). Interestingly, this feature is thought to be intrinsically implemented in the receptor's thermodynamic properties, where increased ligand concentrations induce receptor allosteric modulations (e.g., phosphorylation), which shift the binding affinity between the receptor and its ligand (Olsman & Goentoro, 2016).

In single‐cell organisms, such as *Escherichia coli* bacteria, these principles are implemented by intracellular signaling pathways to support a biased‐random walk chemotaxis strategy (Block *et al*, 1983; Barkai & Leibler, 1997; Tu *et al*, 2008; Shimizu *et al*, 2010; Tu, 2013). In this navigation strategy, cells control the probability for making turns: increasing concentrations of an attractant suppress turning probabilities, such that the cells are more prone to continue moving forward, while decreasing concentrations of the attractant increase turning probabilities (Sourjik & Wingreen, 2012). Crucially, to maintain responsiveness over time, cell activity resumes its basal level even if the stimulus remains constantly on (thus implementing exact adaptation), allowing it to be idle to detect and respond to future changes.

In multicellular organisms, equipped with a neural network, one may assume that similar features may be attributed to dynamics within defined neural circuits. Interestingly, however, individual sensory neurons may also implement such computations in a cell‐autonomous manner (Tanimoto *et al*, 2017; preprint: Desrochers *et al*, 2018). For example, the nervous system of *Caenorhabditis elegans* nematodes consists of 302 neurons (White *et al*, 1986; Cook *et al*, 2019). A single pair of chemosensory neurons, the AWA neurons, exhibits a range of coding features in response to diacetyl, a chemoattractive food cue secreted from bacteria in decomposing fruits (Choi *et al*, 2016). Smooth and slowly increasing gradients of diacetyl lead to a pulsatile activity in the AWA neurons, where the frequency and the amplitude of the pulses increase the greater is the temporal derivative of the stimulus (Itskovits *et al*, 2018). This pulsatile activity was also observed in mutant animals, defective in neurotransmitter and neuropeptide secretion, suggesting that these sensory neurons may implement such intricate computation in a cell‐autonomous manner. Furthermore, following a step‐like increase in diacetyl concentrations, AWA calcium levels quickly rise and then return to near‐baseline levels even when diacetyl levels remain constantly high (exact adaptation). These responses are found for a wide range of diacetyl concentrations spanning seven orders of magnitude (Bargmann *et al*, 1993; Bargmann, 2006; Hart & Chao, 2009; Larsch *et al*, 2013, 2015; Itskovits *et al*, 2018).

Behaviorally, AWA activity facilitates forward locomotion, while a decrease in AWA activity promotes turning events (Larsch *et al*, 2015; Itskovits *et al*, 2018). Thus, a pulsatile activity dictates a run and tumble strategy, similar to the biased‐random walk behavior in *E. coli* (Pierce‐Shimomura *et al*, 1999). However, while cells typically adapt to the absolute levels of the stimulus, AWA pulsatile activity also adapts to the first derivative of the gradient (Itskovits *et al*, 2018). This coding principle was shown to support an efficient navigation strategy that outperforms the classical biased‐random walk strategy (Itskovits *et al*, 2018). An intriguing question then arises: how can a single neuron perform all these computations and efficiently encode complex stimulus patterns?

**Figure 1:**
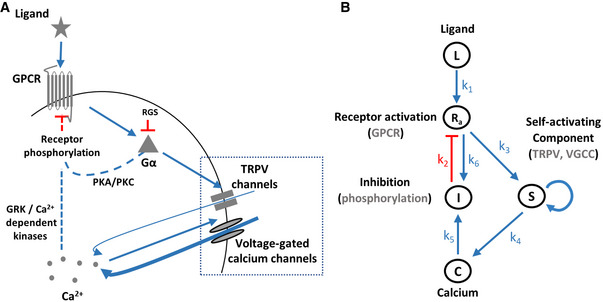
Illustration of the core GPCR signaling pathway and the analogous model A
Key components of the GPCR signaling pathway. Dashed lines mark canonical GPCR pathways, though they have not been verified yet in the AWA neuron.B
An analogous circuit topology that recapitulates the main signaling events related to calcium dynamics. Note that S (TRPV and VGCC) depicts a self‐amplifying module to generate the pulsatile activity. Symbols next to arrows mark the relevant constant in the model's equations. Blue arrows indicate activation, and red lines mark inhibition.

The AWA neuron exclusively expresses the diacetyl G‐protein‐coupled receptor (GPCR), named ODR‐10 (Bargmann *et al*, 1993; Sengupta *et al*, 1996). ODR‐10 activation follows the canonical GPCR signaling pathway: Upon binding of diacetyl, ODR‐10 leads to the dissociation of the trimeric G protein, where the Gα subunit stimulates opening of TRPV channels (Fig 1A), possibly via polyunsaturated fatty acids (Kahn‐Kirby *et al*, 2004; Larsch *et al*, 2015). Calcium ions then flow into the cell leading to a partial depolarization that subsequently triggers a much larger influx of calcium through the voltage‐gated calcium channels (EGL‐19), which culminates in a train of spiking events (Larsch *et al*, 2015; Liu *et al*, 2018).

However, the mechanism by which AWA adapts to the odorant signal is unknown, though evidence suggests that it relies on a negative feedback loop (Rahi *et al*, 2017). A canonical model for GPCR adaptation relies on receptor phosphorylation events via two possible pathways: (i) second messenger‐induced kinases, namely PKA and PKC, which phosphorylate both active and inactive receptors, thus mediating a nonspecific (non‐homologous) adaptation (Fig 1A and B); (ii) G‐protein‐coupled receptor kinases (GRKs) that phosphorylate the active receptors, leading to receptor‐specific (homologous) adaptation. GRKs can be activated by calmodulin, a calcium‐binding protein, suggesting that calcium levels may indirectly regulate receptor adaptation (Lefkowitz, 1998; Zufall, 2000). In addition, regulators of G protein signaling (RGS), which inactivate the Gα subunit via GTP hydrolysis, may also contribute to sensory adaptation (Vries *et al*, 2000; Fukuto *et al*, 2004).

In this study, we demonstrate how a simple negative feedback in the GPCR signaling pathway underlies efficient coding of various spatiotemporal patterns of the stimulus. Remarkably, it enables cell‐autonomous translation of smooth gradients into a series of pulses that adapt to the magnitude of the gradient's first derivative. Furthermore, we identified TAX‐6/Calcineurin as a key component required for the negative feedback that leads to receptor adaptation. A simple mathematical model depicts an array of neural responses of both wild‐type (wt) and *tax‐6* mutant animals. Given the ubiquitous expression of GPCRs in sensory neurons, this mechanism may account for efficient coding in other animals across different sensory modalities.

## Results

### A simple feedback model recapitulates experimental observations for coding complex stimulus patterns

**Figure 2:**
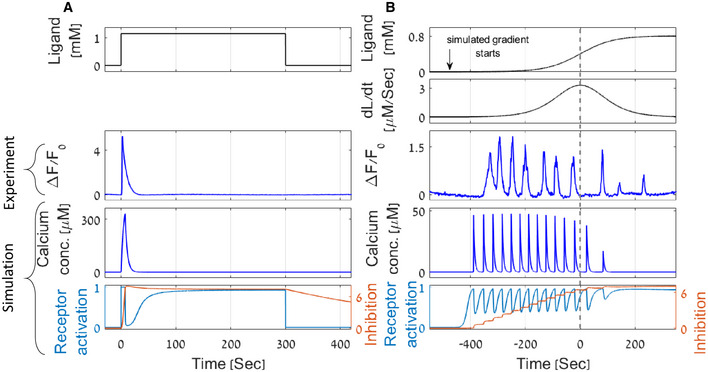
A parsimonious mathematical model recapitulates experimental results demonstrating exact adaptation, pulsatile coding, and adaptation to the magnitude of the first derivative A, B
Experimental results and model simulations of calcium levels in response to a step function (A) and to a smooth sigmoidal function (B) of the stimulus. (A) Following an on step, and during stimulus presentation, calcium levels rise and then return to their basal levels (exact adaptation in experiment and simulation). Receptor activity ($R_{a}$) and inhibition levels (I) reach a new steady state. (B) A sigmoidal gradient of the stimulus elicits a series of calcium pulses that are stronger in the first half of the sigmoid, thus demonstrating adaptation to the gradient's first derivative (dashed line marks the sigmoid midpoint, where the first derivative is maximal). This feature is observed in both experiments and simulations. The stair‐shape inhibition constitutes a discrete memory of previous input levels. Top two panels show the gradient and its derivative. Middle panels depict a representative result of AWA calcium imaging. The two bottom panels present calcium concentrations (C), receptor activation ($R_{a}$), and inhibition (I) levels as simulated by the model for the same gradient. Calcium dynamics was measured using a strain expressing GCaMP in the AWA neuron and is denoted as the GCaMP fluorescence fold change from the initial state $\left(F-F_{0}\right)/F_{0}$. Diacetyl (stimulus) was presented to the worm using a custom‐made microfluidic device (see Materials and Methods). Source data are available online for this figure.

Studies in *C. elegans* worms revealed that a single sensory neuron type, AWA, cell‐autonomously implements key features required for efficient chemotaxis: (i) It codes the ligand concentration in a logarithmic‐like scale, so that neural responses remain similar across orders of magnitude of ligand concentration (Larsch *et al*, 2015; Itskovits *et al*, 2018). (ii) It responds with a single pulse to a step function and with multiple pulses during a continuous gradual increase in the stimulus concentration (Fig 2A; Larsch *et al*, 2013, 2015; Itskovits *et al*, 2018). (iii) It shows an exact adaptation following a step function of the stimulus (Fig 2A; Larsch *et al*, 2015; Itskovits *et al*, 2018). (iv) The mean frequency and the amplitude of the pulses, induced by smooth increasing stimuli, correlate with the first derivative of the gradient and also adapt to it (Fig 2B; Itskovits *et al*, 2018). Thus, the steeper the change in the gradient (higher first derivative), the higher are the frequency and the amplitude of the neural activity. On longer timescales, this activity adapts to the magnitude of the stimulus first derivative, such that the frequency and the amplitude of the pulses decrease if not facing increasing first derivative changes in the stimulus.

To understand how these intricate coding schemes can be cell‐autonomously implemented, we considered the GPCR signaling pathway and constructed a simple parsimonious model of its known signaling components (Fig 1B). In this model, the stimulus ligand (L) binds the GPCR to convert it to its active state ($R_{a}$). The active state, through the small Gα subunit, activates TRPV and subsequently VGCC (together denoted as S) to depolarize the cell by increasing cytoplasmic calcium levels (C). Elevated calcium levels activate an array of proteins, including GRKs (denoted as I), which lead to phosphorylation and eventual inhibition of the GPCRs. Gα‐mediated activation of PKA and PKC also contributes to GPCR inhibition, and this inhibition is also included in the inhibitory component (I).

The dynamics of this signaling pathway can be simulated using a set of four equations (equations (1), (2), (3), (4)), where each equation describes the temporal change of one of the main signaling components (see Appendix Note S1 part 1 for extended descriptions).

(1) $$R_{a}={\left(1+\exp\left(-k_{1}\log\left(\frac{L}{L_{0}}\right)+k_{2}I\right)\right)}^{-1},\;R_{a}\;\epsilon \;\left(0,1\right)$$

(2) $$\frac{dS}{\mathrm{dt}}=k_{3}\left(R_{a}-R_{t}\right)S,\;S\;\epsilon \;\left(0,1\right)$$

(3) $$\frac{\mathrm{dC}}{\mathrm{dt}}=k_{4}S-\frac{1}{\tau_{c}}\left(C-C_{0}\right)$$

(4) $$\frac{\mathrm{dI}}{\mathrm{dt}}=k_{5}\left(C-C_{0}\right)R_{a}+k_{6}R_{a}-\frac{1}{\tau_{I}}\left(1-R_{a}\right)I$$

The fraction of active receptors ($R_{a}$, equation (1)) is described by a sigmoid function, where the levels of active receptors scale logarithmically with the concentration of the ligand (as was shown for the dynamics of *E. coli* receptors; Tu *et al*, 2008). $R_{a}$ inhibition (e.g., via phosphorylation) is linearly dependent on the levels of the inhibitors I. This representation is motivated by previous reports showing that when the sensor's activity increases logarithmically with ligands' concentration, the negative feedback loop enables logarithmic coding (Tu *et al*, 2008).

Equation 2 describes a switch‐like transition in the state of TRPV and VGCC (S). These channels open once active receptors cross a threshold value, $R_{t}$, and close otherwise. The switch‐like dynamics is due to self‐activating voltage‐dependent properties of the VGCCs, through which the majority of the calcium enters the cell. This self‐amplification underlies the pulsatile activity and is a known molecular switch motif (Ferrell & Machleder, 1998; Zhang *et al*, 2007).

Intracellular calcium concentration (C) increases upon opening of the channels (S) while a first‐order removal term decreases it back to baseline levels, $C_{0}$ (equation 3). The fact that calcium removal is proportional to calcium concentration enables exponential decay in calcium levels, as was also experimentally observed (Itskovits *et al*, 2018). Finally, equation 4 describes the circuit negative feedback (I), where calcium‐dependent (e.g., GRKs, denotes by the $k_{5}$ arrow) and calcium‐independent (e.g., PKA/PKC, denoted by the $k_{6}$ arrow) pathways enhance inhibition, and a first‐order removal term suppresses it. Borrowing the exact adaptation concepts developed for the *E. coli* chemosensory circuit, we posit that inhibition is proportional to the fraction of active receptors ($R_{a}$), and the removal term is proportional to the fraction of inactive receptors ($1-R_{a}$). A detailed description and analysis of the mathematical model are available in Appendix Note S1 parts 1–3 and Appendix Table S1.

Remarkably, the simple parsimonious model of a negative feedback in the GPCR signaling pathway captures the key features that we observed experimentally (Fig 2): An on step of the diacetyl stimulus resulted in a single calcium pulse, which decayed to baseline levels even though the stimulus remained constantly present (Fig 2A). Similarly, both the experiments and the simulation results showed pulsatile dynamics in response to smooth sigmoid gradients of the stimulus (Fig 2B). Moreover, the amplitude and the frequency of the pulses increase with the gradient's first derivative but also adapt to it. Thus, in response to a sigmoidal gradient, in which the first derivative is symmetric around the gradient's midpoint, the pulsatile response is stronger in the first half, up to the maximal first derivative point, and decreases thereafter (Fig 2B). Of note, the pulsatile activity greatly varies between individuals (Appendix Fig S3A). This variability, which can be reproduced when simulating the model with slightly different random variables of the parameter space (Appendix Fig S2D–F), manifests in the number of the pulses, their amplitude, as well as in their frequency. Nevertheless, the pulsatile response and the correlation of the pulse's amplitude and frequency with the gradient's first derivative are robust features.

How does the circuit model translate an increasing gradient of the stimulus input into a pulsatile calcium activity, an output that both increases with the input's derivative and adapts to it? Consider a smooth slowly increasing stimulus: At the beginning, calcium levels are close to baseline levels such that the inhibition is relatively constant (equation 4). As stimulus concentration gradually increases, the fraction of active receptors also rises (equation 1). Once it crosses a threshold value, $R_{t}$, TRPV, followed by the VGCCs, will open (equation 2), resulting in a calcium pulse (equation 3). The high intracellular calcium concentration enhances a quick inhibition (equation 4), thus reducing the fraction of active receptors below the critical threshold, $R_{t}$ (equation 2), in which case, the pulse is terminated by exponential removal of the calcium (equation 3). Relaying the signaling output, C, to directly facilitate inhibition is known as an integral feedback and underlies exact adaptation (Yi *et al*, 2000). As time passes and the stimulus concentration increases, this process will repeat itself to produce an additional calcium pulse. Since receptors' activation is logarithmically dependent on the input, the change required in the input levels to elicit a consecutive pulse becomes increasingly larger. For a linear gradient, this implies that the time interval between consecutive pulses will increase, effectively leading to an adaptation to the input gradient. In fact, a detailed mathematical analysis shows that the time interval between consecutive pulses increases exponentially with the pulse index number and is inversely proportional to the linear gradient derivative (Appendix Note S1 part 3).

Our model qualitatively captures additional functional features observed in AWA response dynamics (Appendix Fig S1). In response to repetitive high‐frequency steps of the stimulus, inhibition removal may be too slow, and hence, activity would not be observed in response to all repetitive stimulations, a phenomenon known as periodic skipping (compare Appendix Fig S1B and C and see also experimental evidence in Larsch *et al*, 2015; Rahi *et al*, 2017). Furthermore, in response to short on steps, our model predicts gradual adaptation: a decrease in response amplitude between consecutive steps (Appendix Fig S1A), which was also observed experimentally (Larsch *et al*, 2015; Rahi *et al*, 2017).

Notably, the main features of the model outputs are robust to changes in the values of the different parameters. We simulated the system behavior while varying each parameter by ∼100‐fold (See Materials and Methods). Despite the broad parameter space, the qualitative dynamics remained largely unaffected, where key features such as exact adaptation and adaptation of the pulsatile activity were maintained (Appendix Fig S2, Appendix Table S1). When simultaneously varying all the model's parameters (except for $R_{t}$) by 10‐fold, the qualitative dynamics remained intact in 75% of the cases (see Materials and Methods). Moreover, the model's output is robust to over 10,000‐fold variation in stimulus concentration, a necessary requirement for a versatile sensory system that can code and robustly respond to a range of concentrations. These results indicate that the model shows robust outputs that are insensitive to the exact values of its variables.

### TAX‐6/calcineurin is required for the pulsatile response

A classic feedback circuit that achieves full adaptation requires the inhibition to depend on the circuit output (Barkai & Leibler, 1997; Tu *et al*, 2008). We therefore analyzed several mutants that had been suggested to affect adaptation in *C. elegans* worms. These included: *odr‐3*, which codes the Gα protein, and *eat‐16*, an RGS homolog regulating Gα activity (Roayaie *et al*, 1998; Hajdu‐Cronin *et al*, 1999); *grk‐2*, which affects G‐protein signaling in sensory neurons (Wood & Ferkey, 2016); *osm‐6*, which is responsible for cilia integrity and intraflagellar transport (Larsch *et al*, 2015); *arr‐1*, an arrestin homolog (Fukuto *et al*, 2004; preprint: Merritt *et al*, 2021); and *tax‐6*, a calcineurin shown to cell‐autonomously mediate adaptation in different sensory neurons (Kuhara *et al*, 2002).

**Figure 3:**
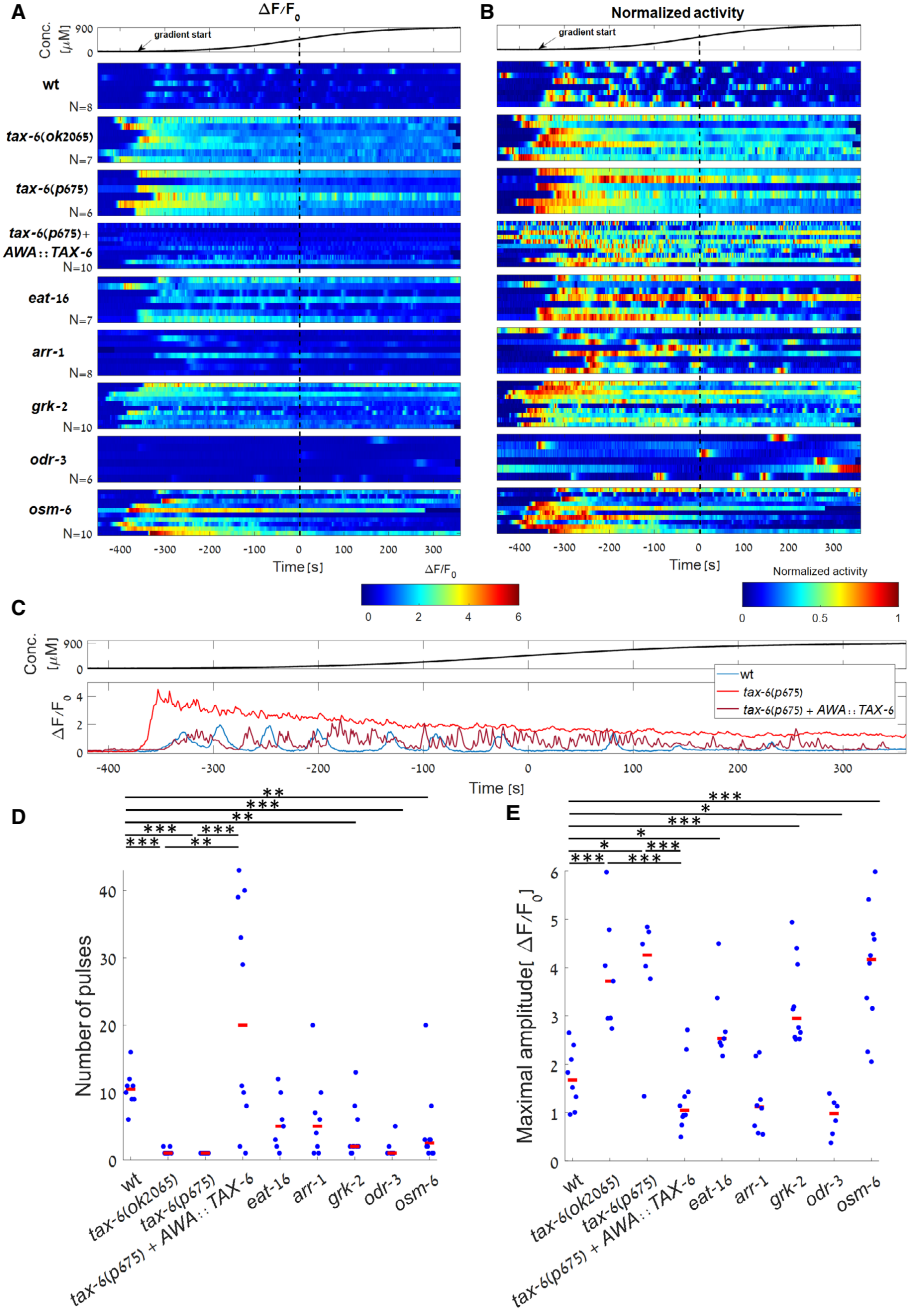
Analysis of dynamic responses and adaptation in different mutant backgrounds A, B
Response profiles of individual wt and mutant worms to a sigmoidal gradient (top). Shown are the fold change activity (A) and the normalized activity (B) of wt, *tax‐6(ok2065)*, *tax‐6(p675)*, *tax‐6(p675)* + *AWA::TAX‐6*, *eat‐16*, *arr‐1*, *grk‐2*, *odr‐3* and *osm‐6* mutant worms (N=8,7,6,10,7,8,10,6,10 worms, respectively). Due to the high variability of the GCaMP baseline fluorescence, $F_{0}$, between and within each of the mutant strains, we considered fluorescence fold change, $\left(F-F_{0}\right)/F_{0}$, when analyzing pulsatile activity. *Tax‐6* mutants lacked any pulsatile activity (raw traces are shown in Appendix Fig S3). Time is aligned to the gradient's inflection point, in which the first derivative is maximal (dotted black line). Arrow marks the approximated time in which odorant levels start to rise.C
Representative traces of wt, *tax‐6(p675)*, *and tax‐6(p675)* + *AWA:TAX‐6* worms (taken from the first row of each strain in panel A).D, E
To quantify the differences in activity between wt worms and the various mutants, we extracted individual pulses (see Materials and Methods). When compared with wt, number of pulses was significantly lower in *tax‐6(ok2065)*, *tax‐6(p675)*, *grk‐2*, *odr‐3*, and *osm‐6* mutants (P=0.002,0.002,0.005,0.002,0.007, respectively). Maximal amplitude was significantly higher in *tax‐6(ok2065)*, *tax‐6(p675)*, *eat‐16*, *grk‐2*, and *osm‐6* mutants (*P* = 0.002, 0.02, 0.02, 0.002, 0.003 respectively) and significantly lower in *odr‐3* mutants (*P* = 0.04). In both *tax‐6* mutant alleles (*ok2065* and *p675*), the number of pulses was significantly lower when compared with the rescued strain (P=0.005,0.004, respectively), and the maximal amplitude was significantly higher (P=0.002,0.004, respectively). Wilcoxon rank‐sum test, FDR corrected for the 20 comparisons. Red bar marks the median. *P < 0.05, **P < 0.01, ***P < 0.005. Source data are available online for this figure.

First, we analyzed the capacity of these mutant strains to generate pulsatile activity in response to smooth gradients of the stimulus diacetyl (Fig 3 and Appendix Fig S3). As expected, calcium transients were barely observed in *odr‐3* mutants as this protein is the immediate downstream component of the GPCR ODR‐10. Interestingly, robust loss of pulsatile activity was observed in *tax‐6* mutants (in both *p675* and *ok2065* alleles). These mutant animals exhibited a single pulse at the start of the gradient ramp, which then slowly decayed over the entire course of the experiment (Fig 3A–D and Appendix Fig S3B and C). A similar dynamics was observed in *grk‐2* mutants (although the effect was less prominent), and in some of the *eat‐16* and *osm‐6* mutants (though not as consistent as observed in the *tax‐6* mutant animals, Fig 3A and D and Appendix Fig S3E and I). In addition, the maximal amplitude of *tax‐6* pulses (as well as of *grk‐2*, *eat‐16*, and *osm‐6*) was significantly higher than the maximal amplitude observed in wild‐type worms (Fig 3E). Together, these results suggested that TAX‐6/Calcineurin and GRK‐2 play a key role in inhibiting signaling and reducing intracellular calcium levels, effectively terminating the pulsatile activity.

### TAX‐6/calcineurin cell‐autonomously promotes exact adaptation and habituation

**Figure 4:**
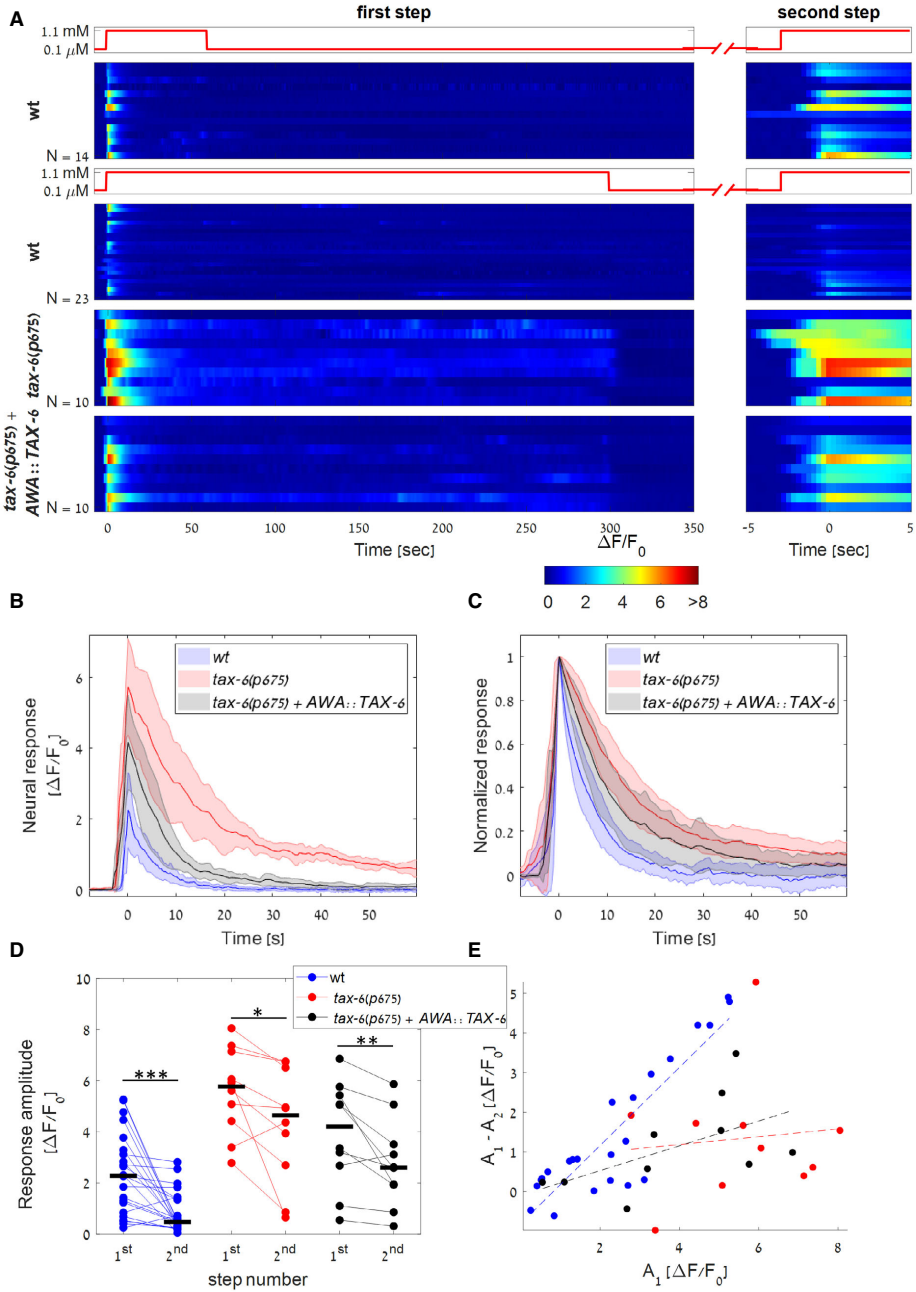
TAX‐6/Calcineurin is required for exact adaptation and habituation A
Response dynamics of wt, *tax‐6(p675)*, and *tax‐6(p675)* + *AWA::TAX‐6* worms to a two‐step protocol: First step consisting of 5 min (bottom panels, N=23,10,10 worms respectively) or 1 min (top panel, N=14 wt worms. Biological replicates) of on step, followed by a 2 min, or 6 min off step, respectively, before applying a second short step (right panels). Wt worms exposed to the 5‐min step showed stronger habituation compared to worms exposed to the 1‐min‐long step ($P=9\cdot 10^{-4}$, Wilcoxon rank‐sum test between the amplitudes of the second steps). Steps are aligned to the maximal amplitude (at time 0).B, C
Median neural activity (B) and normalized neural activity (C) of wt, *tax‐6(p675)*, *and tax‐6(p675)* + *AWA::TAX‐6* worms in response to the first step. Fitting the pulse decay with an exponent of the shape $a\cdot e^{-x/\tau }+c$, *tax‐6* mutants showed a longer decay time than wt worms, suggesting slower adaptation dynamics ($\tau_{\text{median}}$ of 13.5 and 5.6 s, respectively, Wilcoxon rank‐sum test, P=0.008). $\tau_{\text{median}}$ of the rescue line was 7.3 s, which was not significantly different from wt (Wilcoxon rank‐sum test, P=0.26). Color‐shaded area marks mean absolute deviation.D
Comparison of the amplitudes in the first and the second step for wt, *tax‐6(p675)*, *and tax‐6(p675)* + *AWA::TAX‐6* worms (following the longer 5‐min step). All three strains showed a weaker activity in the second step (signed‐rank test, $P=2.6\cdot 10^{-4},0.014,0.01$, respectively), with *tax‐6(p675)* worms having a significantly higher amplitude than wt in both the first and second steps (Wilcoxon rank‐sum test, $P=1.8\cdot 10^{-4}$ and $1.1\cdot 10^{-4}$ respectively). Black bars mark the median. *P < 0.05, **P < 0.01, ***P < 0.005.E
The difference between the response amplitudes of the first and the second steps as a function of the first‐step amplitude. While wt worms show a higher amplitude difference when the response to the first step is stronger ($r=0.89,\;P=1.3\cdot 10^{-8}$), *tax‐6(p675)* mutants do not (*r* = 0.1, *P* = 0.78), suggesting that calcium influx affects habituation in wt worms but not in *tax‐6(p675)* mutants. The linear dependency in the rescued animals (*tax‐6(p675)* + *AWA::TAX‐6*) is higher than in *tax‐6* mutants, though the correlation is not significant (r=0.56,P=0.09). Source data are available online for this figure.

As *tax‐6* mutants completely lost the ability to generate pulsatile activity in response to smooth gradients, we proceeded with this gene to analyze its capacity to reach exact adaptation, a hallmark of chemosensory coding. Furthermore, as the results for both mutant alleles were essentially similar, we proceeded with the *p675* allele only. To analyze the capacity to reach exact adaptation, we exposed the worms to a 5‐min long on‐step of diacetyl (Fig 4A, bottom panels). To analyze possible habituation, a reduction in the neural response between consecutive on steps (Larsch *et al*, 2015), we inflicted a second short on step following 2 min off step.

Following the first step, the response amplitude of the *tax‐6* mutants was significantly higher than that of wt worms, and it was followed by a significantly slower exponential decay (Fig 4B and C). Notably, the neural activity of eight out of the 10 *tax‐6* mutants did not return to its baseline levels for the entire duration of stimulus presentation, indicating a failure to perform exact adaptation (Fig 4A, bottom panels). Only when removing the stimulus (following 5 min of on step) did the activity resume to its basal level. Importantly, the failure to reach exact adaptation was not due to the enhanced neural activity since *eat‐16* mutants also showed enhanced responses to a sigmoidal gradient (Fig 3E) and yet, reached exact adaptation following an on‐step stimulation (Appendix Fig S4).

Furthermore, both the wt worms and the *tax‐6* mutants habituated to the stimuli, showing weaker responses to the second step (Fig 4D). However, only in wt worms this reduction positively correlated with the magnitude of the first pulse (Fig 4E). This suggests that initial stronger pulses, which lead to greater calcium influx, also increase TAX‐6‐mediated adaptation processes (Fig 4E and Appendix Fig S5). Therefore, as TAX‐6/Calcineurin activity is calcium‐dependent, calcium may provide the negative feedback required for the TAX‐6‐mediated adaptation.

We next asked whether factors other than calcium may also contribute to habituation. For this, we compared neural responses of wt animals following either a short or a long on step of the stimulus. The short on‐step stimulus essentially allowed the worms to be off the stimulus for a longer time period before inflicting a second on step (Fig 4A, top panel). While calcium dynamics in both protocols was similar in response to the first step, the response to the second step was significantly reduced following the long‐step protocol where the worms had a shorter time period to recover before the start of the second on step (Fig 4A, compare top two panels). Notably, in both protocols, calcium levels between the onset of the first and the second steps were similar. This suggests that a second, calcium‐independent adaptation mechanism may exist. An alternative explanation is that recovery from adaptation can only occur in the absence of an external stimulus. We found similar results when comparing activity dynamics in response to a multistep protocol and a single long‐step protocol (Appendix Fig S5).

TAX‐6 is widely expressed in the *C. elegans* nervous system (Kuhara *et al*, 2002; Taylor *et al*, 2021). We therefore asked whether TAX‐6 promotes exact adaptation and habituation in the AWA neurons cell‐autonomously or possibly via feedback from other neurons. For this, we introduced TAX‐6 expression in a *tax‐6* mutant (*p675*) exclusively in the AWA neurons (see Materials and Methods). Notably, the wt AWA dynamical features were restored: When facing a stimulus gradient, the pulsatile activity was reestablished (Fig 3A–D and Appendix Fig S3D), and the amplitude of these pulses was reduced to wt levels (Fig 3E). In response to a stimulus step, the heightened amplitude of *tax‐6* mutants was reduced to be closer to wt levels, and exact adaptation was observed in a fraction of the tested worms (Fig 4A–D). In addition, the correlation between habituation and the amplitude of the first pulse was partially restored, shifting the *tax‐6* response dynamics closer to that of the wt (Fig 4E). These results suggest that TAX‐6 acts cell‐autonomously in the AWA neurons to promote pulsatile activity, exact adaptation, and habituation between consecutive pulses.

### TAX‐6/calcineurin‐mediated inhibition does not affect the activity of voltage‐gated calcium channels

**Figure 5:**
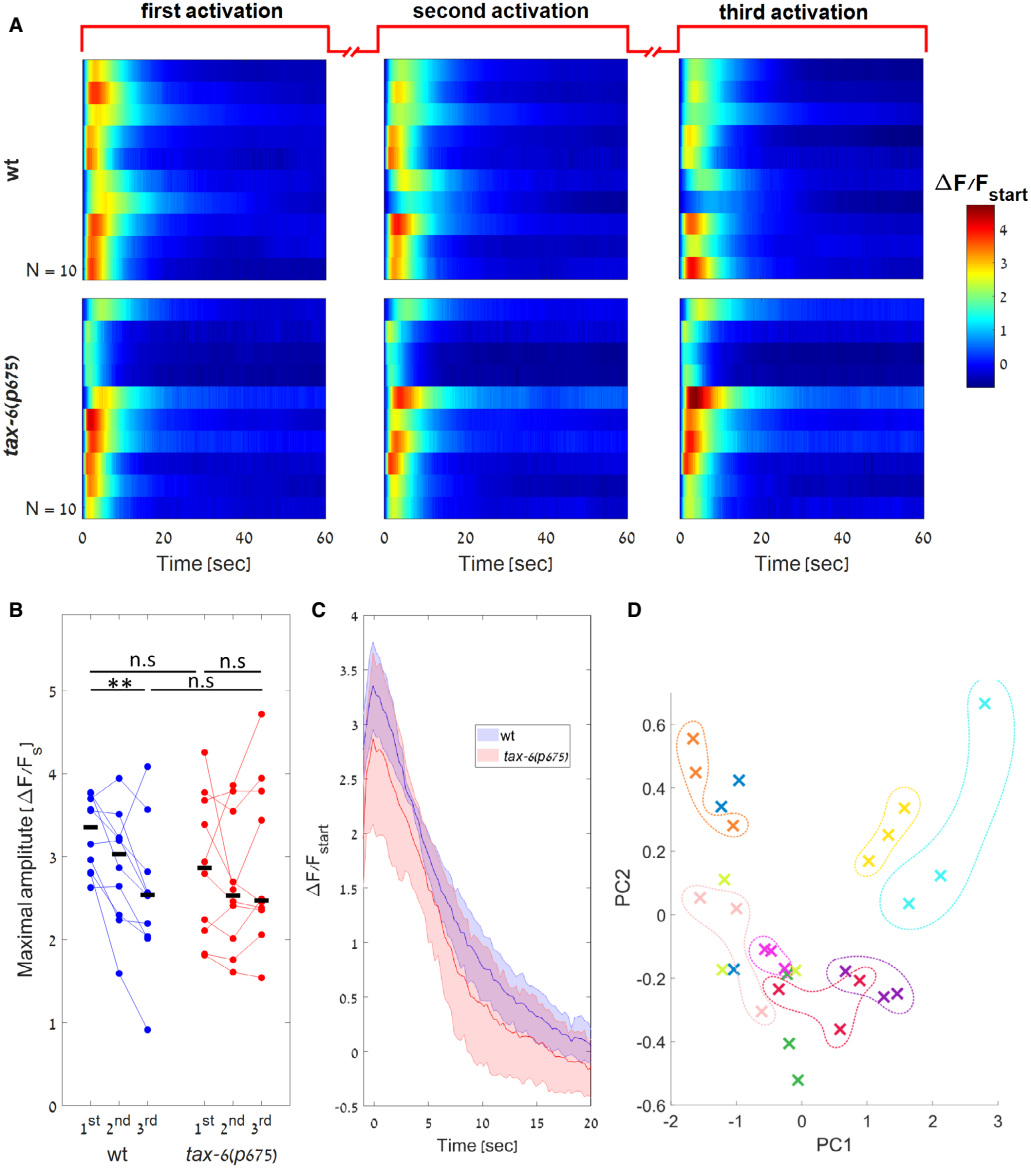
wt and *tax‐6* mutants show similar response dynamics following optogenetic stimulation A
AWA calcium dynamics of wt and *tax‐6*(p675) mutants (N=10,10 worms respectively) in response to three repeated light activations (488 nm). Each worm was illuminated thrice, each for 1‐min long with 1‐min rest between consecutive stimulations. Responses are shown as fold differences in fluorescence from the first captured frame.B
Peak amplitudes as measured for each of the activations. Following light activation, and in contrast to the response to diacetyl, response of *tax‐6* mutants was not stronger than the response of wt worms (P=0.27,0.47 Wilcoxon rank‐sum test between the amplitudes of wt and *tax‐6*(*p675*) in response to the first or third light activation, respectively). Black bars mark the median. Horizontal lines denote the compared groups. *P < 0.05, **P < 0.01, ***P < 0.005.C
Median response dynamics of wt and *tax‐6*(*p675*) worms for the first light activation. The two strains show similar activation dynamics. All responses were aligned to their maximal activation levels prior to extraction of the median values. Shaded‐colored area marks mean absolute deviation.D
Activations from the same worm tend to cluster together indicating a low in‐worm variability, while the between‐worm variability is high. The first 30 s in each of the three light activations of the 10 wt worms shown in (A) were normalized by the maximal amplitude and decomposed using Principal Component Analysis (PCA). Shown are the projections of each activation to the first two PCs (capturing 90% and 6% of the variance, respectively). Each color represents a single worm (three activations for each worm). Colored dashed lines were added manually. Source data are available online for this figure.

The response dynamics of *tax‐6* mutants to diacetyl was significantly different than that of wt worms: they exhibited a heightened single calcium pulse, which did not resume to basal levels (hence no exact adaptation). To elucidate how the lack of TAX‐6 inhibition leads to the differential response, we subjected the worms to artificial light‐induced activation using the optogenetic channel Chrimson (Fig 5A). This light activation increases calcium levels that directly affect VGCC opening and thus bypasses the natural activation and signaling through the ODR‐10 GPCR and the TRPV channels (Fig 1A).

For this, neurons were light‐activated thrice, each time for 1‐min long, while simultaneously imaging calcium dynamics. During the continuous 1‐min long optogenetic activation, both wt and *tax‐6* worms showed a similar fast (∼2 s) increase in calcium levels, which was followed by a slow gradual decrease (Fig 5A). The second and the third light activations elicited responses that were similar to the responses observed after the first light activation, though with a mildly lower amplitude, which may be attributed to bleaching of the fluorescent signal (Fig 5A and B). In fact, both wt and *tax‐6* mutants showed similar neural dynamics in response to light activations (Fig 5C). This is in stark contrast to their differential responses to the natural stimulus diacetyl (Fig 4B and C). Since calcium influx mediated by repeated light‐induced activations does not lead to adaptation, this suggests that TAX‐6/Calcineurin mediates calcium feedback inhibition by affecting the signaling cascade between the ODR‐10 receptor (including) and the TRPV channels (see Fig 1).

Another interesting observation is the relative heterogeneity in the responses of individual worms to the light stimulus. While the response pattern of each worm seems highly repetitive, the fine activation dynamics in the different worms varied with respect to the amplitude, the rise time, and decay kinetics (Fig 5A). To visualize the differences of individual responses, we normalized each response by its maximal amplitude and used Principal Component Analysis (PCA) to project each activation pattern of the wt worms on the two main PCs (Fig 5D). Indeed, the three activations of each worm cluster together, indicating a low in‐worm variability and high between‐worms variability. Notably, these idiosyncratic responses were observed in an isogenic population of worms, expressing the same levels of Chrimson (chromosomally integrated), and grown under the exact same conditions. Thus, the animals' cell‐intrinsic state (e.g., expression levels of the different components, channel's functional states) may govern the shape of the response dynamics.

### A GPCR negative feedback model recapitulates fine dynamic responses of wt and *tax‐6* mutants

The above experiments position calcium and TAX‐6/Calcineurin as key components for GPCR inhibition and exact adaptation. We therefore turned back to our parsimonious model (equations (1), (2), (3), (4) and Figs 1 and 2) and asked whether elimination of TAX‐6/Calcineurin from the circuit will recapitulate the experimental results.

To simulate dynamics of *tax‐6* mutants, we nullified the calcium‐dependent term from equation 4 in the original model (by setting $k_{5}=0$). Hitherto, for simplicity purposes and to allow analytical tractability of the model (Fig 1 and equations (1), (2), (3), (4)), we did not include explicit description of the voltage‐gated channels dynamics in AWA. These details were not crucial for modeling dynamics in wt animals, where the negative feedback was the major drive for exact adaptation. However, in *tax‐6* mutants that lack the negative feedback, the role of the voltage‐gated ion channels in reducing calcium levels becomes prominent. Thus, for completeness, we have now included in the model a module that provides the fine details for the initiation and termination of the pulses within the AWA neurons. This module was implemented exactly as it appears in (Liu *et al*, 2018), who experimentally measured intracellular currents (see details in Appendix Note S1 part 4).

**Figure 6:**
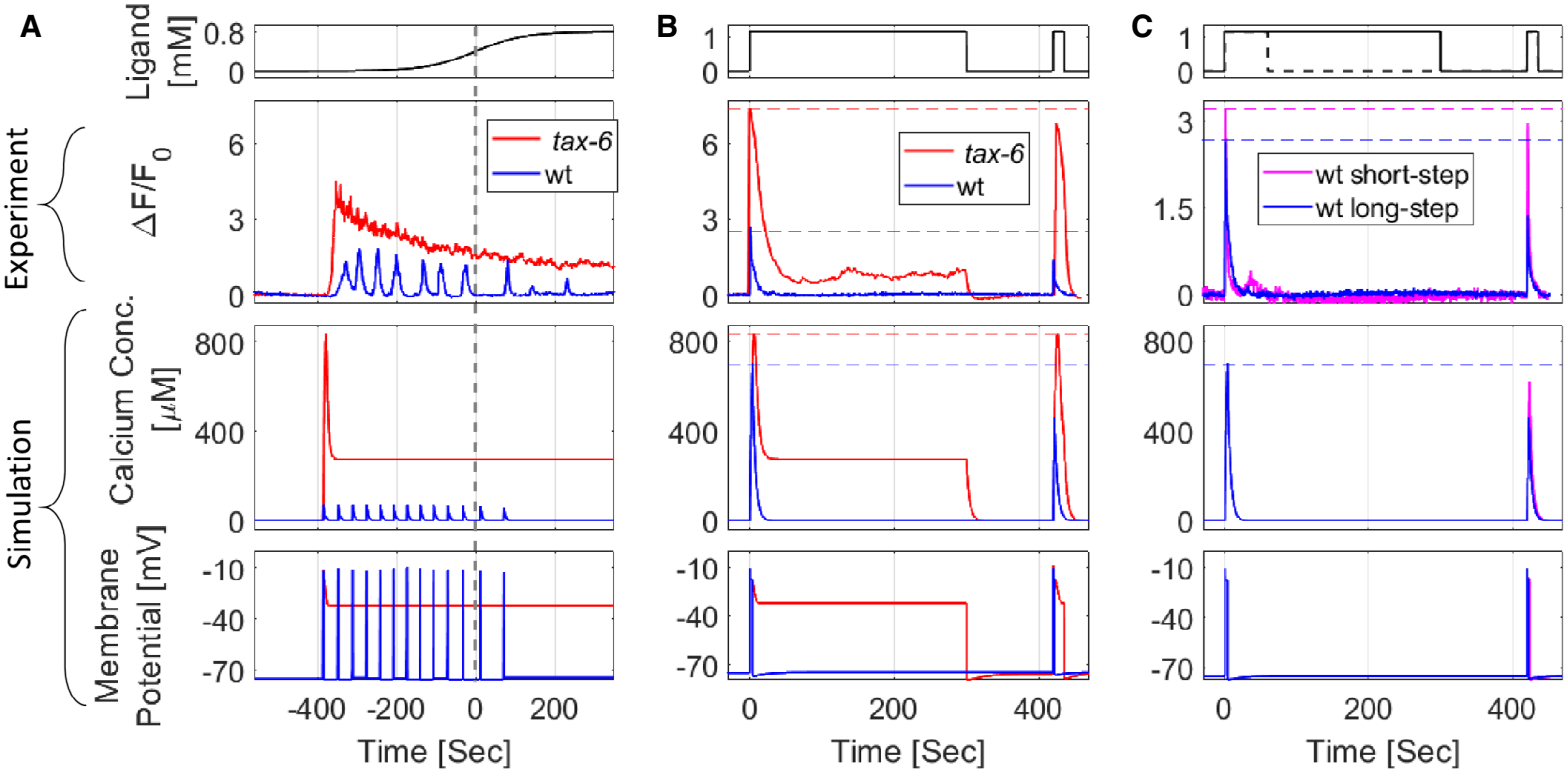
A detailed mathematical model recapitulates experimental results of both wt and *tax‐6* mutant worms In the detailed model (see Appendix Note S1 part 4), we integrated the available dynamics of the AWA ion channels (Liu *et al*, 2018). To simulate circuit dynamics of *tax‐6* mutants, we set the contribution of calcium‐mediated inhibition to zero ($k_{5}=0$ in equation 4 and shown in Fig 1B). A–C
Calcium concentration and membrane potential as simulated by the model, alongside calcium imaging measurements of a representative worm in response to different stimulation patterns (top). (A) In response to a sigmoidal gradient, simulated *tax‐6* worms fail to generate pulsatile activity, in agreement with the experimental results. Also, activity does not resume to its baseline levels. (B) Response dynamics following a first long on step (identical to experiment protocol described in Fig 4). Experimental and simulated wt worms exhibit reduced amplitude responses (increased habituation) following the second on step. In contrast, *tax‐6* mutants did not show exact adaptation nor a reduced activity in response to the second on step (no habituation). (C) Response dynamics of wt worms following a first long (continues line) or short (dashed line) on step. A second on step following a short on step (and hence a long off step), elicits calcium levels that match the first step (purple). This contrasts with the attenuated response following a long on step (and hence short off step, blue). Even though in both protocols, calcium concentrations returned to baseline levels, the longer the off step, the higher the response activity to a second step (in agreement with experimental results, Fig 4A), suggesting that calcium‐independent processes contribute to the inhibition. Note the different *y*‐scale for the experimental results in panels (B) and (C). Source data are available online for this figure.

The resulting simulations recapitulated the experimental results. When exposed to increasing gradients of the stimulus, wt animals exhibited a pulsatile activity that adapted to the magnitude of the gradient's first derivative (Fig 6A). In contrast, activity of *tax‐6* mutants showed a single pulse whose amplitude was higher than that of the wt worms. However, our simplified model failed to accurately describe the slow decay of the pulse in *tax‐6* mutants, probably due to our oversimplification of the two‐states self‐amplification process (equation 2).

When considering the responses to discrete on steps of the stimulus, our simulation qualitatively captured the observed experimental results (Fig 6B and C). Wt animals exhibited a single pulse whose dynamics returned to the basal level (exact adaptation), while *tax‐6* mutants responded with a single pulse that was higher in amplitude and which remained above basal level for as long as the stimulus remained on. This heightened amplitude of *tax‐6* mutants was also observed following the second step, whereas the response of wt worms to the second step was reduced, suggesting that *tax‐6* mutants fail to habituate to past experienced stimuli (Fig 6C, Appendix Fig S6).

Finally, to verify that our model also captures calcium‐independent inhibitory processes, we compared the simulated and the experimental response dynamics of wt worms in the two consecutive on‐step stimulation protocols (short and long intervals, Figs 4A and 6C). While calcium responses to the first on step were identical in both protocols, the amplitude of the second pulse, following a short off‐step interval, was lower (Fig 6C), in agreement with the experimental results (Fig 4A). Notably, the lower amplitude observed following the second step was despite the fact that calcium levels already resumed to baseline levels. This suggests that inhibition is also affected by calcium‐independent inhibition processes.

Taken together, our parsimonious model captured the key features that were experimentally observed: calcium‐dependent and calcium‐independent inhibition, exact adaptation, habituation, and the corresponding dynamics in *tax‐6* mutants, which lack inhibitory capacity. Notably, these features robustly appear despite the model's large parameter space and the various possible outcomes.

## Discussion

In this work, we demonstrated how a simple negative feedback loop in the GPCR signaling pathway promotes efficient coding of complex stimulus patterns. Moreover, we showed how this coding is achieved cell‐autonomously and identified Calcineurin/TAX‐6 as key for the negative feedback. A simple mathematical model recapitulated the experimental results observed in both wt and a calcineurin‐deficient mutant strain, further underscoring the validity of the model and the interpretation of the experimental results.

Notably, the GPCR negative feedback supports several important computational features, including: (i) coding ligand concentration on a logarithmic‐like scale, thus enabling adjusted responses across several orders of magnitude of the stimulus. (ii) Responding with a single pulse to a step function and with multiple pulses when presented with a continuous increasing gradient of the stimulus. (iii) Exhibiting exact adaptation even when the stimulus remains on. (iv) The mean frequency and the amplitude of the pulses correlate with and adapt to the magnitude of the gradient's first derivative. It is therefore remarkable that all these features can be achieved cell‐autonomously with a simple negative feedback loop.

**Figure 7:**
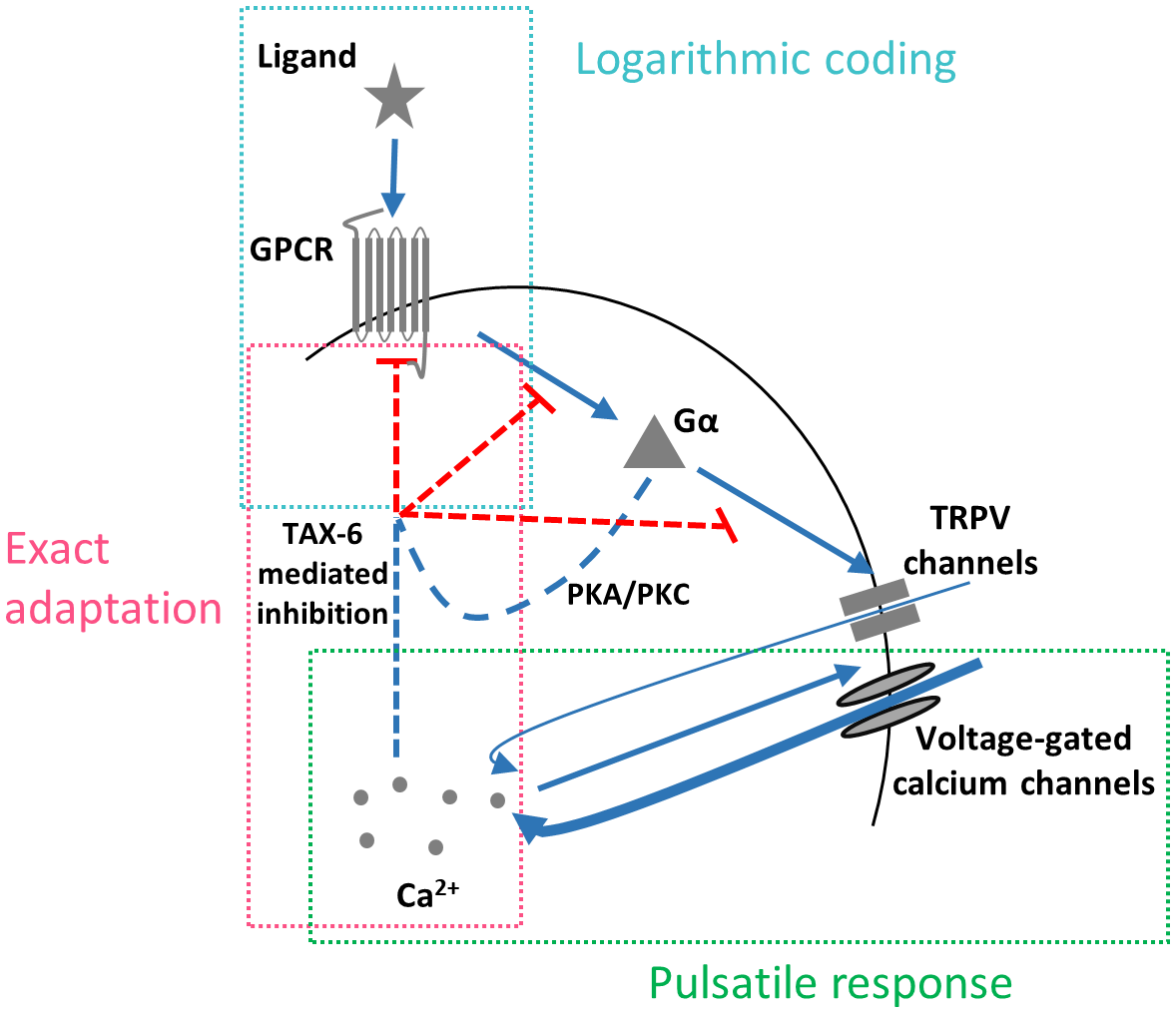
The negative feedback loop can be decomposed into discrete modules where each module fulfills a defined computational role Logarithmic coding is achieved by the logarithmic transformation of ligand levels to receptor's activity, which in turn is linearly inhibited by the system's output (calcium levels). Exact adaptation is achieved by the integral feedback where the system's output inhibits receptor activity in a calcium‐dependent manner. The self‐amplifying activity of the TRPV and VGCC channels underlies the pulsatile activity. TAX‐6/Calcineurin may be acting as a negative feedback on any of the processes marked in red dashed line.

These features are embedded in the signaling pathway in a modular fashion (Fig 7). Adaptation to the first derivative of the gradient and logarithmic‐like coding are achieved by the inherent activity mode of GPCRs (Olsman & Goentoro, 2016). These receptors are logarithmically facilitated by the ligand, but linearly inhibited by intracellular components. Exact adaptation is achieved by calcium‐mediated inhibition. As calcium levels can be viewed as the circuit output, their feedback inhibition forms an integral feedback that underlies exact adaptation, in analogy to the exact adaptation observed in the *E. coli* chemotaxis system (Barkai & Leibler, 1997; Yi *et al*, 2000; Tu *et al*, 2008). Pulsatile coding of smooth gradients is driven by a self‐excitatory element, which converts the graded response into a series of pulses. As the negative feedback loop links all these features together, removing a single element, such as TAX‐6/Calcineurin, disrupts all these functions.

Clearly, this negative feedback necessitates that all the components will be localized to the cilia, the dendrite ending region. Indeed many of the components had been shown to be localized to the dendrites and cilia of *C. elegans* sensory neurons, including the ODR‐10 GPCR, the Gα subunit ODR‐3, the VGCC EGL‐19, and TAX‐6/Calcineurin (Sengupta *et al*, 1996; Roayaie *et al*, 1998; Kuhara *et al*, 2002; Mukhopadhyay *et al*, 2008; Shindou *et al*, 2019). Supporting this hypothesis, we observed calcium pulsatile activity in the AWA dendrite and cilia activity, which was synchronized with the calcium transients observed in the cell soma (Movie EV1).

We discovered that TAX‐6/Calcineurin serves as a key component in inhibiting GPCR signaling: while *tax‐6* mutants were unable to generate pulsatile activity and reach exact adaptation in response to the natural stimulus diacetyl (Figs 3 and 4), their calcium dynamics following optogenetic activation was similar to wt worms (Fig 5). This positions the TAX‐6/Calcineurin to mediate inhibition along the pathway between the GPCR to the TRPV (Figs 1 and 7). Importantly, TAX‐6 mediates inhibition cell‐autonomously: when expressing TAX‐6 exclusively in the AWA neurons of *tax‐6* mutants, we were able to rescue exact adaptation and pulsatile response (Figs 3 and 4). This rescue was observed in a fraction of the animals, possibly due to the variable expression levels of TAX‐6 that is expressed from an extrachromosomal array. This is also in line with a previous report showing that TAX‐6 can cell‐autonomously control adaptation in a number of sensory neurons and modalities (e.g., the thermosensory AFD neurons, the polymodal ASH neurons, and in the olfactory AWC neurons; Kuhara *et al*, 2002). For example, *tax‐6* mutants are hyper‐adaptive to odorant pre‐exposure and AWC‐specific rescue restores this phenotype. Thus, our observations that *tax‐6* mutants show long‐lasting calcium fluxes that fail to resume basal pre‐stimulus levels may explain the hyper‐adaptation phenotypes scored via behavioral outputs (Kuhara *et al*, 2002). This long‐lasting pulse also prevents formation of sequential pulses, presumably rendering the AWA neurons insensitive to the gradient, thus leading to defective chemotaxis (Movies EV2 and EV3 and Appendix Fig S7).

How could TAX‐6/Calcineurin promote inhibition? Calcineurin is a phosphatase, thus it may indirectly mediates phosphorylation of active GPCRs (Creamer, 2020). Such feedback, where the active receptors that initiate the signaling cascade are also modified, is known as homologous adaptation. For example, homologous adaptation was shown in the *C. elegans* AWC neuron, where adaptation to one odorant did not lead to adaptation to a second odorant, which is sensed by the same neuron, presumably because it signals through a different receptor (Colbert & Bargmann, 1995). In *C. elegans*, GPCR phosphorylation is presumably carried out by *grk‐1* and *grk‐2* (which may work redundantly, as a mutation in *grk‐2* alone does not lead to reduced adaptation nor to hypersensitivity; Fukuto *et al*, 2004), as well as by the calmodulin‐dependent protein kinase II homolog *unc‐43* (Hobert, 2013). Interestingly, phosphorylation of active receptors is thought to underlie the logarithmic coding of GPCRs (Olsman & Goentoro, 2016).

It was previously demonstrated that exact adaptation can be achieved when the output signal directly inhibits the sensor in a negative feedback loop (Barkai & Leibler, 1997; Tu, 2013). As calcium drives the main synaptic output of neurons (Katz & Miledi, 1970; Llinás *et al*, 1976), it constitutes a preferable candidate to mediate adaptation via a calcium‐binding enzyme, such as TAX‐6/Calcineurin. The link between calcium and inhibition is further supported by the correlation between calcium influx and the magnitude of the habituation (Fig 4E). Interestingly, a similar correlation between calcium levels and habituation was also observed in mice (Vinograd *et al*, 2017), suggesting that similar principles may underlie these processes in higher organisms. As habituation to the stimulus persisted even after calcium levels returned to their baseline levels (Fig 4A), a second, calcium‐independent inhibitory process may also exist.

To better understand the key principles that promote these coding dynamics, we constructed a simple mathematical model that captured key dynamics features of both wt and *tax‐6* mutant animals. Notably, rather than accurately fitting the experimental results, the main purpose of this model was to demonstrate how a calcium‐mediated feedback inhibition in the GPCR signaling pathway can robustly explain an array of sensory responses: It enables pulsatile activity as well as adaptation to the first derivative of the gradients; it is responsible for exact adaptation in response to step changes; and it mediates habituation between sequential presentations of the same odorant cue.

Another model for neural activation was proposed by Levy and Bargmann, known as the adaptive‐threshold mechanism (Levy & Bargmann, 2020). According to this mechanism, the threshold for initiating a neural response in the AWC sensory neurons is not fixed, but rather a dynamic variable that changes depending on the history of the perceived stimulus. Our experimental design and modeling results, which considered smooth continuous gradient stimuli, carry such stimulus history, and with few assumptions, our model is mathematically analogous to the adaptive‐threshold model (A detailed mathematical derivation is found in Appendix Note S1 part 5).

When constructing the model, we strived to simplify the detailed signaling cascades, focusing on the key computational components that underlie the observed coding. For this, we grouped components acting together or sequentially and abstracted parts of the system's dynamics. For example, we modeled activities of TRPVs and VGCCs as a single self‐activating component. As AWA neurons generate action potentials (Liu *et al*, 2018), a more realistic model would include the positive feedback loop between the VGCCs, the potassium channels driving the action potential downstroke, and the membrane potential (dictated in part by calcium). It is conceivable that the calcium pulses may be initiated as a result of a spike train resulting from voltage‐dependent conductance. However, these spikes operate on shorter timescales than the calcium transients and the longer temporal decay of intracellular calcium (on the order of tens of seconds, Fig 3) may be crucial for the negative feedback of the GPCR pathway. This longer timescale also dictates behavioral outputs, as animals maintain a straight trajectory during the pulse when AWA neurons are active and turn once the pulse is terminated (Itskovits *et al*, 2018).

Interestingly, the activation dynamics in individual animals considerably varied, even in response to identical highly reproducible optogenetic activations (Fig 5). This variability is in line with previous reports demonstrating significant differences between individual worms in neural activity patterns and behavioral outputs (Luo *et al*, 2014; Gordus *et al*, 2015; Stern *et al*, 2017; preprint: Desrochers *et al*, 2018; Itskovits *et al*, 2018; preprint: Pritz *et al*, 2020). Notably, the assayed worms were isogenic and were grown in the exact same conditions. The fact that these neural responses are cell‐autonomous (Itskovits *et al*, 2018) may suggest that a significant variability between individual animals is due to differences in cell‐specific internal states (Marder & Goaillard, 2006). Nevertheless, despite the inherent noise and the significant differences in response dynamics, all neurons showed exact adaptation and pulsatile activity. This may suggest cell‐autonomous mechanisms that provide robustness in face of noise and variability (Alon *et al*, 1999). In that respect, the mathematical model suggests that this robustness may be inherently encoded in the negative feedback as the neural outputs were largely insensitive to a wide range of changes in the system's parameters (Appendix Fig S2).

Together, here we delineated how a negative feedback in the GPCR signaling pathway provides an array of key sensory features that together underlie an efficient navigation strategy. We identified TAX‐6/Calcineurin as a major cell‐autonomous inhibitory component and constructed a simple mathematical model that depicts the experimental observations in both wt and *tax‐6* mutant animals. While this model simplifies the full dynamics within the neuron, it establishes a convenient framework to understand the principles by which a neuron translates complex stimulus patterns into coherent activity outputs. As GPCR signaling is conserved in all eukaryotes, the same mechanisms may underlie cell‐autonomous coding schemes in different sensory modalities across the animal kingdom.

## Materials and Methods

### Strains used in this study

ZAS163 [syEx1252(*gpa‐6*::GCaMP3, *pha‐1*::PHA‐1); *pha‐1(e2123ts)*; *lite‐1(ce314)*].

The following strains were crossed with ZAS163:

PR675 [*tax‐6*(p675) IV] yielding the strain ZAS282

RB1667 [*tax‐6*(ok2065) IV] yielding the strain ZAS486

PR811 [*osm‐6*(p811) V] yielding the strain ZAS416

JT609 [*eat‐16*(sa609) I] yielding the strain ZAS403

RB660 [*arr‐1*(ok401) X] yielding the strain ZAS402

CX2205 [*odr‐3*(n2150) V] yielding the strain ZAS482

FG7 [*grk‐2*(gk268) III] yielding the strain ZAS487

ZAS423 [*azrIs423*(*odr‐7*p::Chrimson::SL2::mCherry; *elt‐2*::mCherry); *syEx1252*(*gpa‐6*::GCaMP3, *pha‐1*::PHA‐1)] was generated by first integrating CX16561(*kyEx5662*[*odr‐7*p::Chrimson::SL2::mCherry 5 ng/ul, elt‐2::mCherry]) (Larsch *et al*, 2015) using UV and backcrossing seven times with wt N2 worms. The resulting strain was then crossed with ZAS163. As GCaMP3 signal was weaker in worms homozygous for the Chrimson, heterozygous worms were picked for imaging.

ZAS424 [*tax‐6*(*p675*); *azrIs423*(*odr‐7*p::Chrimson::SL2::mCherry; *elt‐2*::mCherry); *syEx1252*(*gpa‐6*::GCaMP3, *pha‐1*::PHA‐1)] was generated by crossing ZAS423 with PR675[*tax‐6*(*p675*)], which yields the genotype *azrIs423*(*odr‐7*p::Chrimson::SL2::mCherry; *elt‐2*::mCherry); *syEX1252*(*gpa‐6*::GCaMP3, *pha‐1*::PHA‐1) in *tax‐6* mutant background.

ZAS489 [*tax‐6(p675); lite‐1(ce314); pha‐1(e2123)*; *syEX1252*(*gpa‐6*::GCaMP3; *pha‐1*::PHA‐1); *azrEx489*(*gpa‐6*::TAX‐6‐DsRed; *unc‐122*::GFP)] was generated as following: The TAX‐6 coding sequence was amplified from N2 cDNA library using the following primers: forward ‐ ATGGCCTCGACATCGGC; reverse ‐ TTAGCTATTTGATGGACCATTTTG. It was then cloned into the pPD95.77 plasmid upstream and in frame to DsRed. Next, we fused the pcr segment of TAX‐6‐DsRed from the pPD95.77 plasmid to the *gpa‐6* promoter amplified from a genomic DNA (Zaslaver *et al*, 2015; Bokman *et al*, 2022). Finally, we injected the linear fragment to ZAS282 together with *unc122*::GFP. The resulting transgenic animals showed DsRed AWA‐exclusive expression in the head and in another cell in the tail.

Worms were grown at 20°C on NGM plates that were pre‐seeded with overnight culture of OP50 according to (Sulston & Brenner, 1974).

### Preparing reagents for calcium imaging

For calcium imaging of the AWA neurons in response to diacetyl, we used two types of solutions: “Stimulus” and “Buffer.” Both solutions were composed of a CTM buffer supplemented with 10 mM Levamisole (Sigma, CAS Number: 16595‐80‐5). Levamisole was used to minimize worms' movement in the microfluidic device during imaging. Importantly, Levamisole does not alter AWA response activities as non‐sedated worms demonstrate similar pulsatile activity (Itskovits *et al*, 2018). The “Stimulus” input was also supplemented with 1.15 mM diacetyl and 0.5 μM rhodamine. The rhodamine dye was used to directly measure the diacetyl gradient experienced by the worms. Importantly, the AWA neuron does not respond to these concentrations of rhodamine (Itskovits *et al*, 2018). We also added low levels of diacetyl (0.12 μM) to the “Buffer” solution to reduce the effect of possible abrupt changes in diacetyl concentration that would greatly impact AWA activity.

### Calcium imaging setup

We typically imaged one (either left or right) of the bilateral symmetric AWA neurons using an Olympus IX‐83 inverted microscope equipped with a Photometrics sCMOS camera (Prime 95B) and a 40× magnification (0.95 NA) Olympus objective. A dual‐band filter (Chroma 59012) and a two‐led illumination source (X‐cite, Lumen Dynamics) were used to allow alternating imaging of both green and red channels intermittently. Hardware was controlled using Micro‐Manager (Edelstein *et al*, 2014).

### Generating gradients and steps of the stimulus

To generate smooth gradients of diacetyl, we used two computer‐controlled syringe pumps to flow the diacetyl and a diluting buffer into a small‐volume (50 μl) mixing chamber (Itskovits *et al*, 2018). The content of the chamber was stirred by a magnetic bead and its output was flowed through the nose of the worm while it was constrained in a custom‐made microfluidic device. Generation of step changes were done in an “olfactory chip” (Chronis *et al*, 2007). Switching between on and off steps was done either manually or using an automated valve (LFYA1218032H, the Lee company) controlled by Arduino.

### Image analysis

AWA activity (green) and rhodamine concentration (red) were each imaged at a rate of 1.4 frames/s. For step gradients that did not necessitate continuous measurements of diacetyl concentrations, we imaged the green channel at a frame rate of 3.3 frames/s. Image analysis was done using in‐house developed MATLAB scripts. We extracted the fluorescence values of the neuron at each time and calculated the change in fluorescence relative to the fluorescence before the gradient onset (or before the step): $\left(F-F_{0}\right)/F_{0}$ (as in Figs 2, 3, 4). Normalization of raster plots to a range of [0–1] was done using [val – min (val)]/[max (val) – min (val)].

### Optogenetic activation

About 12–24 h prior to imaging, L4 worms were picked and placed on NGM plates pre‐seeded with *E. coli* OP 50 supplemented with 100 μM ATR. Optogenetic activation was done via a 40× magnification (0.95 NA) Olympus objective by an X‐cite blue led illumination source set to 5% of maximal power. As the same LED light source was also used to image calcium levels, the beginning of neural activation was likely to be observed in the first recorded frame. We therefore quantified neural activity by comparing fluorescent levels to the first frame $\left(F-F_{\text{start}}\right)/F_{\text{start}}$.

### Extraction of the pulsatile activity in smooth gradients

Pulses were extracted automatically using the following criterion: a local maximum with an amplitude that exceeds 20% of the maximal measured fluorescence, and which is surrounded by a decay of at least 70% of its amplitude. Data points that obeyed this criterion were considered as pulses. In case no point passed this criterion, the number of pulses was set to 1.

### Model construction and parameter evaluation

We used MATLAB (Mathworks© Inc.) for implementing numerical simulations of the dynamical system (equations (1), (2), (3), (4) and Appendix Note S1 part 1). As we did not fit the model to the data, but rather aimed to construct a “toy” model that captures the fine dynamic features observed experimentally, some of the parameters were manually varied by orders of magnitude (see full description in Appendix Table S1). Parameters for which we had experimental evidence were fixed based on available data. Appendix Table S1 provides the description of the parameters and how they were evaluated.

### Parameter' scan and analysis of the model robustness

To analyze the robustness of the model to variations in the parameters space, we used two complementing approaches to simulate the model's output while varying the parameters: (i) we systematically varied the parameters one by one in the range of 0.1–10‐fold of their initialized value (100‐fold range in total). (ii) In each simulation, we simultaneously varied multiple parameters (except for $R_{t}$, whose dynamic range was found to be relatively small, as seen in Appendix Fig S3). Each parameter was drawn independently from a log‐uniform distribution, with a range that was set to generate a symmetric 10‐fold range around the initially set value (shown in Appendix Table S1). We used this process to generate 10,000 independent sets of parameters.

For each set of parameters, the model was tested for two features: (i) exact adaptation in response to a single step (1.15 mM) of the stimulus. Here, the output criterion was to observe a single pulse whose duration was less than 60 s. (ii) Adaptation of the pulsatile response to the magnitude of the gradient's first derivative (during a sigmoidal smooth gradient as shown in Fig 1C). The output criterion was to observe anywhere between 3 and 100 pulses, where more than 55% of the pulses initiated prior to reaching the middle point of the sigmoid gradient (the point of maximal first derivative, as expected for adaptation to the gradient's first derivative).

Pulses were extracted from the simulation output using the following criteria: a local maximum with an amplitude that exceeds 1% of the global maximal amplitude. A pulse's initiation/termination time was calculated as the time that the output rose/fell more than 10%/90% of its amplitude, respectively. Parameter values that produced activity dynamics obeying these criteria were considered as valid, thus leading to robust behavioral output of the model. To analyze the model's performance in response to various ligand concentrations, we performed each of the above simulations independently while modifying the stimulus amplitude only (the other parameters remained fixed at their initially set values).

### Chemotaxis assays

Assays were performed on synchronized‐bleached young adult (65–70 h post bleaching) wt and *tax‐6*(*p675*) worms. Worms were grown on standard NGM plates, pre‐seeded with 500 μl *E. coli* OP 50 culture. Before the experiments, the worms were rinsed off the growth plates and washed three times in CTM (5 mM potassium phosphate, pH 6.0, 1 mM CaCl_2_, 1 mM MgSO_4_) to remove bacteria. The worms were then kept food‐deprived in CTM for 45–60 min before loaded on the assay plates. Assays were performed in 9 cm covered CTX plates (25 mM KH_2_PO_4_, pH 6.0, 1 mM CaCl_2_, 1 mM MgSO_4_, 1.7% agar).

We drew a diameter on the plate's lid (90 mm round plates) and marked on it two points, ∼10 mm equally distant from the lid's edges. We used an agar chunk soaked (15 μl) with diacetyl (11.5 mM) and attached it from the inside of the lid, on one of the points that we marked. On the other point, on the CTX itself, we placed a 15 μl CTM drop of washed worms (100–200, Appendix Fig S7). Chemotaxis assays were imaged using a Photometrics Micropublisher 5 MB camera, using Olympus SZ61 binocular equipped with a 0.5× lens. Movies were acquired at a rate of two frames per second. To minimize day‐to‐day variability, we assayed wt and *tax‐6* mutants side by side in parallel. Image analyses and extraction of tracks were done using in‐house tracking software (Itskovits *et al*, 2017) that was modified to segment worm entities using a deep learning algorithm (available in https://github.com/itskov/WormSegmentation.git).

## Author contributions

**Rotem Ruach:** Resources; data curation; software; formal analysis; validation; investigation; visualization; methodology; writing – original draft; writing – review and editing. **Shai Yellinek:** Resources; data curation; software; formal analysis; investigation; visualization; methodology; writing – original draft; writing – review and editing. **Eyal Itskovits:** Data curation; software; formal analysis; validation; investigation; methodology. **Noa Deshe:** Resources; validation; methodology. **Yifat Eliezer:** Resources; validation; methodology. **Eduard Bokman:** Formal analysis; methodology. **Alon Zaslaver:** Conceptualization; data curation; supervision; funding acquisition; validation; visualization; writing – original draft; project administration; writing – review and editing.

## Disclosure and competing interests statement

The authors declare that they have no conflict of interest.

## Supporting information



Appendix

Movie EV1

Movie EV2

Movie EV3

Source Data for Figure 2

Source Data for Figure 3

Source Data for Figure 4

Source Data for Figure 5

Source Data for Figure 6

Source Data for Appendix

## Data Availability

The code for the model and the simulations is available in: https://github.com/zaslab/negative_feedback.git.
