# Supplementary material for: A negative feedback loop in the GPCR pathway underlies efficient coding of external stimuli
Source: Mol Syst Biol. 2022 Sep 15;18(9):e10514. doi: 10.15252/msb.202110514 (PMC9476886; doi:10.15252/msb.202110514)
Supplement: Supplementary file 1 — Appendix [file MSB-18-e10514-s007.pdf]

Appendix

**Appendix Figures S1-7**	**2-14**
**Appendix Table S1**	**15**
**Appendix Note S1 parts 1-5**	**16-22**
**References**	**23**

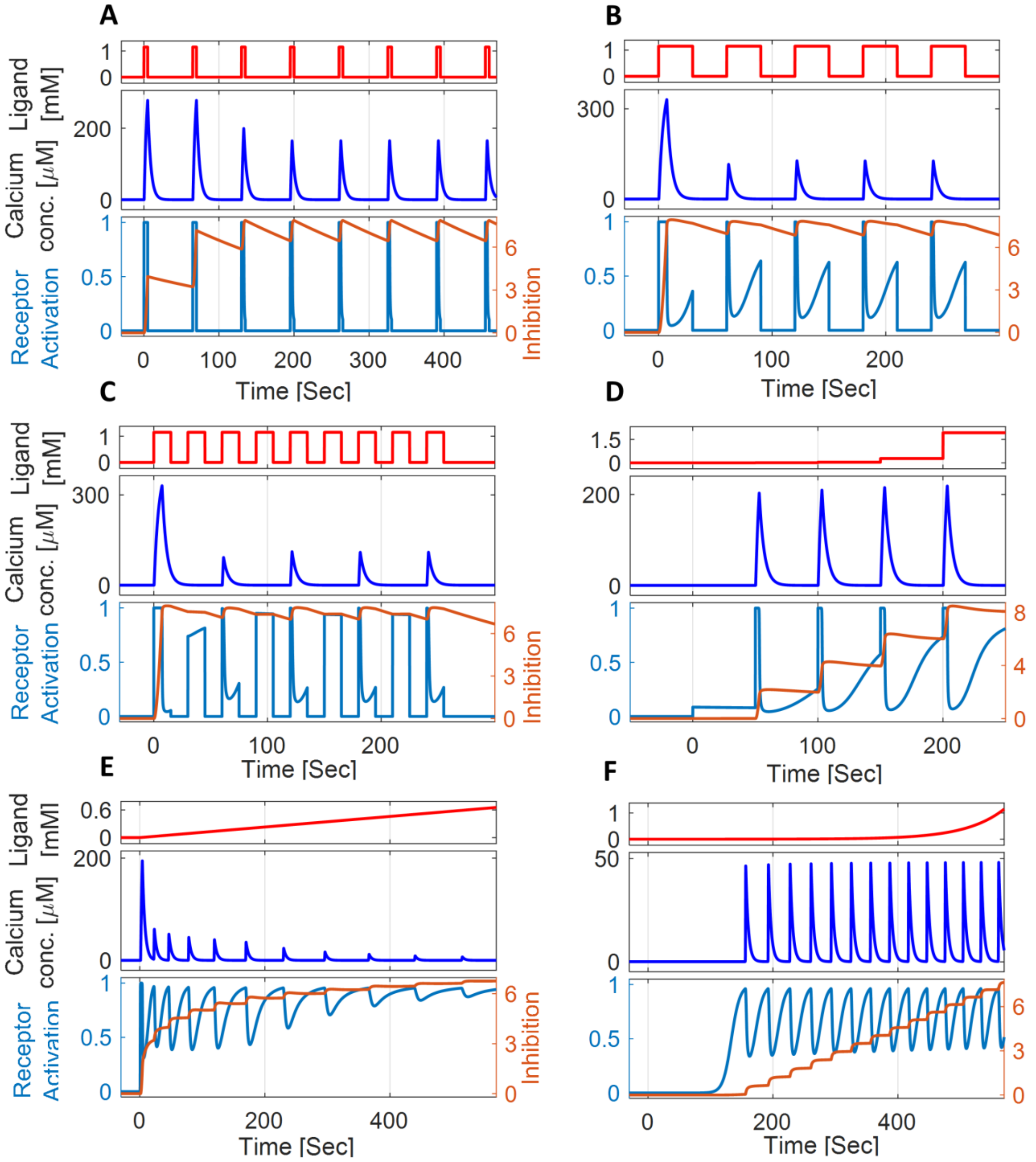

Appendix Figure S1. **Simulations of the circuit dynamics in response to various stimulation patterns.**

(A-F) The circuit output, measured as calcium levels (middle), in response to various input gradients (Ligand, top). Also shown are the simulated levels of inhibition and receptor activation (bottom). **(A)** In response to periodic on-steps of the ligand (5 s on and 60 s off), the output activity gradually weakens until it reaches a fixed response. The same qualitative response to a similar

stimulation pattern was observed in the AWA neuron (Larsch *et al*, 2015). **(B)** When extending the on-step period and shortening the off-step period (30 s on and 30 s off), the output activity reaches a fixed response after the first step. **(C)** Further increasing the frequency of the stimulus pattern (15 s on and 15 s off) leads to periodic activity skipping, where a calcium pulse is observed in response to every other on-step of the ligand. Skipping occurs because the recovery time from the inhibition (I, equations 1-4) during the short off period (between two consecutives on steps) is too short. This is an expected outcome of negative feedback loops that was also observed in the AWA neuron (Rahi *et al*, 2017). **(D)** Nearly identical output responses when doubling the concentration at each step, reflecting a typical fold-change response. **(E)** In response to a linear gradient, the model outcome is a series of pulses whose frequency and amplitude decay over time. **(F)** In response to an exponential gradient, neural activity increases with the gradient's first derivative. Calcium dynamics in (E) and (F) is also in agreement with experimental observations(Itskovits *et al*, 2018). All ligand patterns start at time 0 and from a baseline concentration of 1.15μM.

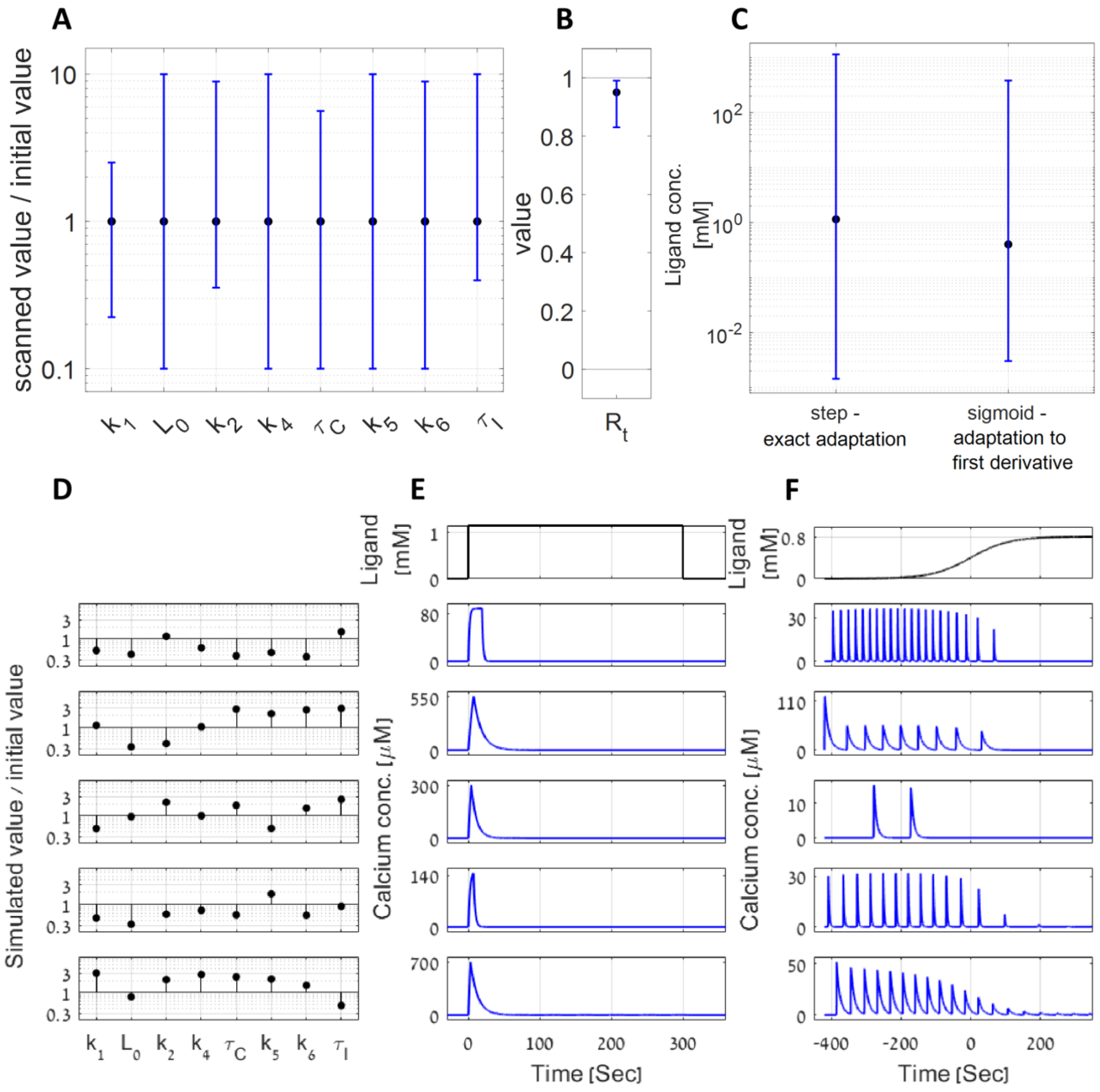

Appendix Figure S2. **The model is insensitive to the exact values of the parameters, and it performs robustly for a wide range of input concentrations.**

(A) Varying each of the model parameters by ~100-fold (10-fold higher and 10-fold lower than the chosen parameters) largely did not affect the model's outcome. In each simulation, we varied only one of the parameters, while fixing all the other parameters, and analyzed if the system fulfilled two main features: (1) exact adaptation and (2) adaptation of the pulsatile response to the first derivative of the stimulus (see methods). As the various parameters differ in their units and scales, their ranges are presented as a multiplication of their initial value. **(B)** $R_t$values (Fraction of active receptors, above which a signaling cascade initiates) are in the $[0,1]$ interval and thus shown

separately. **(C)** The range of ligand concentrations (almost 6 orders of magnitude) in which the two main features of the model were maintained. In all panels, the black dots represent the values used for the simulations in this work (presented in table 1) and error bars denote the range of values for which the system fulfilled the above-mentioned features. **(D-F)** Simulation outputs when randomly varying the different parameters by up to ~10-fold (3.2-fold higher and 3.2-fold lower than the chosen parameters). (D) Shown are five examples for varying the different parameters and the corresponding outputs: (E) for a step function, and (F) for a sigmoidal gradient function. The overall qualitative shape of the responses is similar, though the fine details (*e.g.*, number of pulses and their rise/decay times) may differ.

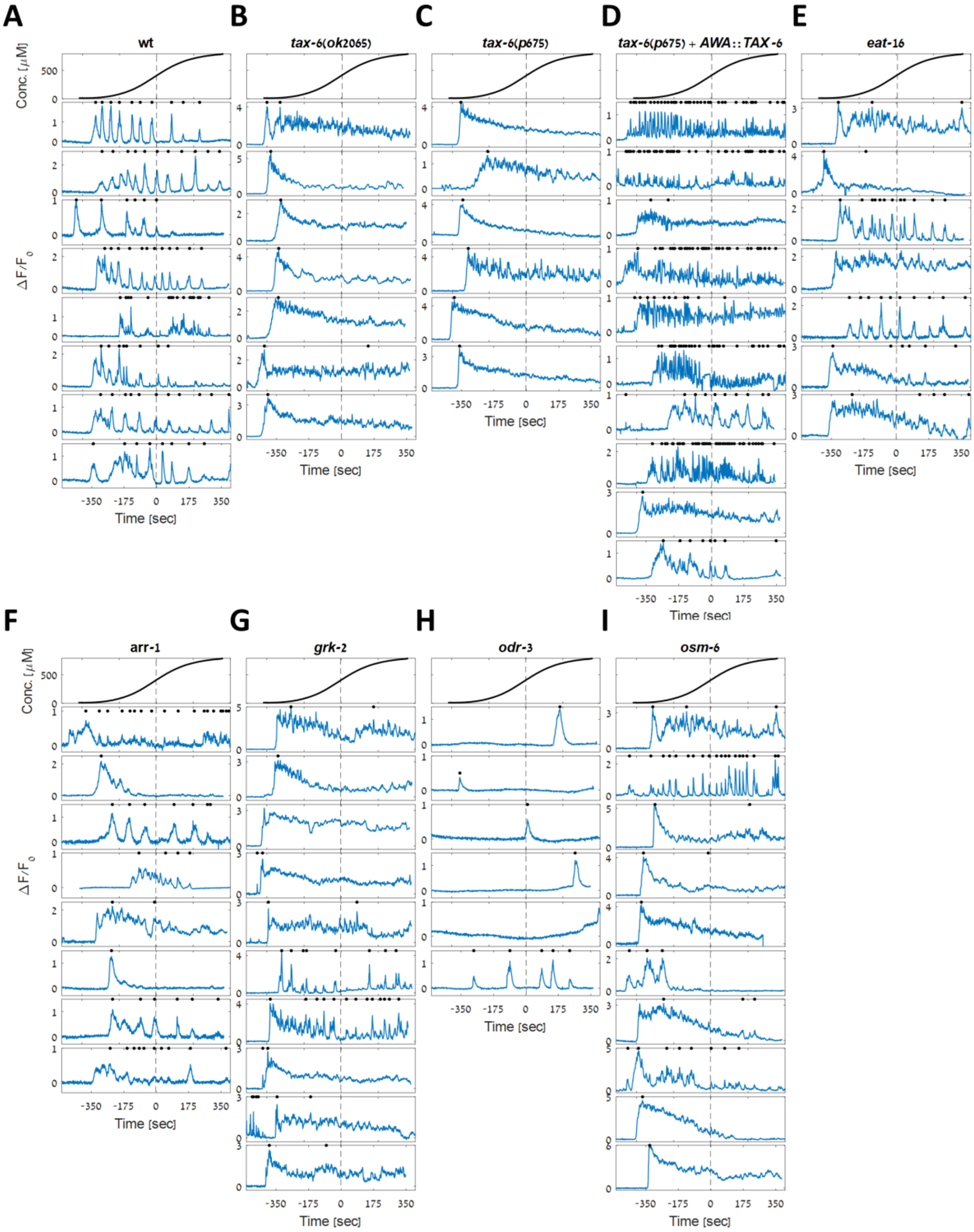
A
wt
B
tax-6(ok2065)
C
tax-6(p675)
D
tax-6(p675) + AWA::TAX-6
E
eat-16
F
arr-1
G
grk-2
H
odr-3
I
osm-6
Conc. [μM]
ΔF/F0
Time [sec]

Appendix Figure S3. **Activity traces of the different mutants in response to sigmoidal gradients.**

(A) WT; **(B)** *tax-6(ok2065)*; **(C)** *tax-6(p675)*; **(D)** *tax-6(p675)+AWA::TAX-6*; **(E)** *eat-16*; **(F)** *arr-1;* **(G)** *grk-2;* **(H)** *odr-3;* **(I)** *osm-6;* **.** Activity of *tax-6* mutant animals reaches a higher amplitude and lacks exact adaptation properties. Note the high variability between and within the various strains. Black dots denote the location of the pulses, as identified by our algorithm (see Methods), and which were used in the statistical analysis of **Figure 3D,E**.

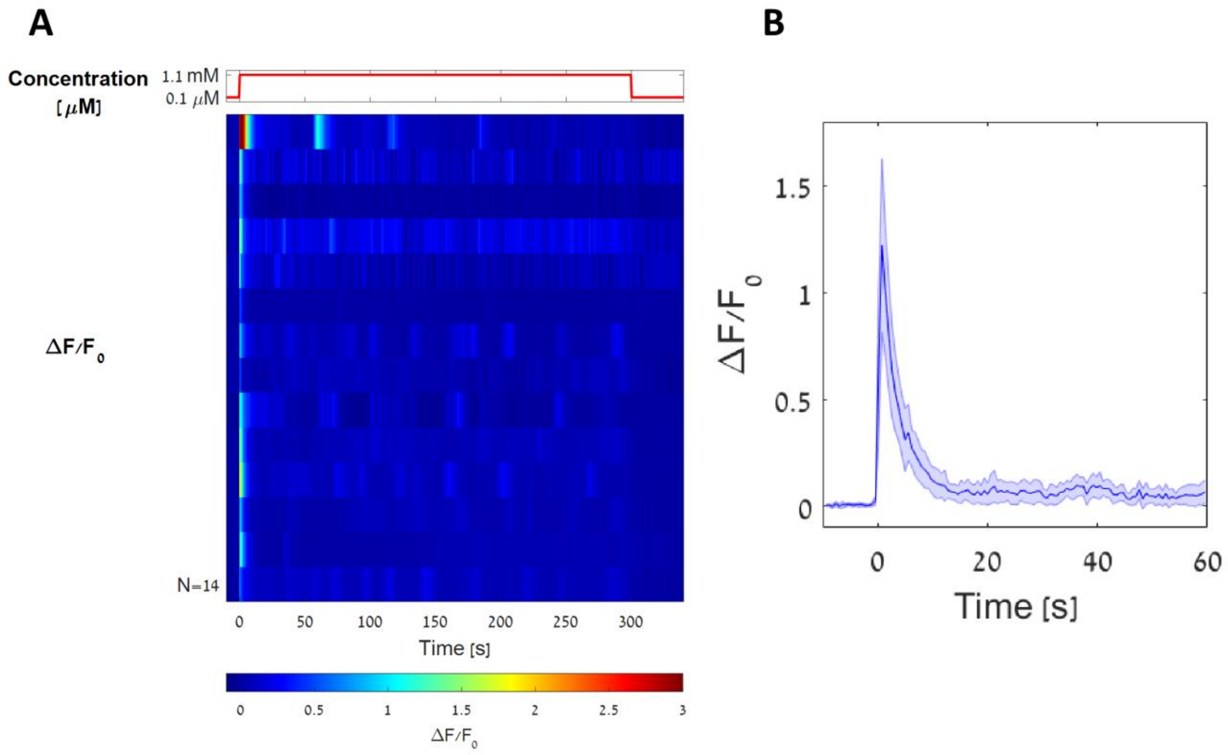

Appendix Figure S4. **Calcium levels in *eat-16* mutant animals show exact adaptation following an on-step stimulus.**

(A) Neural responses of *eat-16* mutants ($N = 14$ worms) to a five-minutes step increase of diacetyl (shown on top). While amplitude response in some neurons was low, all animals showed robust increased levels following the on step. These calcium levels gradually decreased until reaching baseline levels despite the fact that the stimulus remained on. This is in contrast to *tax-6* mutants that fail to return to their baseline activity (as seen in **Figure 4**). **(B)** Median neural activity of *eat-16* worms in response to the step. Shaded area marks mean absolute deviation.

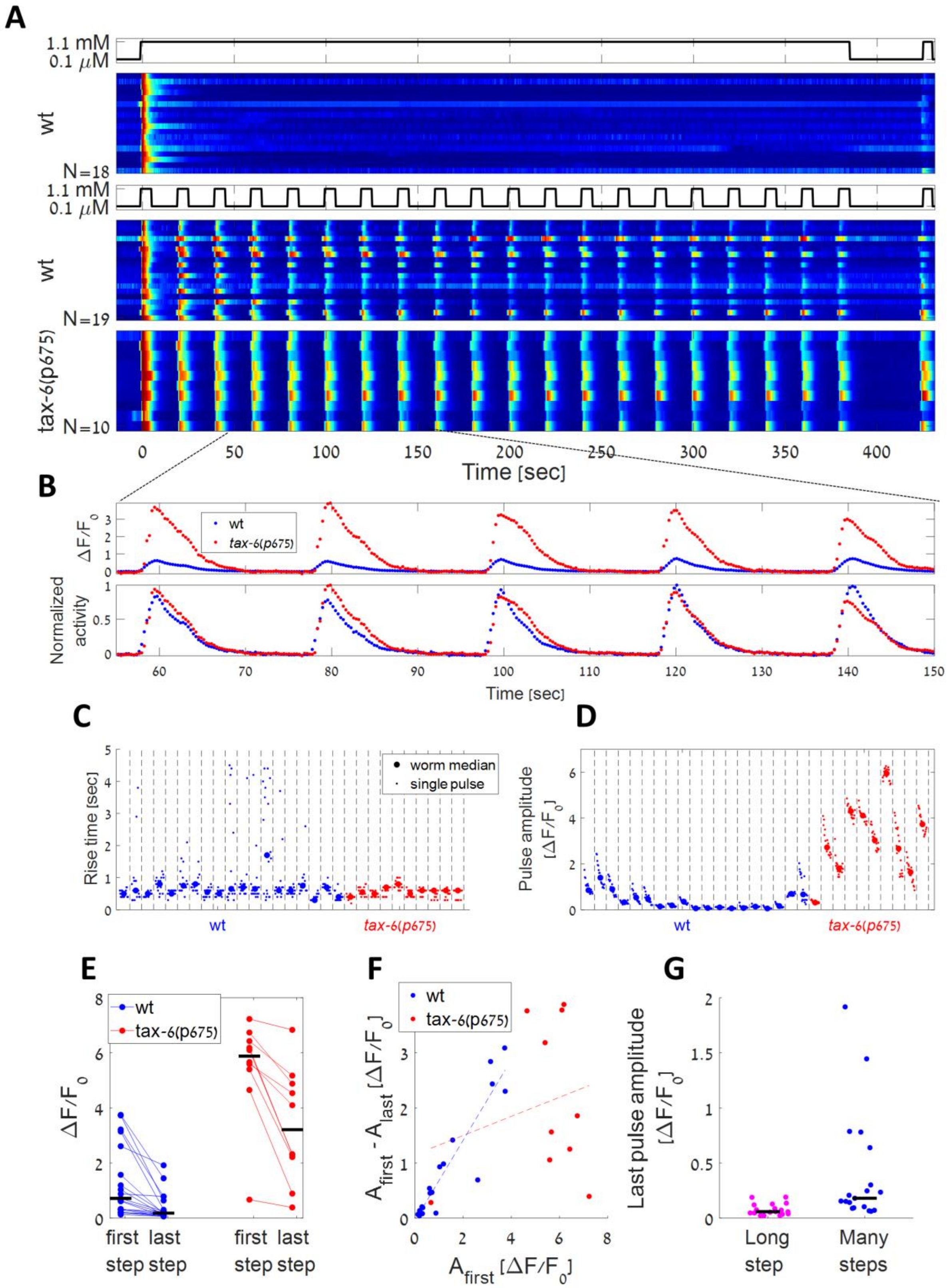
A
1.1 mM
0.1 μM
wt
N=18
1.1 mM
0.1 μM
wt
N=19
tax-6(p675)
N=10
0
50
100
150
200
250
300
350
400
Time [sec]
B
ΔF/F0
wt
tax-6(p675)
Normalized activity
Time [sec]
C
Rise time [sec]
worm median
single pulse
wt
tax-6(p675)
D
Pulse amplitude [ΔF/F0]
wt
tax-6(p675)
E
ΔF/F0
wt
tax-6(p675)
first step
last step
first step
last step
F
wt
tax-6(p675)
A_first - A_last [ΔF/F0]
A_first [ΔF/F0]
G
Last pulse amplitude [ΔF/F0]
Long step
Many steps

Appendix Figure S5. In *tax-6* mutants, the response amplitude is higher than in wt animals, while the response time remains the same.

(A) Responses to steps of diacetyl in individual worms. Worms were subjected to either one long step (top panel, $N = 18$ wt worms) or twenty short 5-seconds long steps (middle and bottom panels, $N = 19, 10$ wt and *tax-6(p675)* worms respectively), followed by a 40-seconds off step and a final 5-seconds on step. Each trace is normalized to its maximal level. **(B)** While the pulse amplitude of *tax-6(p675)* mutants is higher than that of wt worms, the time to reach the peak is similar. A zoomed-in view of representative responses of wt and *tax-6(p675)* worms. Top, absolute values of the response. Bottom, normalized values (by its maximal levels). **(C-D)**. Quantification of the response time, defined as the time to reach 80% of the maximal amplitude (C) and the peak pulse amplitude (D). While the median response time is not significantly different between *tax-6(p675)* mutants and wt worms, the median amplitude of *tax-6(p675)* mutants is significantly higher (Wilcoxon rank-sum test, $p = 9 \cdot 10^{-5}, N = 19, 10$ for wt and *tax-6(p675)* worms respectively). **(E)** Comparison between the amplitudes of wt worms and *tax-6(p675)* mutants in the first and last steps of the short interval steps protocol (shown in (A), middle and bottom panels). In both wt and *tax-6(p675)* worms, the response amplitude to the last step was lower (signed-rank test, $p = 1.3 \cdot 10^{-4}$ and 0.002 respectively), with *tax-6(p675)* mutants having a significantly higher amplitude in both the first and last steps (Wilcoxon rank-sum test, $p = 1.1 \cdot 10^{-4}$ and $6 \cdot 10^{-5}$ respectively). Black bars mark the median. **(F)** The difference between the amplitudes of the first and last steps is plotted as a function of the first step's amplitude. While wt worms show a larger amplitude difference when the response to the first step is stronger ($r = 0.93,\ p < 10^{-8}$), *tax-6(p675)* worms do not ($r = 0.22,\ p = 0.53$). This suggests a link between calcium influx and habituation in wt worms, but not in *tax-6(p675)* mutant worms. **(G)** Comparison between the neural responses of wt worms in the last step of the long-exposure protocol and the multiple-steps protocol. Mean calcium levels are higher during the multiple-steps protocol (as each step generates a new calcium wave response), and this neural activity is also stronger in the last step following the multiple-steps protocol (Wilcoxon rank-sum test, median of $p < 2 \cdot 10^{-4}$). This suggests that calcium is not the sole factor leading to habituation and additional inhibitory mechanisms may exist.

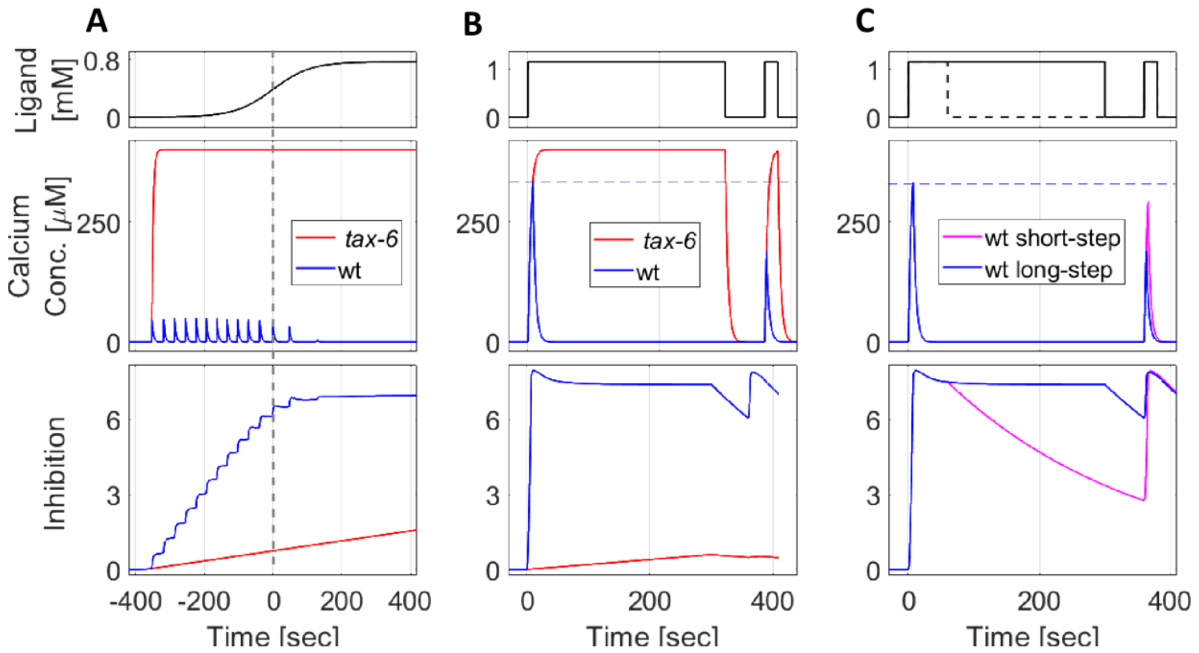

Appendix Figure S6. Eliminating the calcium-dependent inhibition from the model recapitulates key responses observed in *tax-6* mutants, indicating that calcium-independent processes contribute to the inhibitory processes.

Simulations of the model outputs (denoted as calcium concentration, middle) and the inhibition dynamics (bottom) in response to various patterns of stimulus presentation (top). To simulate the responses of *tax-6* mutants, we eliminated calcium-dependent inhibition (by setting k_5 in the model to zero). This is the implicit model (based on **Equations 1-4**) which does not implement the dynamics of the AWA ion channels (Liu *et al*, 2018) as shown in **Figure 6**. The output features are qualitatively similar to the detailed model showing that *tax-6* mutants are unable to achieve exact adaptation and habituate to past stimuli. They also lack pulsatile activity. **(A)** In contrast to the wt dynamics, in response to a sigmoidal gradient (top), simulated *tax-6* worms fail to generate a pulsatile activity and their calcium levels remain at their maximal level for the duration of stimulus presentation, so that exact adaptation was not achieved. **(B)** wt worms exhibit lower responses (increased habituation) following the second on-step. In contrast, *tax-6* mutants did not show exact adaptation, nor a reduced activity in response to the second on-step (no habituation). Indeed, the inhibition remained low compared to wt worms. **(C)** Response dynamics of wt worms following a first long (continues line) or short (dashed line) on-step. A second on-step following a short on-step elicits calcium levels that match the response to the first step. This is due to the long off-step where inhibition is decreasing, thus allowing recovery (purple, note it partially overlaps with the blue and hence not visible). This contrasts with the attenuated response following a long on-step that results in a short recovery time (and hence inhibition remains high,

blue). Notably, even though in both protocols, calcium concentrations returned to baseline levels, the longer the recovery time period, the higher the response activity to a second step (in agreement with experimental results, **Figure 4A**). This suggests that calcium-independent processes contribute to the inhibitory processes in the neuron.

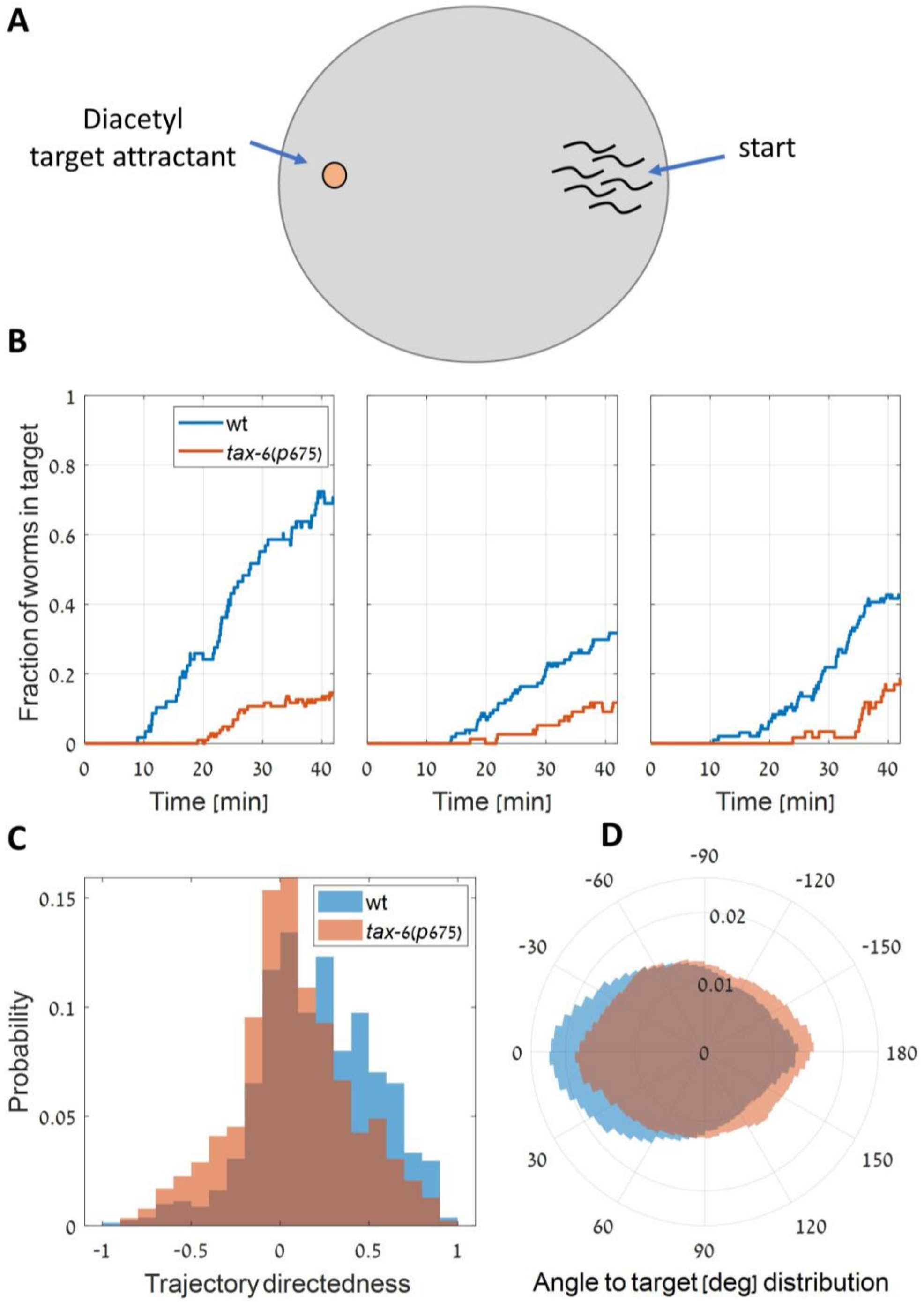

Appendix Figure S7. ***tax-6* mutants are impaired in chemotaxis.**

(A) A layout of a chemotaxis assay. About 100-200 worms are loaded to the starting point and allowed to chemotax towards the target attractant (diacetyl, see methods). **(B)** Worms'

accumulation in the target chemoattractant region of three independent experimental repeats of both wt and *tax-6* mutants. **(C)** Probability distribution of trajectories' directedness(Itskovits *et al*, 2018) for all three experiments, ranging from -1 (trajectory in opposite direction) to +1 (trajectory straight towards the target). Distribution of *tax-6* animals is centered around zero, while the wt distribution is skewed towards positive values. **(D)** Probability distribution of movement angles relative to the target region. An angle was calculated for each frame and concatenated for all trajectories and experiments. While wt distribution is highly biased towards small angles (more directed towards the target), *tax-6* distribution is more uniformly distributed, an expected result given their mostly circular undirected trajectories (**Movies EV2-3**).

Appendix Table S1. The parameters used in the model and their values

Equation	Description	Parameter	Value [units]
1	Activation facilitation by ligand coefficient	k_1	25
1	Scales the dynamic range of detectable input concentration - determined from(Larsch *et al*, 2015) by the minimal concentration detected by worms.	L_0	$1\ [\mu M]$
1	Activation inhibition by modification coefficient	k_2	10
2	Self-enhancing component time constant	k_3	$1\ \left[\frac{1}{ms}\right]$
2	Threshold of required Receptor activation to elicit a pulse	R_t	0.95
3	Calcium influx coefficient	k_4	$1\cdot 10^{-7}\ \left[\frac{M}{ms}\right]$
3	Calcium removal time constant, determined from(Itskovits *et al*, 2018).	τ_c	$4\cdot 10^{3}\ [ms]$
3	Steady state calcium level, determined from(Liu *et al*, 2018).	C_0	$0.1\ [\mu M]$
4	Calcium mediated inhibition coefficient	k_5	$5\ \left[\frac{1}{M\cdot ms}\right]$
4	Receptor activity mediated inhibition constant	k_6	$2\cdot 10^{-6}\ \left[\frac{1}{ms}\right]$
4	Inhibition removal time constant	τ_I	$3\cdot 10^{5}\ [ms]$
2.1	Current influx constant for integrated model	$c_{current}$	$35\ [pA]$
3.1	Calcium current to concentration change AWA constant (determined from physical properties of the AWA neuron)	c_{AWA}	$2.6\cdot 10^{-8}\ \left[\frac{M}{ms\cdot pA}\right]$

Appendix Note S1.

Part 1. A detailed description and analysis of the mathematical model

Our model for chemo-sensation in the AWA neurons is composed of a system of time dependent differential equations. The circuit's topology (**Figure 1**) is analogous to the negative feedback loop circuit found in the chemosensory system of *E. coli (Tu et al, 2008)*. We therefore capitalized on the mathematical framework developed in these studies and modified it to adjust to the known GPCR signaling pathway found in the AWA neurons of *C. elegans* worms.

Our model consists of five variables. The "Ligand" ($\boldsymbol{L}$) denotes the input stimulus (*e.g.,* diacetyl). The Receptor Activation term ($\boldsymbol{R_a}$) represents the fraction of active ligand-bound receptors in the cell membrane. $\boldsymbol{R_a}$ is facilitated by ligand binding and is regulated by Inhibition ($\boldsymbol{I}$) which represents the effect of inhibiting factors. Overall, $\boldsymbol{R_a}$ is modeled as a sigmoidal function of the difference between the Ligand and Inhibition terms:

$$(1) \qquad \boldsymbol{R_a} = \left(1 + exp\left(-k_1 log\left(\frac{\boldsymbol{L}}{L_0}\right) + k_2 \boldsymbol{I}\right)\right)^{-1} \qquad , \boldsymbol{R_a} \,\epsilon\, (0,1)$$

Where k_1, L_0, k_2 are positive constants. In this functional form, $\boldsymbol{I}$ serves as the system's "memory" of past ligand concentrations. The logarithmic-scale coding of the ligand and the linear regulation of the negative feedback ("Inhibition" motif) are concepts borrowed from chemo-sensation models in *E. coli(Shimizu et al, 2010; Yi et al, 2000; Tu et al, 2008)*.

$\boldsymbol{R_a}$ facilitates a self-enhancing component ($\boldsymbol{S}$), which represents the VGCC and TRPV channels combined. Their dynamics can be described by:

$$(2) \qquad \frac{d\boldsymbol{S}}{dt} = k_3(\boldsymbol{R_a} - R_t)\boldsymbol{S} \qquad , \boldsymbol{S} \,\epsilon\, (0,1)$$

Thus, when $\boldsymbol{R_a}$ crosses a critical activation threshold R_t $(0 < R_t < 1)$, $\boldsymbol{S}$ will grow/fall exponentially. $\boldsymbol{S}$ is approximated to form a self-enhancing motif where a small calcium influx generated by the $\boldsymbol{R_a}$-mediated opening of TRPV channels initiates further opening and self-amplification of VGCCs, through which most of the calcium enters the cell(Larsch *et al*, 2015; Liu *et al*, 2018).

$\boldsymbol{S}$ values are limited by code to the interval $\boldsymbol{S} \,\epsilon\, (0,1]$, to simplify a more realistic sigmoidal behavior. Its lower limit is a small (<<1) positive number, and its exact limit does not affect the model performance (not shown). Thus, $\boldsymbol{S}$ produces a "two-state" switch where the neuron is either

active or inactive. For this, the time constant k_3^{-1}, needs to be smaller than the other time constants in the system, though its specific values do not significantly affect the model performance.

In equation 3, $\boldsymbol{S}$ facilitates calcium accumulation in the cell ($\boldsymbol{C}$):

$$(3) \qquad \frac{d\boldsymbol{C}}{dt} = k_4\boldsymbol{S} - \frac{1}{\tau_c}(\boldsymbol{C} - C_0)$$

Calcium enters through open channels ($\boldsymbol{S}$) and is removed by calcium pumps. The removal time constant, τ_c was approximated experimentally to be in the order of several seconds (Itskovits *et al*, 2018). C_0 is the calcium level at rest so that $\boldsymbol{C} \geq C_0$. The calcium in the cell facilitates the Inhibition term, according to equation 4:

$$(4) \qquad \frac{d\boldsymbol{I}}{dt} = k_5(\boldsymbol{C} - C_0)\boldsymbol{R_a} + k_6\boldsymbol{R_a} - \frac{1}{\tau_I}(1 - \boldsymbol{R_a})\boldsymbol{I}$$

Where k_5, k_6 and τ_I are positive constants. Two components increase inhibition levels: the first depends on calcium (with the constant k_5) and the second is calcium independent (denoted with the constant k_6). The calcium-dependent term is consistent with homologous adaptation by G protein-coupled receptor kinases (GRKs) that phosphorylate only active receptors (which is why the term is multiplied by $\boldsymbol{R_a}$). The second term depends on receptor activity only and is consistent with phosphorylation driven by second messenger-stimulated kinases(Lefkowitz, 1998).

These two terms fulfill two different features in the system. To achieve robust exact adaptation, the circuit's output needs to directly inhibit the receptor(Yi *et al*, 2000). Therefore, as calcium directly correlates with the synaptic output(Katz & Miledi, 1970; Llinás *et al*, 1976), its regulation of receptor inhibition underlies robust exact adaptation. However, as calcium levels are transient, adaptation should also depend on the degree of receptor activation to promote inhibition for as long as the stimulus is present. Our experimental results support this logic as we find that calcium levels correlate with adaptation magnitude, and this adaptation persists even after calcium removal (**Figure 4**). These results suggest two separate adaptation mechanisms: one that is calcium dependent and the other which relies on the receptor's activity.

The last term in equation 4 denotes inhibition removal which depends on inactive receptors only (thus multiplying $\boldsymbol{I}$ by $(1 - \boldsymbol{R_a})$), similar to methylation dynamics in *E. coli(Shimizu et al, 2010)*. To close the negative feedback loop, this inhibition ($\boldsymbol{I}$) regulates receptor activation according to equation 1.

Part 2. Mathematical analysis of the model's steady state shows how exact adaptation is achieved

The mathematical formulation of exact adaptation requires that following a step increase in the attractant, the steady state value of the circuit output must return to its basal pre-stimulation levels. To examine this transition, we set $\boldsymbol{L} \rightarrow L_1$and require $\frac{d\boldsymbol{S}}{dt} = \frac{d\boldsymbol{C}}{dt} = \frac{d\boldsymbol{I}}{dt} = 0$. From equation 2, a stable equilibrium can only be reached by $\boldsymbol{S} = 1$ or $\boldsymbol{S} \rightarrow 0$. A steady state in which $\boldsymbol{S} = 1$ represents a non-pulsatile response, in which calcium stays at its maximum, and can be obtained by choices of parameters where calcium-mediated inhibition is low (as in *tax-6* mutants, **Appendix Figure S6**). A steady state in which $S \rightarrow 0$ may lead to exact adaptation as follows: by plugging $S = 0$ in equation 3, we get a steady-state calcium level that does not depend on the input: $C_{SS} = C_0$. Furthermore, plugging these results in equation 4, yields:

$$(5) \qquad \boldsymbol{I} = k_6 \tau_I \frac{R_{SS}}{1 - R_{ss}}$$

If we plug $\boldsymbol{I}$ in equation 1, we get a closed form for the receptor activation during steady state:

$$(6) \qquad R_{SS} = \left(1 + exp\left(-k_1 log\left(\frac{L_1}{L_0}\right) + k_2 k_6 \tau_I \frac{R_{SS}}{1-R_{SS}}\right)\right)^{-1}$$

This relation can be solved numerically, and by plugging this solution back to equation 5, we get the steady state inhibition level, I_{SS}. We can see that steady-state activation and inhibition do depend on the input level L_1, thus serving as a 'memory' of the input, even after output returns to basal levels.

Considering dynamics, when responding to a step-function input, receptor activation will rise above R_t, elicit an output pulse that will, in turn, increase inhibition. Inhibition will eventually decrease receptor activation to terminate the pulse. In the regime where $R_{ss} > R_t$, receptor activation will rise and cross R_t before reaching steady state, and thus elicit another pulse. Therefore, a strict requirement for exact adaptation in this simple model is $R_{ss} < R_t$.

Part 3. How smooth gradients of input are encoded as a pulsatile response that adapts to the gradient's first derivative?

Our model translates smooth gradients of the input into a pulsatile response output: Increased ligand levels lead to receptor activation (eq. 1). At this stage, since there is still no output, inhibition is relatively low (eq. 4), and for simplification, we assume its effect on receptor

activation to be negligible. When receptor activation reaches its threshold level, R_t, a pulse of calcium is generated (eqs. 2 and 3). High levels of calcium rapidly increase inhibition (eq. 4), though at this time scale, ligand concentrations remain relatively constant. The rapid increase in inhibition causes a sharp decline in receptor activation levels (eq. 1), and when decreased below R_t, the pulse terminates (eq. 2,3). Calcium is being removed and hence calcium-mediated inhibition slowly decreases (eq. 4), while ligand concentrations increase to elicit another pulse (as can be seen in **Figure 2B**).

To explain how the above pulsatile response adapts to the first derivative of the input gradient, we analyze the model with the following assumptions: (1) a calcium pulse is an immediate event, in which ligand levels remain constant, and the inhibition rises to a new higher level; (2) the change in inhibition during a pulse always results in a constant decrease of receptor activation; (3) between pulses, inhibition remains constant, so that only the ligand can change receptor activation.

We assume a first pulse occurs when activation reaches R_t at a ligand level L_1 and inhibition level I_1. This pulse increases inhibition to a new level, I_2, so that receptor activation falls to a new level $R_0 < R_t$:

$$(7) \qquad R_0 = \left(1 + \exp\left(-k_1 log\left(\frac{L_1}{L_0}\right) + k_2 I_2\right)\right)^{-1}$$

To elicit an additional pulse, the ligand concentration has to rise to a new level, L_2, so that receptor activation reaches R_t once again. Inhibition is assumed to stay constant, thus when a second pulse is produced:

$$(8) \qquad R_t = \left(1 + \exp\left(-k_1 log\left(\frac{L_2}{L_0}\right) + k_2 I_2\right)\right)^{-1}$$

Isolating I_2 in equation 7, and placing in equation 8 yields:

$$(9) \qquad L_2 = A \cdot L_1$$

Where $A = \left(\frac{R_t(1-R_0)}{R_0(1-R_t)}\right)^{\frac{ln10}{k_1}}$ is greater than 1 since $R_t > R_0$ and $k_1 > 0$. Thus, to produce the consecutive pulse, ligand levels need to increase by a factor A. For a linear increase in ligand levels, $\boldsymbol{L} = \alpha t$, this means that the n'th pulse time, and the time between consecutive pulses will be:

$$(10) \qquad t_n = \frac{L_1}{\alpha} A^{n-1} \quad ; \quad \Delta t_n = \frac{L_1}{\alpha} A^{n-1}(A-1)$$

Thus, the time interval between consecutive pulses grows with the number of pulses. The same consideration will also dictate less pulses in the second half of a sigmoidal gradient (decreasing first derivatives of the gradient), effectively exhibiting adaptation to the first derivative.

Part 4. Integrating the model with known dynamics of voltage gated ion channels in AWA.

To accurately account for the main voltage-gated ion channels in the AWA neuron, we integrated our model with a model developed in the Bargmann lab based on intracellular recordings of the AWA neuron(Liu *et al*, 2018). This model takes electrical current influx as an input and simulates the dynamics of ion channels and membrane potential in the cell. To integrate this model into our simulations, the electrical current influx was considered to be proportional to our self-enhancing motif:

(2.1) $$\boldsymbol{I} = c_{current}\boldsymbol{S}$$

Where $\boldsymbol{I}$ is the current influx, $\boldsymbol{S}$ is the self-enhancing motif, and $c_{current}$ is the proportion constant which determines the connectivity between the models. Thus, the current influx is provided as the input to the model:

(2.2) $$[\boldsymbol{V}, \boldsymbol{I_{Ca}}] = f(\boldsymbol{I})$$

Where f is the model, $\boldsymbol{V}$ is the membrane potential and $\boldsymbol{I_{Ca}}$ is the calcium influx entering the cell. Calcium influx is therefore plugged into equation 3, instead of S:

(3.1) $$\frac{d\boldsymbol{C}}{dt} = c_{AWA}\boldsymbol{I_{Ca}} - \frac{1}{\tau_C}(\boldsymbol{C} - C_0)$$

Where c_{AWA} is a constant that changes the electrical current units to concentration changes while accounting for the volume of the AWA neuron. To determine this constant, we approximated the cell volume based on our microscopy images to be: $V_{AWA} = 9 \cdot 4 \cdot 4\ \mu m^3 \sim 2 \cdot 10^{-13}\ L$, and used the following transformation:

$$\frac{d\boldsymbol{C}}{dt} = \frac{1}{2 \cdot q_e \cdot N_A \cdot V_{AWA}}\boldsymbol{I_{Ca}}$$

Where q_e is the elementary charge, N_A is the Avogadro number, and $\frac{1}{2}$ accounts for the charge of a calcium ion.

Overall, in the detailed integrated model, equations 2.1 and 2.2 replace equation 2 (in the original model), and equation 3.1 replaces equation 3 (in the original model).

Part 5. Comparing our model with the adaptive-threshold mechanism.

Levy and Bergmann (Levy & Bargmann, 2020) proposed the adaptive threshold mechanism for the timing at which the AWC neurons in *C. elegans* are activated in response to a chemical cue. Here we show that this model can be viewed as mathematically analogous to our model (with minor assumptions) which also provides the initiation timing of the pulses in response to stimulus gradients.

According to the adaptive threshold mechanism, the threshold is not fixed, but rather dynamically changes depending on the history of the perceived stimulus. To show how this can be equivalent to our model, we consider a smooth slowly-increasing gradient, for which we wish to calculate the concentration threshold required for the stimulus to cross in order to initiate a pulse. In our model, the condition for pulse initiation is that the fraction of active receptors crosses a threshold (eq. 2):

$$\boldsymbol{R_a} > R_t$$

$\boldsymbol{R_a}$ is a sigmoidal function of the difference between the Ligand term and the inhibition term (eq. 1):

$$\boldsymbol{R_a} = \left(1 + exp(-\boldsymbol{x})\right)^{-1} \quad , \quad \boldsymbol{x} \equiv k_1 \log\left(\frac{\boldsymbol{L}}{L_0}\right) - k_2 \boldsymbol{I}$$

For simplicity, we will use Taylor approximation to expand the sigmoid function for a small $\boldsymbol{x}$, such that the two terms of the right-hand equation are of the same magnitude, roughly canceling one another:

$$\left(1 + exp(-\boldsymbol{x})\right)^{-1} \approx \frac{1}{2} + \frac{\boldsymbol{x}}{4} \quad , \quad \boldsymbol{x} \in [0,1]$$

For smooth increasing gradients, before pulse initiation, $\boldsymbol{R_a}$ rises monotonically. Moreover, $\boldsymbol{R_a}$ will not reach saturation (i.e., $\boldsymbol{R_a} = 1$), because a pulse will be initiated once $\boldsymbol{R_a}$ crosses R_t ($R_t < 1$). Thus, we continue to consider the linear regime of the function. With this simplification, we get:

$$(i) \qquad \boldsymbol{R_a} \approx \frac{1}{4}\left(k_1 \log\left(\frac{\boldsymbol{L}}{L_0}\right) - k_2 \boldsymbol{I}\right) + \frac{1}{2}$$

Next, when considering the inhibition dynamics (eq. 4), we make the following assumptions: (1) before the initiation of the first pulse, or between consecutive pulses, calcium levels are close to baseline levels such that they do not contribute to the inhibition. (2) The inhibition removal term, which is a function of the non-active receptors, is negligible since its time constant is an order of magnitude larger (300 sec) than the inter-pulse time (~10 sec). This

assumption should hold as long as the ligand concentration changes fast enough to promote several pulses within the ~300 seconds time frame. With these simplifications, we can neglect the first and the third terms in equation 4 and then substitute equation (i):

$$(ii) \qquad \frac{d\boldsymbol{I}}{dt} = \frac{1}{4}k_1 k_6 \log\left(\frac{\boldsymbol{L}}{L_0}\right) - \frac{1}{4}k_2 k_6 \boldsymbol{I} + \frac{1}{2}k_6$$

The solution for this first-order, linear, ODE is:

$$(iii) \qquad \boldsymbol{I}(t) = \left(I_0 - \frac{2}{k_2}\right)e^{-\frac{1}{4}k_6 k_2 t} + \frac{2}{k_2} + \frac{1}{4}k_6 k_1 \int_0^t log\left(\frac{\boldsymbol{L}(t')}{L_0}\right)e^{-\frac{1}{4}k_6 k_2 (t-t')}dt'$$

Where $I_0 = \boldsymbol{I}(t=0)$ is the initial inhibition level. Plugging equation (iii) into equation (i), the condition for pulse initiation, $\boldsymbol{R_a} > R_t$, becomes:

$$\frac{1}{4}k_1 \log\left(\frac{\boldsymbol{L}}{L_0}\right) - \frac{1}{4}k_2\left(I_0 - \frac{2}{k_2}\right)e^{-\frac{1}{4}k_6 k_2 t} - \frac{1}{16}k_2 k_6 k_1 \int_0^t log\left(\frac{\boldsymbol{L}(t')}{L_0}\right)e^{-\frac{1}{4}k_6 k_2 (t-t')}dt' > R_t$$

This can be reorganized to:

$$(iv) \qquad \log\left(\frac{\boldsymbol{L}}{L_0}\right) - \frac{4R_t}{k_1} > \frac{k_2}{k_1}\left(I_0 - \frac{2}{k_2}\right)e^{-\frac{1}{4}k_6 k_2 t} + \frac{1}{4}k_2 k_6 \int_0^t log\left(\frac{\boldsymbol{L}(t')}{L_0}\right)e^{-\frac{1}{4}k_6 k_2 (t-t')}dt'$$

Thus, for initiating a pulse, the logarithm of the ligand concentration, shifted by a constant (left hand side of equation iv), needs to cross an adaptive threshold term (right hand side of the equation iv). At time zero, the threshold is $\frac{k_2}{k_1}(I_0 - \frac{\mathbf{2}}{k_2})$. This initial threshold, however, is forgotten exponentially fast, and is replaced by a new adaptive threshold which integrates the history of ligand concentrations with an exponential kernel.

References

Itskovits E, Ruach R, Kazakov A & Zaslaver A (2018) Concerted pulsatile and graded neural dynamics enables efficient chemotaxis in C. elegans. *Nature Communications* 9 doi:10.1038/s41467-018-05151-2

Katz B & Miledi R (1970) Further study of the role of calcium in synaptic transmission. *The Journal of Physiology* 207: 789–801 doi:10.1113/jphysiol.1970.sp009095

Larsch J, Flavell SW, Liu Q, Gordus A, Albrecht DR & Bargmann CI (2015) A Circuit for Gradient Climbing in C. elegans Chemotaxis. *Cell Rep* 12: 1748–1760

Lefkowitz RJ (1998) G protein-coupled receptors. III. New roles for receptor kinases and beta-arrestins in receptor signaling and desensitization. *J Biol Chem* 273: 18677–18680

Levy S & Bargmann CI (2020) An Adaptive-Threshold Mechanism for Odor Sensation and Animal Navigation. *Neuron* 105: 534–548.e13

Liu Q, Kidd PB, Dobosiewicz M & Bargmann CI (2018) C. elegans AWA Olfactory Neurons Fire Calcium-Mediated All-or-None Action Potentials. *Cell* 175: 57–70.e17

Llinás R, Steinberg IZ & Walton K (1976) Presynaptic calcium currents and their relation to synaptic transmission: voltage clamp study in squid giant synapse and theoretical model for the calcium gate. *Proc Natl Acad Sci U S A* 73: 2918–2922

Rahi SJ, Larsch J, Pecani K, Katsov AY, Mansouri N, Tsaneva-Atanasova K, Sontag ED & Cross FR (2017) Oscillatory stimuli differentiate adapting circuit topologies. *Nat Methods* 14: 1010–1016

Shimizu TS, Tu Y & Berg HC (2010) A modular gradient-sensing network for chemotaxis in Escherichia coli revealed by responses to time-varying stimuli. *Mol Syst Biol* 6: 382

Tu Y, Shimizu TS & Berg HC (2008) Modeling the chemotactic response of Escherichia coli to time-varying stimuli. *Proc Natl Acad Sci U S A* 105: 14855–14860

Yi TM, Huang Y, Simon MI & Doyle J (2000) Robust perfect adaptation in bacterial chemotaxis through integral feedback control. *Proc Natl Acad Sci U S A* 97: 4649–4653
