# Supplementary material for: A negative feedback loop in the GPCR pathway underlies efficient coding of external stimuli
Source: Mol Syst Biol. 2022 Sep 15;18(9):e10514. doi: 10.15252/msb.202110514 (PMC9476886; doi:10.15252/msb.202110514)
Supplement: Supplementary file 11 [file MSB-18-e10514-s002.pdf]

molecular systems biology

A negative feedback loop in the GPCR pathway underlies efficient coding of external stimuli

Rotem Ruach, Shai Yellinek, Eyal Itzkovitz, Noa Deshe, Yifat Eliezer, Eduard Bokman, and Alon Zaslaver
DOI: 10.15252/msb.202110514

Corresponding author(s): Alon Zaslaver (alonzas@mail.huji.ac.il)

Review Timeline:

Submission Date:	18th Jun 21
Editorial Decision:	21st Jul 21
Revision Received:	18th Jun 22
Editorial Decision:	21st Jul 22
Revision Received:	18th Aug 22
Accepted:	22nd Aug 22

Editor: Maria Polychronidou

Transaction Report:

(Note: With the exception of the correction of typographical or spelling errors that could be a source of ambiguity, letters and reports are not edited. Depending on transfer agreements, referee reports obtained elsewhere may or may not be included in this compilation. Referee reports are anonymous unless the Referee chooses to sign their reports.)

1st Editorial Decision **21st Jul 2021**

Manuscript Number: MSB-2021-10514, A GPCR negative feedback loop underlies efficient coding of external stimuli

Thank you again for submitting your work to Molecular Systems Biology. We have now heard back from the three referees who agreed to evaluate your study. Overall, the reviewers acknowledge that the study is a relevant contribution to the field. They raise however a series of concerns, which we would ask you to address in a major revision.

I think that the reviewers' recommendations are clear and it is therefore not required to repeat the points listed below. Most issues refer to the need to preform additional analyses, including experimental ones, in order to better support the main conclusions. All issues raised by the reviewers need to be satisfactorily addressed. Please contact me in case you would like to discuss in further detail any of the issues raised.

On a more editorial level, we would ask you to address the following points:

Reviewer #1:

In a previous study, the authors showed that, during olfactory chemotaxis by C. elegans, AWA olfactory neurons exhibit pulsatile responses when exposed to a continuous increase in the concentration of an attractive odorant diacetyl. In this study, they assumed molecular feedback circuits in AWA neurons and reproduced the pulsatile activity based on mathematical modeling. They further showed that the calcium-dependent protein phosphatase calcineurin TAX-6 plays an important role in this activity. However, although I agree that the model is reasonable and represents a reasonable reproduction of the wild-type pulsatile responses, I still have a number major and minor concerns, which I believe should be addressed prior to the publication of this work. These are listed below.

Major:

(1) In their genetic analyses, the authors used only a single mutant allele for each gene, and no rescue experiment was conducted. It is thus possible that the phenotypes described in the manuscript may be caused by side mutations. In order to clarify this issue, it is necessary to use at least two alleles for each gene or alternatively conduct rescue experiments.

(2) Related: The authors provide no clear evidence of cell autonomy. To the best of my knowledge, the possibility that diacetyl is sensed by neuron(s) other than AWA and the neuron(s) affecting AWA activity has not been disproved.

(3) A further related issue concerns the authors' assumption of a molecular feedback loop consisting of GPCR -> G_alpha -> TRP channel -> VGCC -> calcium entry -> calcineurin -| GPCR or G_alpha. Although this scenario is plausible in terms of molecular and cellular biology, the assumed operation of this mechanism would be dependent on all the participating molecules being localize in very close proximity; that is, at the sensory ending of the neuron. Is this true? Certainly, this assumption would be reasonable for the GPCR, G_alpha, and TRP channels; however, as far as I understand, VGCC is not considered to localizes at the worm's sensory ending. Also, what about TAX-6? Moreover, they monitored calcium dynamics within the cell body, rather than the sensory ending. Thus, I would like the authors to present some experimental evidence showing that VGCC and TAX-6 indeed work in the feedback loop.

(4) The title of the manuscript includes the expression "A GPCR negative feedback loop." However, what is the evidence for the involvement of GPCR? Is it because diacetyl is sensed by the GPCR ODR-10? In my understanding, the arr-1 mutation did not significantly affect the AWA response. Thus, the defective phenotype observed only in (a single allele of) eat-16 is used to support the claim, whereas another component of GPCR signaling, arrestin, is not involved. I do not believe this constitutes sufficient evidence to corroborate the authors' contentions and thus, as discussed above, further supporting evidence is needed.

(5) What is the physiological significance of the dynamic AWA calcium response to a gradual increase in odor stimulus? It is OK that animals sense several minutes (~400 s) of continuous gradual increase in odor concentration. That happens in general. Then, the data presented by the authors indicate that AWA shows 10 times of transient (pulsatile) calcium increases every 30 to 60 s. How does such a series of AWA activations affect the worm's behavior?

Collectively, although the overall model appears elegant, the link between the model components and molecular activity, as well as its physiological significance, have yet to be sufficiently explained and/or supported by evidence.

(6) In the title and early part of the Introduction, said the authors use the term "coding," which, in my understanding, is taken to indicate relaying environmental information to downstream neurons. However, there is no description of these downstream neurons.

(7) p. 5 middle: The authors claim that "The pulsatile activity correlates with the first derivative of the gradient and adapts to the magnitude of the first derivative." I was unable to grasp the meaning of this sentence, particularly from a quantitative perspective, even after having gone through their previous paper (Itskovits et al., 2018). On the basis of the information presented in Figure 2, for the step-like stimulus (panel a), I understand that the response (ΔF/F0) is quite similar to dL/dt. However, for a gradually increasing stimulus, how is ΔF/F0 "correlated" with the first derivative? The shapes of dL/dt and ΔF/F0 appear to differ substantially. During the early phase, dL/dt was very small and close to zero; however, ΔF/F0 was already high (indeed at almost its highest). How does then this pattern change thereafter? Do the authors claim that when dL/dt increases, the peak magnitude decreases to a certain extent and/or the intervals of the peaks become longer? I do not agree with this, given that just a single example is shown. Actually individual responses are shown in Sup. Fig S4, although there are only eight examples, and interpretation of the results could differ owing to lack of quantitative analysis. I was unable to find a quantitative analysis based on actual AWA responses even in the previous 2018 paper. Thus, I would like to conclude that the authors' claim (the quoted sentence at the top) is insufficiently supported by the data presented and would thus request more quantitative analysis of the pulsatile activity characteristics in wild-type animals.

(8) p. 6: In my understanding, the key to this study is the modeling of AWA activity using equations 1 to 4, which were in fact developed for bacterial chemotaxis by a different group. Then, what is the originality of this study? It would appear that the authors simply applied a well-established method to examine C. elegans neuronal activity.

(9) Related: p. 8, "Our model predicts..." The authors claim that certain features of AWA responses are reproduced in their model. However, I was not convinced that the responses of AWA are characterized by such features, owing to the lack of quantitative evidence.

(10) p. 8, "The model implements..." (Sup Fig. S2): I agree that the k5 pathway is essential. However, k6 may not play a prominent role. Moreover, the authors only show model responses to a step-like stimulus. It would be informative to examine the responses of the model to a gradual stimulus in the absence of k5 or k6.

(11) p. 8, "Notably,,," (Sup Fig. S3): Although the authors show the ranges of parameters, they do not describe the outcomes of the models with different parameters. What does this figure purport to show?

(12) I understand that the long-lasting response of AWA neurons in tax-6 mutants can be explained by the lack of a Ca++-dependent feedback pathway. However, does the model explain the large initial response to the step-like stimulus in tax-6 mutants? This does not appear to have been reproduced in the model (Fig. 6b). This should not be taken as a criticism, but would be of interest to know.

Minor:

(1) There are too many typographical errors to point out individually. In my opinion, the submitted manuscript has not been sufficiently well prepared. I am constantly disappointed by authors (not just the authors of this particular manuscript, but those in general) who prematurely submit poorly constructed manuscripts, in the expectation that reviewers will play the role of the grammar-check function of a word processing application.

(2) p. 2-5: In my opinion, the Introduction is too long, in excess of 1,000 words. Moreover, in addition to the length, it contains information that is not strictly related to the Results. I accordingly recommend reducing the word count to less than 700.

Reviewer #2:

C. elegans performs olfactory behaviors using a small number of sensory neurons in a small nervous system. Each sensory neuron is endowed with substantial functionality to allow the animal to sense absolute and relative concentrations of many different ambient chemicals. Much is known about signal transduction in E. coli chemotaxis where all molecules in its biochemical pathway have been identified and dissected. Models for bacterial chemotaxis are rigorous and well-tested.

C. elegans olfaction is beginning to be explored in similarly quantitative ways. This paper represents a substantial contribution in this growing research area. Worm genetics has identified a number of molecules that contribute to signal transduction in the G-protein coupled pathways in their OSNs. One GPCR has been identified (ODR-10), and roles for a number of commonly studied molecules like calcineurin, guanylyl receptor kinases, and arrestin have been assigned using behavioral phenotypes.

With the rise of quantitative physiological methods with single-cell resolution (mostly calcium imaging), it is now possible to pursue integrated dynamical models of the molecular pathways that underlie olfactory processing. GPCR signaling pathways have been intensively and successfully modeled in other systems, notably vertebrate photoreception. It would be amazing to capitalize on the strengths of worm genetics to better understand these pathways in olfactory sensory neurons.

The authors focus on the AWA sensory neuron. AWA senses diacetyl over a large concentration range (7 orders of magnitude). In response to a stepwise increase of diacetyl concentration, AWA exhibits a large increase in calcium levels that perfectly adapts to prestimulus levels. In response to a gradual change in diacetyl concentrations, AWA exhibits pulsatile dynamics.

Rahi et al. argued that the AWA olfactory response exhibits adaptation by negative feedback
based on calcium imaging in response to a series of diacetyl pulses [1]. We know many molecules that work between diacetyl detection by ODR-10 and calcium dynamics. These include TRPV channels [2] and voltage-gated calcium channels [3]. Several molecules associated with G-protein coupled pathways in AWA and other OSNs have been identified. These include kinases and arrestin that typically downregulate GPCRs (but which lead to unusual and interesting phenotypes in C. elegans [4], [5]), TAX-6/calcineurin (which has been suggested to negative regulate sensory signaling in worms [6]), EAT-16 (which negatively regulates activated G-protein pathways [7], and OSM-6 which AWA needs for habituation to repeated diacetyl pulses [8].

This paper's main conceptual contribution is a simple model of the sensory tranduction cascade that incorporates negative feedback and self-activation. The parsimonious model is based on a small set of reasonable assumptions.

Firstly, receptor activity is modeled heuristically with a logarithmic dependence on ligand concentration. The form of the equation is borrowed from a model of the bacterial chemotaxis signaling pathway [9]. In bacteria, the form of this equation reflects methylation and receptor desensitization at different concentrations of ambient ligand. My quibble with this paper is that adding the logarithmic dependence on ligand concentration by hand should automatically give rise to a broad dynamic range that "spans orders of magnitude". This may be how the system works, but remains a hypothesis.

Secondly, the authors posit switch-like activation of the channels. This self-reinforcing activity when receptors are activated is probably required for the pulsatile AWA activity. This may be related to the recent discovery of action potentials exhibited by AWA [3]. This point is addressed in the Supplement, but deserves discussion in the main paper. In the current formulation, the spikelike increase in neural activity would explicitly depend on receptor activity, whereas an action potential is modeled in terms of intrinsic voltage-dependent conductances. If I understand correctly, the link is made by connecting calcium dynamics to membrane potential. This hypothesis might be buttressed by analysis of the VGCC mutant (see appeal for more experiments below).

The authors reasonably model calcium dynamics as a function of channel activity (Equation

3). The heart of negative feedback is posited in Equation 4. The authors posit both calciumdependent and calcium-independent forms of inhibition. Equation 4 borrows ideas from models of bacterial chemotaxis. They specifically make the magnitude of a negative feedback pathway proportional to signaling activity [10] [11]. This feature is known to give rise to perfect adaptation in control theory. Here, inhibition is added in proportion to the fraction of activate receptors and is removed in proportion to the fraction of inactive receptors. These facts are essential to understanding the significance of Equation 4, but are buried in the Supplemental Information. This discussion belongs in the main paper.

Given the form of the equations, it is not surprising that abolishing the calcium-dependent component of the inhibition in Equation 4 that the system fails to adapt. When k5 = 0, Equation 3 becomes irrelevant, I becomes a direct function of Ra at steady-state, Ra becomes a direct function of L, and S will either saturate at its maximum or minimum values.

It is notable that the model predicts pulsatile AWA dynamics in response to graded stimuli, and adaptation to the first derivative of an applied stimulus. These experimental results were explored by the Zaslaver group in an earlier study [12]. It would help to have an intuitive explanation of why the model generates pulsatile dynamics and adaptation to the first derivative in the paper itself. EMBO likely does not have a space limitation.
The paper makes much of the robustness of the model, perfect adaptation, and how it works over many orders of magnitude of ligand concentration. But these features simply arise from the circuit topology that is borrowed from the robustness of models of bacterial chemotaxis. It does not seem possible to not get these features given their set of borrowed assumptions.

The paper's main experimental discovery concerns TAX-6/calcineurin. They find that calcium dynamics in the AWA neuron lacking TAX-6 function also lacks pulsatile activity. These mutants seemingly lack an important (and presumably calcium-dependent) negative feedback loop in the AWA neuron. Less robust effects are obtained with mutations in osm-6 and eat-16, which are thought to negatively regulate signal transduction. Arrestin-1, which inactivates GPCR signaling in other systems, is even more like wild-type.

I found the heat plots in Figure 3 much less informative than the line plots in Supplementary Figure 4. In the latter, it was much easier to see that, in most animals, osm-6 and eat-16 mutants exhibited pulsatile dynamics much like wild-type. But sometimes, these mutants exhibited calcium plateaus much like tax-6 mutants.

I believe that the effects of all of these mutations on AWA function are cell autonomous. But it would be straightforward to prove using cell-specific rescue of these genes in the AWA neuron. I think cell-specific rescue of TAX-6, in particular, would strengthen the paper. All of these genes are broadly expressed.

I would also welcome the additional analysis of mutations that affect the VGCC and TRPV channels that have been shown to operate in AWA [3] [2]. I would also welcome analysis of guanylyl cyclase mutants like grk-1 and grk-2, and other proteins that are known to affect G-protein signaling in sensory neurons like odr-3 [13] [4]. The authors are in an excellent position to comprehensively analyze the handful of signaling proteins that plausibly contribute to their model of signal transduction. The number of candidate genes in the literature is relatively small.

I would also welcome analysis of the effect of the TAX-6 mutation, their most compelling and important experimental discovery, on signal processing in at least one other cell type. I appreciate that the AWA is unique in its display of pulsatile dynamics. But TAX-6 is also widely expressed, and presumably contributes in a similar way to signal processing in AWC or ASH, other wellstudied neurons. I am delighted that a simple and parsimonious model suffices to explain calcium dynamics in AWA to a broad range of stimuli. Can the same or similar model (with different parameters or tweaked topology) explain signal processing in AWC, for example? A recent paper suggests that AWC resets its threshold in response to sustained odorant inputs[14], but I remain skeptical. I wonder if a more conventional dynamical systems model - like the one presented in this paper - would be equally effective.

At minimum, the contrast with models that have been used to describe AWC would be worthwhile. But the quantitative approach taken here, combined with mutant analysis, might provide a route to a more unified way of thinking about olfactory sensory neurons in the worm. And I would be interested in an alternative to (or reconciliation with) the adaptive threshold model presented by Levy and Bargmann.

In summary, I think the conceptual discoveries of the paper can be more clearly described and honestly presented. I think their discovery about TAX-6 is very important and deserves publication. But it could be strengthened by a broader understanding of other candidate genes in the signaling pathway of AWA, or a broader understanding of TAX-6 in other olfactory neurons. The authors are onto something. This is important work, but could be greatly improved without too much more work.

References
1. Rahi, S. J. et al. Oscillatory stimuli differentiate adapting circuit topologies. Nature Methods 14, 1010-1016. ISSN: 15487105 (2017).
2. Kahn-Kirby, A. H. et al. Specific polyunsaturated fatty acids drive TRPV-dependent sensory signaling in vivo. Cell 119, 889-900. ISSN: 00928674 (2004).
3. Liu, Q., Kidd, P. B., Dobosiewicz, M. & Bargmann, C. I. C. elegans AWA Olfactory Neurons Fire Calcium-Mediated All-or-

None Action Potentials. Cell 175, 57-70.e17. ISSN: 10974172 (Sept. 2018).
4. Fukuto, H. S. et al. G Protein-Coupled Receptor Kinase Function Is Essential for Chemosensation in C. elegans. eng. Neuron 42, 581-593. ISSN: 0896-6273 (Print) (May 2004).
5. Merritt, D. M. et al. Title: Arrestin-mediated Desensitization Enables Olfactory Discrimination in C. elegans. bioRxiv, 2021.05.02.439367 (2021).
6. Kuhara, A., Inada, H., Katsura, I. & Mori, I. Negative regulation and gain control of sensory neurons by the C. elegans calcineurin TAX-6. Neuron 33, 751-763. ISSN: 08966273 (2002).
7. Hajdu-Cronin, Y. M., Chen, W. J., Patikoglou, G., Koelle, M. R. & Sternberg, P. W. Antagonism between G(o)α and G(q)α in Caenorhabditis elegans: The RGS protein EAT-16 is necessary for G(o)α signaling and regulates G(q)α activity. Genes and Development 13, 1780-1793. ISSN: 08909369 (1999).
8. Larsch, J. et al. A Circuit for Gradient Climbing in C. elegans Chemotaxis. Cell Reports 12, 1748-1760. ISSN: 22111247 (2015).
9. Tu, Y., Shimizu, T. S. & Berg, H. C. Modeling the chemotactic response of Escherichia coli to time-varying stimuli. Proceedings of the National Academy of Sciences of the United States of America 105, 14855-14860 (Sept. 2008).
10. Barkai, N. & Leibler, S. Robustness in simple biochemical networks. Nature 387, 913-917. ISSN : 00280836 (June 1997).
11. Yi, T. M., Huang, Y., Simon, M. I. & Doyle, J. Robust perfect adaptation in bacterial chemotaxis through integral feedback control. Proceedings of the National Academy of Sciences of the United States of America 97, 4649-4653. ISSN: 00278424 (2000).
12. Itskovits, E., Ruach, R. & Zaslaver, A. Concerted pulsatile and graded neural dynamics enables efficient chemotaxis in C. elegans. 9. ISSN: 20411723. doi:10.1038/s41467- 01805151-2 (2018).
13. Roayaie, K., Crump, J. G., Sagasti, A. & Bargmann, C. I. The Gα protein ODR-3 mediates olfactory and nociceptive function and controls cilium morphogenesis in C. elegans olfactory neurons. Neuron 20, 55-67. ISSN: 08966273 (1998).
14. Levy, S. & Bargmann, C. I. An Adaptive-Threshold Mechanism for Odor Sensation and Animal Navigation. Neuron 105, 534-548.e13. ISSN: 10974199 (2020).

Reviewer #3:

In this manuscript, Ruach and colleagues examine the molecular mechanisms underlying the coding of odor in the AWA chemosensory neuron of C. elegans. The work elegantly combines the experimental inspection of the AWA neuron in wild-type and mutant backgrounds with modeling to reveal the critical role of feedback inhibition on the GPCR signaling involved in the detection of odor stimluli. The authors developed a parsimonious ODE model for the GPCR signaling cascade coupled to the TRPV and the voltage-gated ion channels responsible for the depolarization dynamics of the neuron, including its adaptive and pulsatile properties. I find the parsimonious model impressive: instead of trying to model the GPCR signaling pathway in detail, the authors opted for a coarse-grained representation that aims to abstract essential features of complex regulatory processes. As a result, modeling genuinely assists the functional dissection of the signal transduction cascade without getting bogged down with the biophysical description of mechanisms that remain poorly understood.

The parsimonious model captures essential features of the AWA response: calcium-dependent and calcium-independent inhibition, exact adaptation. One of the main findings of the study is that the mechanism by which the AWA neuron adapts to odorant signals relies on a negative feedback loop. The authors report that Calcineurin tax-6 creates an inhibitory feedback on the GPCR pathway. The parsimonious model helps establish that the inhibitory feedback mediated by tax-6 is calcium-independent. Although the exact mode of action of Calcineurin tax-6 remains to be determined in future work, the characterization of its loss of function on the AWA response represents an important step toward understanding how a single neuron perform complex computations to efficiently encode complex stimulus patterns necessary to efficiently navigate chemosensory gradients.

The manuscript is clearly written. I enjoyed all the references made to bacterial chemotaxis, which represents a source of inspiration to conceptualize sensory coding and formulate mechanistic hypotheses. Key results described in the main figures are supported by extra material in the supplementary material. I nonetheless believe the authors should strengthen the evidence supporting some of their claims. Below, I listed a series of concerns and suggestions for the authors to improve the manuscript. None of these concerns are major, though some suggestions might require additional experimental work.

1) The work argues about the ethological relevance of pulsatile coding in the AWA chemosensory neuron - this idea builds on a previous publication from the same lab (Itskovits et 2018). In particular, the authors show that the AWA neuron of tax-6 mutants are unable to generate pulsatile activity and to achieve exact adaption in response to natural stimuli. If the calcium pulses control the reorientation dynamics of the worm, the implications of the study is that tax-6 mutants should have a severe deficit in chemotaxis. Would the authors predict that reorientation maneuvers would be inhibited in the tax-6 mutants? It would be important to validate this prediction experimentally.

From the seminal study of Pierce-Shimomura and Lockery 1999, the typical duration of a "run" is on the order of ~20s. By contrast, the pulses of calcium in the AWA neuron tend to take place on a timescale of ~100s. What explains this apparent mismatch between the neuronal activity and the reorientation behavior?

2) The work focuses on two features of the AWA response: (1) exact adaptation in response to a single step of the stimulus; (2) adaptation of pulsatile responses to the magnitude of the gradient's first derivative. Reproducing these properties was used to define the parameters of the parsimonious ODE model. It would be helpful if the authors could clarify in the main text how the parameter optimization was achieved, before introducing the perturbative analysis of the model's parameters. Strikingly, the exact adaption of the AWA response is only evaluated based on odor pulses (diacethyl) added to an odor background of 1.15mM. The adaption to the first derivative is assessed on a single sigmoid function. Given that the study relies on the goodness of fit of the model with experimental observations, the authors should extend their validation to odor steps added to different background concentrations (along the lines of what is done in Supp. Fig. 1d). They should also use patterns of odor stimulation where the first derivative varies. This analysis would validate the claim that "the amplitude and the frequency of the pulses correlate with the gradient's first derivative and adapt to it" (page 7).

3) The authors discuss the "periodic skipping" of calcium pulses in the AWA neuron upon odor pulses at high frequencies (page 8). The skipping is not reproduced by the simulations of Supp. Fig. 1a-c. While the calcium pulses become attenuated, they do not disappear. Could the authors explain why might be missing in the model to fully account for this behavioral observation? Likewise, the relaxation dynamics of the tax-6 mutant predicted by the model is much shorter than that observed experimentally in Fig 6a. What regulatory processe(s) might be missing to capture the relaxation kinetics more accurately in the tax-6 mutants?

4) Could you define what you mean by "adaptation" and "habituation"? Habituation is generally defined as a decrease in behavioral response that is independent of sensory adaption. I am not convinced that this definition applies to the long-term loss of sensitivity reported following a prolonged odor pulse or a series of odor pulses. This phenomenon appears to fit within the realm of sensory adaptation, unless you expect it to depend on higher order processes that involve other neurons. You might need to revise your terminology accordingly.

Typos:
- The reference to "Fechner" finishes by N.D. and not a date.
- Some of the references that include multiple authors are cited as single authored.
- Page 3, 3rd paragraph: should "none-active" receptors read "inactive"?

A point by point reply to the reviewer's comments

We thank all reviewers for the instructive most helpful comments. We had followed all of the suggestions and considerably improved the manuscript. In particular, to verify that the intricate AWA dynamics is cell-autonomously mediated via the TAX-6 negative feedback, we have expressed TAX-6 in an AWA-specific manner. This rescue restored the pulsatile activity and reduced the amplitude to WT levels, thus, corroborating our working model and previous observations that TAX-6 mediates the negative feedback in a cell-autonomous manner. In addition, we also tested an additional tax-6 mutant strain (*ok2065*) which essentially recapitulated the results obtained with the original *tax-6*(*p675*) mutant used. We also analyzed dynamics in Galpha (*odr-3*) and GRK (*grk-2*) mutants that are known to be expressed in AWA neurons. Results of these mutants further supported our working model.

We have also reanalyzed the data as requested and provided results for additional modeling conditions that together further supported our experimental results.

Reviewer #1:

In a previous study, the authors showed that, during olfactory chemotaxis by C. elegans, AWA olfactory neurons exhibit pulsatile responses when exposed to a continuous increase in the concentration of an attractive odorant diacetyl. In this study, they assumed molecular feedback circuits in AWA neurons and reproduced the pulsatile activity based on mathematical modeling. They further showed that the calcium-dependent protein phosphatase calcineurin TAX-6 plays an important role in this activity. However, although I agree that the model is reasonable and represents a reasonable reproduction of the wild-type pulsatile responses, I still have a number major and minor concerns, which I believe should be addressed prior to the publication of this work. These are listed below.

Major:

(1) In their genetic analyses, the authors used only a single mutant allele for each gene, and no rescue experiment was conducted. It is thus possible that the phenotypes described in the manuscript may be caused by side mutations. In order to clarify this issue, it is necessary to use at least two alleles for each gene or alternatively conduct rescue experiments.

Since this manuscript focuses on the negative feedback of TAX-6/Calcineurin, we have now added to the analysis an additional mutant allele of this gene, *ok2065*. This mutant shows a very similar phenotype to the *p675* allele (reported in our original manuscript): Both are impaired in pulsatile activity and fail to reach exact adaptation (Figure 3).

Importantly, we have now conducted a rescue experiment in which we expressed TAX-6 exclusively in the AWA neurons (there is also expression in one more distal cell at the tail). Analysis of this line restored the pulsatile activity, the amplitude, and the exact adaptation within the AWA neruons, thus

supporting our results that TAX-6 plays a major role in the negative feedback in a cell-autonomous manner.

We also made sure that all mutants used in this study were backcrossed with the N2 wild type strain at least five times (this was done either by us or by the researchers that originally reported the mutant strain). While not eliminating all mutations, these repeated crosses reduce possible side effects.

(2) Related: The authors provide no clear evidence of cell autonomy. To the best of my knowledge, the possibility that diacetyl is sensed by neuron(s) other than AWA and the neuron(s) affecting AWA activity has not been disproved.

The evidence for the cell autonomous activity stems mainly from our previous publication (Itskovits et al Nat Comm 2018) where we showed that the pulsatile activity remains intact in both neurotransmitter- and neuropeptide-secretion mutants (*unc-13 and unc-31*, respectively).

We have now highlighted this important point in the introduction, page 3, 2nd paragraph:

"A single pair of chemosensory neurons, the AWA neurons, exhibits a range of coding features in response to diacetyl, a chemo-attractive food cue secreted from bacteria in decomposing fruits(Choi et al, 2016). Smooth and slowly increasing gradients of diacetyl lead to a pulsatile activity in the AWA neurons, where the frequency and the amplitude of the pulses increase the greater is the temporal derivative of the stimulus (Itskovits et al, 2018). This pulsatile activity was also observed in mutant animals, defective in neurotransmitter and neuropeptide secretion, suggesting that these sensory neurons may implement such intricate computation in a cell-autonomous manner. "

Moreover, as described above, we have now generated a cell-specific rescue line expressing TAX-6 exclusively in the AWA neurons (we also observed an expression in one cell at the tail region). In the newer version of the manuscript we show that rescuing the gene in the AWA neurons restores the fine dynamics in the AWA neurons (Figures 3-4), further supporting our conclusion that this is a cell-autonomous process.

(3) A further related issue concerns the authors' assumption of a molecular feedback loop consisting of GPCR -> G_alpha -> TRP channel -> VGCC -> calcium entry -> calcineurin -| GPCR or G_alpha. Although this scenario is plausible in terms of molecular and cellular biology, the assumed operation of this mechanism would be dependent on all the participating molecules being localize in very close proximity; that is, at the sensory ending of the neuron. Is this true? Certainly, this assumption would be reasonable for the GPCR, G_alpha, and TRP channels; however, as far as I understand, VGCC is not considered to localizes at the worm's sensory ending. Also, what about TAX-6? Moreover, they monitored calcium dynamics within the cell body, rather than the sensory ending. Thus, I would like the authors to present some experimental evidence showing that VGCC and TAX-6 indeed work in the feedback loop.

The evidence for VGCC functioning in the feedback loop is based on a previous report showing that this channel mediates diacetyl-induced AWA activation. Larsch et al (Cell Reports 2015, Figure 4a) showed that a mutant of the alpha1 L-type voltage-gated Ca channel (*egl-19*) is defective to show Ca fluxes in response to a range of diacetyl concentrations. We therefore relied on these experiments and positioned VGCC in our feedback loop model as a key factor for initiating the Ca flux.

In addition, while not in the AWA neurons but in the adjacent chemosensory neuron ASEL, *egl-19* was shown to be essential for sensory-evoked regenerative depolarization in dendrites (Shindou et al, Scientific Reports 2019). It is also shown that this channel is localized to the dendrites as well as to the axon and the cell body of this neuron. Thus, in *C. elegans* chemosensory neurons, VGCCs are localized to dendrites.

As for TAX-6, Kuhara et al (Neuron 2002) showed that a translational fusion of this protein is also found in the cilia of the AFD sensory neurons (Figure 3J-K in that reference). Also, our translational fusions for AWA-specific rescue show that TAX-6 is expressed in the entire cell, including the dendrites. Furthermore, our imaging analyses show that Ca pulses are prominent in the dendrites as well. We added an Appendix movie S1 that shows these dynamics.

Taken together, these findings indicate that all the signaling components may be localized in proximity along the dendrite, presumably also in the cilia, to drive the negative feedback loop. We have now added a paragraph to the discussion that details all of the findings provided above to explain how this circuit may be operating within chemosensory dendrites. Page 22, last paragraph:

*"Clearly, this negative feedback necessitates that all the components will be localized to the cilia, the dendrite ending region. Indeed many of the components had been shown to be localized to the dendrites and cilia of C. elegans sensory neurons, including the ODR-10 GPCR, the Gα subunit ODR-3, the VGCC EGL-19, and TAX-6/Calcineurin (Kuhara et al, 2002; Sengupta et al, 1996; Shindou et al, 2019; Mukhopadhyay et al, 2008; Roayaie et al, 1998). Supporting this hypothesis, we observed calcium pulsatile activity in the AWA dendrite and cilia, activity which was synchronized with the calcium transients observed in the cell soma (**Appendix Movie S1**)."*

(4) The title of the manuscript includes the expression "A GPCR negative feedback loop." However, what is the evidence for the involvement of GPCR? Is it because diacetyl is sensed by the GPCR ODR-10? In my understanding, the arr-1 mutation did not significantly affect the AWA response. Thus, the defective phenotype observed only in (a single allele of) eat-16 is used to support the claim, whereas another component of GPCR signaling, arrestin, is not involved. I do not believe this constitutes sufficient evidence to corroborate the authors' contentions and thus, as discussed above, further supporting evidence is needed.

We have now analyzed additional mutants, defective in key genes that belong to the GPCR signaling pathway, including the Galpha subunit (*odr-3*), and the G protein-coupled receptor (*grk-2*). Analysis of their activities in response to diacetyl gradients shows these genes are involved in

mediating the intracellular signals (Figure 3). For example, no response at all in *odr-3* mutants and hyperactivation with no clear pulses in the absence of GRK-2.

Furthermore, in our revised manuscript, we emphasize that the TAX-6 negative feedback might be affecting along the path between the GPCR and the activation of TRPV channels. On page 23, last paragraph:
*"This positions the TAX-6/Calcineurin to mediate inhibition along the pathway between the GPCR to the TRPV (**Figure 1,7**)."*

Consequently, we also changed the title to:
'A negative feedback loop in the GPCR pathway underlies efficient coding of external stimuli'

We speculate that the lack of a clear phenotype in *arr-1* mutants is due to the fact that arrestin is usually recruited after longer time periods to mediate GPCR internalization that may promote extended forms of sensory adaptation.

(5) What is the physiological significance of the dynamic AWA calcium response to a gradual increase in odor stimulus? It is OK that animals sense several minutes (~400 s) of continuous gradual increase in odor concentration. That happens in general. Then, the data presented by the authors indicate that AWA shows 10 times of transient (pulsatile) calcium increases every 30 to 60 s. How does such a series of AWA activations affect the worm's behavior?

The significance of these pulses in the AWA neurons is that each pulse promotes forward movement of the animal. We have demonstrated this causal relationship in our previous manuscript (Itskovits et al Nat Comm 2018). Thus, in animals sensing increasing first derivatives of the attractive odorant, the frequency of the pulses increases, thereby maintaining the trajectory course of the animal straight. This pulsatile activity is analogous to the runs epochs during the 'run and tumble' biased random walk strategy.

A time course of several/many minutes in which animals sense and respond to chemical stimuli is a relevant time frame given their natural habitats and speed. For example, worms are typically extracted from rotten fruits where they seek bacteria that serves as their food source. These bacteria were shown to emit such odorant cues that attract the worms.

We have now added this explanation to the introduction, page 3 last paragraph:
"Behaviorally, AWA activity facilitates forward locomotion, while a decrease in AWA activity promotes turning events(Itskovits et al, 2018; Larsch et al, 2015). Thus, a pulsatile activity dictates a run and tumble strategy, similar to the biased-random walk behavior in E. coli(Pierce-Shimomura et al, 1999)."

Collectively, although the overall model appears elegant, the link between the model components and molecular activity, as well as its physiological significance, have yet to be sufficiently explained and/or supported by evidence.

In this revised version, we have provided a detailed explanation that better links between the model, the experimental results, and the overall physiological significance (as noted also in the previous point).

In the results, page 8, paragraphs 2 and 3, we detail how the model explains the observed results: *"How does the circuit model translate an increasing gradient…"*

Furthermore, we now explain that the model is geared to qualitatively explain the data, rather than to provide a detailed quantitative parametric description of the molecular processes. In the discussion, page 25:
"When constructing the model, we strived to simplify the detailed signaling cascades, focusing on the key computational components that underlie the observed coding. For this, we grouped components acting together or sequentially and abstracted parts of the system's dynamics. For example, we modeled activities of TRPVs and VGCCs as a single self-activating component."

(6) In the title and early part of the Introduction, said the authors use the term "coding," which, in my understanding, is taken to indicate relaying environmental information to downstream neurons. However, there is no description of these downstream neurons.

By using the term coding, we refer to the computational operations performed within the AWA neuron to decode the complex dynamics of the external stimulus. In other words, how diacetyl gradients are translated into AWA activity. Clearly, AWA activity is then relayed to activate downstream postsynaptic neurons to eventually enact the proper behavior. However, in this manuscript, we did not analyze these downstream neurons.

(7) p. 5 middle: The authors claim that "The pulsatile activity correlates with the first derivative of the gradient and adapts to the magnitude of the first derivative." I was unable to grasp the meaning of this sentence, particularly from a quantitative perspective, even after having gone through their previous paper (Itskovits et al., 2018). On the basis of the information presented in Figure 2, for the step-like stimulus (panel a), I understand that the response ($\Delta F/F0$) is quite similar to dL/dt. However, for a gradually increasing stimulus, how is $\Delta F/F0$ "correlated" with the first derivative? The shapes of dL/dt and $\Delta F/F0$ appear to differ substantially. During the early phase, dL/dt was very small and close to zero; however, $\Delta F/F0$ was already high (indeed at almost its highest). How does then this pattern change thereafter? Do the authors claim that when dL/dt increases, the peak magnitude decreases to a certain extent and/or the intervals of the peaks become longer? I do not agree with this, given that just a single example is shown. Actually individual responses are shown in Sup. Fig S4, although there are only eight examples, and interpretation of the results could differ owing to lack of quantitative analysis. I was unable to find a quantitative analysis based on actual AWA responses even in the previous 2018 paper. Thus, I would like to conclude that the authors' claim (the quoted sentence at the top) is insufficiently supported by the data presented and would thus request more quantitative analysis of the pulsatile activity characteristics in wild-type animals.

We have now changed the sentence to better explain what we meant: It is the frequency and the amplitude of the pulses that are correlated with the first derivative of the stimulus. This can be seen

when averaging a population of animals and responses. On longer time scales, this activity adapts to the magnitude of the stimulus first derivative. We have shown this with extensive experimental evidence in our previous manuscript (Itskovits et al 2018), and herein, we reproduce these results and provide a mechanistic explanation for these features. We have now rephrased this sentence to better explain what we meant by this correlation. In the first paragraph of the results, on page 5:

*"(4) The mean frequency and the amplitude of the pulses, induced by smooth increasing stimuli, correlates with the first derivative of the gradient and also adapts to it (**Figure 2B** and (Itskovits et al, 2018)). Thus, the steeper the change in the gradient (higher first derivative), the higher are the frequency and the amplitude of the neural activity. On longer time scales, this activity adapts to the magnitude of the stimulus first derivative, such that the frequency and the amplitude of the pulses decrease if not facing increasing first derivative changes in the stimulus."*

(8) p. 6: In my understanding, the key to this study is the modeling of AWA activity using equations 1 to 4, which were in fact developed for bacterial chemotaxis by a different group. Then, what is the originality of this study? It would appear that the authors simply applied a well-established method to examine C. elegans neuronal activity.

Indeed, the negative feedback idea was originally developed for bacterial chemotaxis (as also noted in the ms). Nevertheless, this is the first report to demonstrate how such a negative feedback can be embedded within the GPCR signaling pathway. Moreover, we have shown how one can integrate the known integral feedback mechanism into a pulsing neuron to exert several functions, including a pulsatile activity that adapts to the first derivative, in a cell-autonomous manner.
To our knowledge, this is the first report demonstrating such a mechanism in an animal's sensory neuron.

(9) Related: p. 8, "Our model predicts..." The authors claim that certain features of AWA responses are reproduced in their model. However, I was not convinced that the responses of AWA are characterized by such features, owing to the lack of quantitative evidence.

In this sentence we refer to experimental evidence obtained by other groups, and which can be qualitatively captured by our model. We now better explain this point and made sure to provide the exact references for these experimental observations. Page 8, last paragraph.:

*"Our model qualitatively captures additional functional features observed in AWA response dynamics (**Appendix Figure S1**). In response to repetitive high-frequency steps of the stimulus, inhibition removal may be too slow, and hence, activity would not be observed in response to all repetitive stimulations, a phenomena known as periodic skipping (compare **Appendix Figure S1B,C** and see also experimental evidence in (Larsch et al, 2015; Rahi et al, 2017)). Furthermore, in response to short on-steps, our model predicts gradual adaptation: a decrease in response amplitude between consecutive steps (**Appendix Figure S1A**), which was also observed experimentally (Larsch et al, 2015; Rahi et al, 2017)."*

(10) p. 8, "The model implements..." (Sup Fig. S2): I agree that the k5 pathway is essential. However, k6 may not play a prominent role. Moreover, the authors only show model responses to a step-like stimulus. It would be informative to examine the responses of the model to a gradual stimulus in the absence of k5 or k6.

Indeed, k6 may not play a prominent role in the negative feedback, and we did not assay mutants which affect k6, so we decided to withdraw this figure from the manuscript. We nevertheless added it to the model to account for the known Ca-independent inhibiting processes. We now provide simulation results for the k5=0 case, which essentially mimics a *tax-6* mutant, the key factor that we focus on in this ms.

(11) p. 8, "Notably,,," (Sup Fig. S3): Although the authors show the ranges of parameters, they do not describe the outcomes of the models with different parameters. What does this figure purport to show?

The purpose of the figure was to show that the model outcomes are not sensitive to the exact values of the different parameters. Even when modulating each of the parameters (separately) by an order of 10-fold, the main features are still observed, essentially, exact adaptation following a step and pulsatile activity that adapts to the first derivative during a smooth sigmoidal gradient.

We have now added examples of five random simulations with varied parameters (Appendix Figure S3D-F) which demonstrate the outcome following both an on step and a gradient of the stimulus. While the fine dynamics and the frequency of the pulses changes, the global features mentioned above are maintained.

(12) I understand that the long-lasting response of AWA neurons in tax-6 mutants can be explained by the lack of a Ca++-dependent feedback pathway. However, does the model explain the large initial response to the step-like stimulus in tax-6 mutants? This does not appear to have been reproduced in the model (Fig. 6b). This should not be taken as a criticism, but would be of interest to know.

Indeed, while we do observe a higher amplitude of *tax-6* mutants (when compared to wt) when modeling the on-step, this increase is not as striking as seen in the experimental results. This is due to the specific parameters chosen to use for the simulations. For example, choosing a higher k5 (the constant for ca-mediated inhibition of the receptors) will provide a greater difference in the amplitude between wt and *tax-6* mutants. Thus, while qualitatively the model agrees with the experimental data, the exact quantitative outputs may differ.

Minor:

(1) There are too many typographical errors to point out individually. In my opinion, the submitted manuscript has not been sufficiently well prepared. I am constantly disappointed by authors (not just the authors of this particular manuscript, but those in general) who prematurely submit poorly constructed manuscripts, in the expectation that reviewers will play the role of the grammar-check function of a word processing application.

We have now fixed the typos.

(2) p. 2-5: In my opinion, the Introduction is too long, in excess of 1,000 words. Moreover, in addition to the length, it contains information that is not strictly related to the Results. I accordingly recommend reducing the word count to less than 700.

We tried to make the introduction more concise but felt that the broad scope of *C.elegans* experimental system together with modeling concepts borrowed from other disciplines requires detailing on each.

Reviewer #2:

C. elegans performs olfactory behaviors using a small number of sensory neurons in a small nervous system. Each sensory neuron is endowed with substantial functionality to allow the animal to sense absolute and relative concentrations of many different ambient chemicals. Much is known about signal transduction in E. coli chemotaxis where all molecules in its biochemical pathway have been identified and dissected. Models for bacterial chemotaxis are rigorous and well-tested.

C. elegans olfaction is beginning to be explored in similarly quantitative ways. This paper represents a substantial contribution in this growing research area. Worm genetics has identified a number of molecules that contribute to signal transduction in the G-protein coupled pathways in their OSNs. One GPCR has been identified (ODR-10), and roles for a number of commonly studied molecules like calcineurin, guanylyl receptor kinases, and arrestin have been assigned using behavioral phenotypes.

With the rise of quantitative physiological methods with single-cell resolution (mostly calcium imaging), it is now possible to pursue integrated dynamical models of the molecular pathways that underlie olfactory processing. GPCR signaling pathways have been intensively and successfully modeled in other systems, notably vertebrate photoreception. It would be amazing to capitalize on the strengths of worm genetics to better understand these pathways in olfactory sensory neurons.

The authors focus on the AWA sensory neuron. AWA senses diacetyl over a large concentration range (7 orders of magnitude). In response to a stepwise increase of diacetyl concentration, AWA exhibits a large increase in calcium levels that perfectly adapts to prestimulus levels. In response to a gradual change in diacetyl concentrations, AWA exhibits pulsatile dynamics.

Rahi et al. argued that the AWA olfactory response exhibits adaptation by negative feedback
based on calcium imaging in response to a series of diacetyl pulses [1]. We know many molecules that work between diacetyl detection by ODR-10 and calcium dynamics. These include TRPV channels [2] and voltage-gated calcium channels [3]. Several molecules associated with G-protein coupled pathways in AWA and other OSNs have been identified. These include kinases and arrestin that typically downregulate GPCRs (but which lead to unusual and interesting phenotypes in C. elegans [4], [5]), TAX-6/calcineurin (which has been suggested to negative regulate sensory

signaling in worms [6]), EAT-16 (which negatively regulates activated G-protein pathways [7], and OSM-6 which AWA needs for habituation to repeated diacetyl pulses [8].

This paper's main conceptual contribution is a simple model of the sensory tranduction cascade that incorporates negative feedback and self-activation. The parsimonious model is based on a small set of reasonable assumptions.

Firstly, receptor activity is modeled heuristically with a logarithmic dependence on ligand concentration. The form of the equation is borrowed from a model of the bacterial chemotaxis signaling pathway [9]. In bacteria, the form of this equation reflects methylation and receptor desensitization at different concentrations of ambient ligand. My quibble with this paper is that adding the logarithmic dependence on ligand concentration by hand should automatically give rise to a broad dynamic range that "spans orders of magnitude". This may be how the system works, but remains a hypothesis.

Indeed, we rely on this conjecture to achieve two important features of the system: the logarithmic dependence provides the broad dynamic range, and at the same time, together with the linear inhibition, it underlies the adaptation to the first derivative. This is explained in discussion. Page 22

“Adaptation to the first derivative of the gradient and logarithmic coding are achieved by the inherent activity mode of GPCRs(Olsman & Goentoro, 2016). These receptors are logarithmically facilitated by the ligand, but linearly inhibited by intracellular components.”

Secondly, the authors posit switch-like activation of the channels. This self-reinforcing activity when receptors are activated is probably required for the pulsatile AWA activity. This may be related to the recent discovery of action potentials exhibited by AWA [3]. This point is addressed in the Supplement, but deserves discussion in the main paper. In the current formulation, the spikelike increase in neural activity would explicitly depend on receptor activity, whereas an action potential is modeled in terms of intrinsic voltage-dependent conductances. If I understand correctly, the link is made by connecting calcium dynamics to membrane potential. This hypothesis might be buttressed by analysis of the VGCC mutant (see appeal for more experiments below).

Experiments of a VGCC mutant (*egl-19*) had been performed by Larsch et al (Cell reports 2015, ref #8 below). These experiments revealed that *egl-19* mutants do not show calcium responses when exposed to diacetyl. Thus, these channels are clearly necessary for the calcium influx. We think that the spikes observed in AWA (Liu et al Cell 2019) and calcium pulses that we observe in response to diacetyl gradients are related but not the same. Each calcium pulse may be initiated as a result of a spike train resulting from voltage-dependent conductance. However, the longer temporal decay of intracellular calcium (on the order of tens of seconds) may be the crucial factor playing in the negative feedback of the GPCR pathway. This longer time scale is also crucial for the behavioral output, where animals are moving straight as long as AWA is active (shown in our previous manuscript, Itskovits et al 2018).

We have now added these considerations to the manuscript. Page 25, 3rd para.:

*“As AWA neurons generate action potentials (Liu et al, 2018), a more realistic model would include the positive feedback loop between the VGCCs, the potassium channels driving the action potential downstroke, and the membrane potential (dictated in part by calcium). It is conceivable that the calcium pulses may be initiated as a result of a spike train resulting from voltage-dependent conductance. However, these spikes operate on shorter time scales than the calcium transients and the longer temporal decay of intracellular calcium (on the order of tens of seconds, **Figure 3**) may be crucial for the negative feedback of the GPCR pathway. This longer time scale also dictates behavioral outputs, as animals maintain a straight trajectory during the pulse when AWA neurons are active, and turn once the pulse is terminated (Itskovits et al, 2018).”*

The authors reasonably model calcium dynamics as a function of channel activity (Equation
3). The heart of negative feedback is posited in Equation 4. The authors posit both calciumdependent and calcium-independent forms of inhibition. Equation 4 borrows ideas from models of bacterial chemotaxis. They specifically make the magnitude of a negative feedback pathway proportional to signaling activity [10] [11]. This feature is known to give rise to perfect adaptation in control theory. Here, inhibition is added in proportion to the fraction of activate receptors and is removed in proportion to the fraction of inactive receptors. These facts are essential to understanding the significance of Equation 4, but are buried in the Supplemental Information. This discussion belongs in the main paper.

Agreed, and we have now provided these key points that are crucial to understanding the model. In the results part that explains the equations rationale. Page 7:
*“Finally, equation 4 describes the circuit negative feedback (), where calcium-dependent (e.g., GRKs, denotes by the arrow) and calcium-independent (e.g., PKA/PKC, denoted by the arrow) pathways enhance inhibition, and a first-order removal term suppresses it. Borrowing the exact adaptation concepts developed for the E. coli chemosensory circuit, we posit that inhibition is proportional to the fraction of active receptors (), and the removal term is proportional to the fraction of inactive receptors (). A detailed description and analysis of the mathematical model are available in **Appendix Note S1 parts 1-3 and Appendix Table S1**.”*

And on page 8:
“The high intracellular calcium concentration enhances a quick inhibition (eq. 4), thus reducing the fraction of active receptors below the critical threshold, (eq. 2), in which case, the pulse is terminated by exponential removal of the calcium (eq. 3). Relaying the signaling output, , to directly facilitate inhibition is known as an integral feedback and underlies exact adaptation (Yi et al, 2000).”

Given the form of the equations, it is not surprising that abolishing the calcium-dependent component of the inhibition in Equation 4 that the system fails to adapt. When k5 = 0, Equation 3 becomes irrelevant, I becomes a direct function of Ra at steady-state, Ra becomes a direct function of L, and S will either saturate at its maximum or minimum values.
It is notable that the model predicts pulsatile AWA dynamics in response to graded stimuli, and adaptation to the first derivative of an applied stimulus. These experimental results were explored by the Zaslaver group in an earlier study [12]. It would help to have an intuitive explanation of why the

model generates pulsatile dynamics and adaptation to the first derivative in the paper itself. EMBO likely does not have a space limitation.

We have now added these intuitive explanations for the pulses to the paper. On page 8:

*"How does the circuit model translate an increasing gradient of the stimulus input into a pulsatile calcium activity, an output that both increases with the input's derivative and adapts to it? Consider a smooth slowly-increasing stimulus: At the beginning, calcium levels are close to baseline levels such that the inhibition is relatively constant (eq. 4). As stimulus concentration gradually increases, the fraction of active receptors also rises (eq. 1). Once it crosses a threshold value, , TRPV, followed by the VGCCs, will open (eq. 2), resulting in a calcium pulse (eq. 3). The high intracellular calcium concentration enhances a quick inhibition (eq. 4), thus reducing the fraction of active receptors below the critical threshold, (eq. 2), in which case, the pulse is terminated by exponential removal of the calcium (eq. 3). Relaying the signaling output, , to directly facilitate inhibition is known as an integral feedback and underlies exact adaptation (Yi et al, 2000). As time passes and the stimulus concentration increases, this process will repeat itself to produce an additional calcium pulse. Since receptors' activation is logarithmically dependent on the input, the change required in the input levels to elicit a consecutive pulse becomes increasingly larger. For a linear gradient, this implies that the time interval between consecutive pulses will increase, effectively leading to an adaptation to the input gradient. In fact, a detailed mathematical analysis shows that the time interval between consecutive pulses increases exponentially with the pulse index number and is inversely proportional to the linear gradient derivative (**Appendix Note S1 part 3**)."*

The paper makes much of the robustness of the model, perfect adaptation, and how it works over many orders of magnitude of ligand concentration. But these features simply arise from the circuit topology that is borrowed from the robustness of models of bacterial chemotaxis. It does not seem possible to not get these features given their set of borrowed assumptions.

True, and we now tried to put all these points in the concise paragraph provided in the above point to show how all these features can be implemented within a single neuron via the negative feedback loop of the GPCR signaling pathway. We'd like to think this is part of the elegance in the model and the manuscript.

The paper's main experimental discovery concerns TAX-6/calcineurin. They find that calcium dynamics in the AWA neuron lacking TAX-6 function also lacks pulsatile activity. These mutants seemingly lack an important (and presumably calcium-dependent) negative feedback loop in the AWA neuron. Less robust effects are obtained with mutations in osm-6 and eat-16, which are thought to negatively regulate signal transduction. Arrestin-1, which inactivates GPCR signaling in other systems, is even more like wild-type.
I found the heat plots in Figure 3 much less informative than the line plots in Supplementary Figure 4. In the latter, it was much easier to see that, in most animals, osm-6 and eat-16 mutants exhibited pulsatile dynamics much like wild-type. But sometimes, these mutants exhibited calcium plateaus much like tax-6 mutants.

We appreciate that there are pros and cons to showing either the heat or the line plots. Given that we have now extended the experimental data, it seemed to us better to keep the more packed style of the heat plots in the main text and provide the full line plots for all experiments in the Appendix part. Furthermore, in the heat plot, we can squeeze in both absolute and normalized values of the data such that both amplitudes and pulses can be compared across the different strains.

I believe that the effects of all of these mutations on AWA function are cell autonomous. But it would be straightforward to prove using cell-specific rescue of these genes in the AWA neuron. I think cell-specific rescue of TAX-6, in particular, would strengthen the paper. All of these genes are broadly expressed.

We have now followed this recommendation and performed a cell-specific rescue of TAX-6 within the AWA neurons (in the strain we generated, there is an expression in an additional distal cell in the tail which is irrelevant).
Analysis of this rescue strain shows that the pulsatile activity is restored (in fact, the frequency is higher, possibly due to overexpression) and the amplitude is also reduced to WT levels (Figures 3-4). This indicates that TAX-6 may indeed be functioning in the negative feedback in a cell autonomous manner.

I would also welcome the additional analysis of mutations that affect the VGCC and TRPV channels that have been shown to operate in AWA [3] [2]. I would also welcome analysis of guanylyl cyclase mutants like grk-1 and grk-2, and other proteins that are known to affect G-protein signaling in sensory neurons like odr-3 [13] [4]. The authors are in an excellent position to comprehensively analyze the handful of signaling proteins that plausibly contribute to their model of signal transduction. The number of candidate genes in the literature is relatively small.

We have now followed these suggestions and analyzed mutant strains defective in *odr-3* and *grk-2* (Figure 3). We also added an additional *tax-6* mutant allele *(ok2065)*, to verify the key role of TAX-6 in the negative feedback (Figures 3-4).
Since Larsch et al already tested VGCC and TRPV mutants (e.g., *egl-19, ocr-1/ocr-2, osm-9*), finding that they lack calcium transients in response to sharp diacetyl on steps, we did not expect these mutants to show any dynamics in response to smooth slowly-increasing gradients.

As expected, mutants defective in *odr-3* (the immediate downstream component of the GPCR that eventually triggers TRPV and VGCC) lacked any calcium transients when facing a diacetyl gradient. Mutants, defective in *grk-2*, showed calcium dynamics that resemble more the *tax-6* mutants than the WT. These results further underscore the role of these genes in the GPCR negative feedback loop, and in particular demonstrating that GRKs are directly involved in the negative feedback.

I would also welcome analysis of the effect of the TAX-6 mutation, their most compelling and important experimental discovery, on signal processing in at least one other cell type. I appreciate that the AWA is unique in its display of pulsatile dynamics. But TAX-6 is also widely expressed, and presumably contributes in a similar way to signal processing in AWC or ASH, other wellstudied

neurons. I am delighted that a simple and parsimonious model suffices to explain calcium dynamics in AWA to a broad range of stimuli. Can the same or similar model (with different parameters or tweaked topology) explain signal processing in AWC, for example? A recent paper suggests that AWC resets its threshold in response to sustained odorant inputs[14], but I remain skeptical. I wonder if a more conventional dynamical systems model - like the one presented in this paper - would be equally effective.
At minimum, the contrast with models that have been used to describe AWC would be worthwhile. But the quantitative approach taken here, combined with mutant analysis, might provide a route to a more unified way of thinking about olfactory sensory neurons in the worm. And I would be interested in an alternative to (or reconciliation with) the adaptive threshold model presented by Levy and Bargmann.

Indeed, TAX-6 has been shown to play a central role in controlling adaptation in a number of sensory neurons and modalities of *C. elegans* (e.g, the thermosensory AFD neurons, the polymodal ASH neurons, and in the olfactory AWC neurons, Kuhara et al Neuron 2002). For example, *tax-6* mutant animals are hyper-adaptive to odorant pre-exposure and AWC-specific rescue restores this phenotype. This indeed positions TAX-6 as a key protein in mediating sensory adaptation. Of note, in the above study, the scored output was behavior while we focus intracellular calcium fluxes. The impiared calcium adaptation may explain the observed enhanced behavioral adaptation.

We have now added this information to the discussion section, page 23, last paragraph:
"... previous report showing that TAX-6 can cell-autonomously control adaptation in a number of sensory neurons and modalities (e.g, the thermosensory AFD neurons, the polymodal ASH neurons, and in the olfactory AWC neurons(Kuhara et al, 2002)). For example, tax-6 mutants are hyper-adaptive to odorant pre-exposure and AWC-specific rescue restores this phenotype. Thus, our observations that tax-6 mutants show long-lasting calcium fluxes that fail to resume basal pre-stimulus levels may explain the hyper-adaptation phenotypes scored via behavioral outputs (Kuhara et al, 2002).."

In addition , we have now contrasted our model with that of the adaptive threshold model proposed by Levy and Bargmann (Neuron 2020). We find that indeed with few simplifications, our model can be viewed as analogous to that model when describing the initiation timing of a neural response (or a pulse). A detailed mathematical derivation of this comparison can be found in the Appendix Note S1 (part 5).

And we also explain this in the text, page 25, second paragraph:
"Another model for neural activation was proposed by Levy and Bargmann, known as the adaptive-threshold mechanism (Levy & Bargmann, 2020). According to this mechanism, the threshold for initiating a neural response in the AWC sensory neurons is not fixed, but rather a dynamic variable that changes depending on the history of the perceived stimulus. Our experimental design and modeling results, which considered smooth continuous gradient stimuli, carry such stimulus history, and with few assumptions, our model is mathematically analogous to the adaptive-threshold model (A detailed mathematical derivation is found in ***Appendix Note S1 part 5)****."*

In summary, I think the conceptual discoveries of the paper can be more clearly described and honestly presented. I think their discovery about TAX-6 is very important and deserves publication. But it could be strengthened by a broader understanding of other candidate genes in the signaling pathway of AWA, or a broader understanding of TAX-6 in other olfactory neurons. The authors are onto something. This is important work, but could be greatly improved without too much more work.

We believe our new additional data further supports adn strengthens our conclusions. Particularly the analyses of additional mutants and the generation of the cell-specific TAX-6 rescue. We have also better explained our results in light of all the comments raised above.

References

1. Rahi, S. J. et al. Oscillatory stimuli differentiate adapting circuit topologies. Nature Methods 14, 1010-1016. ISSN: 15487105 (2017).

2. Kahn-Kirby, A. H. et al. Specific polyunsaturated fatty acids drive TRPV-dependent sensory signaling in vivo. Cell 119, 889-900. ISSN: 00928674 (2004).

3. Liu, Q., Kidd, P. B., Dobosiewicz, M. & Bargmann, C. I. C. elegans AWA Olfactory Neurons Fire Calcium-Mediated All-or-None Action Potentials. Cell 175, 57-70.e17. ISSN: 10974172 (Sept. 2018).

4. Fukuto, H. S. et al. G Protein-Coupled Receptor Kinase Function Is Essential for Chemosensation in C. elegans. eng. Neuron 42, 581-593. ISSN: 0896-6273 (Print) (May 2004).

5. Merritt, D. M. et al. Title: Arrestin-mediated Desensitization Enables Olfactory Discrimination in C. elegans. bioRxiv, 2021.05.02.439367 (2021).

6. Kuhara, A., Inada, H., Katsura, I. & Mori, I. Negative regulation and gain control of sensory neurons by the C. elegans calcineurin TAX-6. Neuron 33, 751-763. ISSN: 08966273 (2002).

7. Hajdu-Cronin, Y. M., Chen, W. J., Patikoglou, G., Koelle, M. R. & Sternberg, P. W. Antagonism between G(o)α and G(q)α in Caenorhabditis elegans: The RGS protein EAT-16 is necessary for G(o)α signaling and regulates G(q)α activity. Genes and Development 13, 1780-1793. ISSN: 08909369 (1999).

8. Larsch, J. et al. A Circuit for Gradient Climbing in C. elegans Chemotaxis. Cell Reports 12, 1748-1760. ISSN: 22111247 (2015).

9. Tu, Y., Shimizu, T. S. & Berg, H. C. Modeling the chemotactic response of Escherichia coli to time-varying stimuli. Proceedings of the National Academy of Sciences of the United States of America 105, 14855-14860 (Sept. 2008).

10. Barkai, N. & Leibler, S. Robustness in simple biochemical networks. Nature 387, 913-917. ISSN : 00280836 (June 1997).

11. Yi, T. M., Huang, Y., Simon, M. I. & Doyle, J. Robust perfect adaptation in bacterial chemotaxis through integral feedback control. Proceedings of the National Academy of Sciences of the United States of America 97, 4649-4653. ISSN: 00278424 (2000).

12. Itskovits, E., Ruach, R. & Zaslaver, A. Concerted pulsatile and graded neural dynamics enables efficient chemotaxis in C. elegans. 9. ISSN: 20411723. doi:10.1038/s41467- 01805151-2 (2018).

13. Roayaie, K., Crump, J. G., Sagasti, A. & Bargmann, C. I. The Gα protein ODR-3 mediates olfactory and nociceptive function and controls cilium morphogenesis in C. elegans olfactory neurons. Neuron 20, 55-67. ISSN: 08966273 (1998).
14. Levy, S. & Bargmann, C. I. An Adaptive-Threshold Mechanism for Odor Sensation and Animal Navigation. Neuron 105, 534-548.e13. ISSN: 10974199 (2020).

Reviewer #3:
In this manuscript, Ruach and colleagues examine the molecular mechanisms underlying the coding of odor in the AWA chemosensory neuron of C. elegans. The work elegantly combines the experimental inspection of the AWA neuron in wild-type and mutant backgrounds with modeling to reveal the critical role of feedback inhibition on the GPCR signaling involved in the detection of odor stimluli. The authors developed a parsimonious ODE model for the GPCR signaling cascade coupled to the TRPV and the voltage-gated ion channels responsible for the depolarization dynamics of the neuron, including its adaptive and pulsatile properties. I find the parsimonious model impressive: instead of trying to model the GPCR signaling pathway in detail, the authors opted for a coarse-grained representation that aims to abstract essential features of complex regulatory processes. As a result, modeling genuinely assists the functional dissection of the signal transduction cascade without getting bogged down with the biophysical description of mechanisms that remain poorly understood.

The parsimonious model captures essential features of the AWA response: calcium-dependent and calcium-independent inhibition, exact adaptation. One of the main findings of the study is that the mechanism by which the AWA neuron adapts to odorant signals relies on a negative feedback loop. The authors report that Calcineurin tax-6 creates an inhibitory feedback on the GPCR pathway. The parsimonious model helps establish that the inhibitory feedback mediated by tax-6 is calcium-independent. Although the exact mode of action of Calcineurin tax-6 remains to be determined in future work, the characterization of its loss of function on the AWA response represents an important step toward understanding how a single neuron perform complex computations to efficiently encode complex stimulus patterns necessary to efficiently navigate chemosensory gradients.

The manuscript is clearly written. I enjoyed all the references made to bacterial chemotaxis, which represents a source of inspiration to conceptualize sensory coding and formulate mechanistic hypotheses. Key results described in the main figures are supported by extra material in the supplementary material. I nonetheless believe the authors should strengthen the evidence supporting some of their claims. Below, I listed a series of concerns and suggestions for the authors to improve the manuscript. None of these concerns are major, though some suggestions might require additional experimental work.

(1) The work argues about the ethological relevance of pulsatile coding in the AWA chemosensory neuron - this idea builds on a previous publication from the same lab (Itskovits et 2018). In particular, the authors show that the AWA neuron of tax-6 mutants are unable to generate pulsatile

activity and to achieve exact adaptation in response to natural stimuli. If the calcium pulses control the reorientation dynamics of the worm, the implications of the study is that tax-6 mutants should have a severe deficit in chemotaxis. Would the authors predict that reorientation maneuvers would be inhibited in the tax-6 mutants? It would be important to validate this prediction experimentally.

Indeed, *tax-6* mutants show severe impaired chemotaxis. This was demonstrated before in several experiments (for example, in Kuhara et al . Neuron 2002, Figure 5). These experiments measured chemotaxis index which scored the number of animals reaching the target without tracking the fine locomotion trajectories. We therefore repeated these experiments, this time imaging the worms throughout the experiment and extracting trajectories. Indeed, *tax-6* mutants were highly defective in chemotaxis. In fact, they hardly made direct trajectories, but rather moved in circles in all directions (hardly even leaving the starting point region).
A possible explanation for this is that the lack of pulsatile activity in *tax-6* mutants prevents them from moving towards the target. We also showed in our previous MS (Itskovits et al 2018) that after AWA pulses, and before reaching exact adaptation, AWC activity induces a turn (was also shown by Larsch et al , PNAS 2013). Following this turn, the inability of AWA neurons to produce new pulses, prevents the ability of extracting odorant gradient information, possibly explaining why these animals move in circles with no apparent target.

Moreover, We speculate that since TAX-6 is ubiquitously expressed, it affects development and proper functions of many other neurons involved in coordinated locomotion, and hence, it is difficult to relate activity of a single neuron to complex locomotion outputs.

We have added exemplary movies of wt and *tax-6* mutants (github) to show how aberrant their locomotion is.

From the seminal study of Pierce-Shimomura and Lockery 1999, the typical duration of a "run" is on the order of ~20s. By contrast, the pulses of calcium in the AWA neuron tend to take place on a timescale of ~100s. What explains this apparent mismatch between the neuronal activity and the reorientation behavior?

We have now analyzed the duration of the pulses for wt animals in response to sigmoidal gradients (from Appendix Figure S3). Our analysis indicates that the average is 35 s +/- 10 s. Figure 3C shows a zoomed-in example of an activity trace where it is clear that the duration is ~ 30-40 s. Likewise, Fig 4B-C shows a typical duration of ~20 s following an on step.
Furthermore in Itskovits et al (2018), we demonstrated that the decay constant ranges between ~7-10 s. Thus, the decreasing phase of the pulse is ~20-30 s (activity increase time is almost instantaneous, lasting only a few seconds). Together, all these results indicate that the duration is ~30 s. It is also important to note that these measurements were extracted from animals restrained in a microfluidic device. Freely-behaving animals may have additional sensorimotor feedback processes that may affect the duration of the pulse, and hence the possible slight difference between the measurements.

(2) The work focuses on two features of the AWA response: (1) exact adaptation in response to a single step of the stimulus; (2) adaptation of pulsatile responses to the magnitude of the gradient's first derivative. Reproducing these properties was used to define the parameters of the parsimonious ODE model. It would be helpful if the authors could clarify in the main text how the parameter optimization was achieved, before introducing the perturbative analysis of the model's parameters.

We now better explain that we did not optimize to extract parameters. Some of the parameters were experimentally estimated (e.g. Calcium decay constant [tauC], recovery time from inhibition [tauI]). Appendix Table S1 details which parameters were extracted based on the experimental data. The other parameters were inferred manually to qualitatively describe the general features of the activity traces. It is plausible that the parameter's space includes additional ranges of possible values.

We now explain this in the Methods, page 29:
*"**Model construction and parameter evaluation.** We used MATLAB (Mathworks © Inc.) for implementing numerical simulations of the dynamical system (**Equations 1-4 and Appendix Note S1 part 1**). As we did not fit the model to the data, but rather aimed to construct a 'toy' model that captures the fine dynamic features observed experimentally, some of the parameters were manually varied by orders of magnitude (see full description in **Appendix Table S1**). Parameters, for which we had experimental evidence for, were fixed based on available data. **Appendix Table S1** provides the description of the parameters and how they were evaluated. "*

Strikingly, the exact adaptation of the AWA response is only evaluated based on odor pulses (diacethyl) added to an odor background of 1.15mM. The adaptation to the first derivative is assessed on a single sigmoid function. Given that the study relies on the goodness of fit of the model with experimental observations, the authors should extend their validation to odor steps added to different background concentrations (along the lines of what is done in Supp. Fig. 1d). They should also use patterns of odor stimulation where the first derivative varies. This analysis would validate the claim that "the amplitude and the frequency of the pulses correlate with the gradient's first derivative and adapt to it" (page 7).

The suggested experiments were actually performed in previous studies so that we did not need to repeat them. In Itskovits et al (2018), we provided a thorough analysis for different sigmoidal gradients (Figure 3 in that paper) showing that the pulsatile response adapts to the 1st derivative of the gradient, regardless of the absolute concentration. Furthermore, when exposing the worms to increasing exponential gradients, we showed that the higher the magnitude of the gradient's first derivative, the higher is the amplitude and the frequency of the pulses (supplementary figure 7 in Itskovits et al).

In addition, Larsch et al (Cell reports 2015, figure 6) analyzed AWA responses to successive fold-change increases in odor concentrations. It was found that small fold-change increases did not elicit prominents calcium transients regardless of the absolute odor concentration. Higher fold-change increases elicited calcium transients with roughly similar amplitudes. Both of these qualitative observations are captured by our model.

We now details all this in the first para. of the results, page 5:

*"Studies in C. elegans worms revealed that a single sensory neuron type, AWA, cell-autonomously implements key features required for efficient chemotaxis: (1) It codes the ligand concentration in a logarithmic-like scale, so that neural responses remain similar across orders of magnitude of ligand concentration (Larsch et al, 2015; Itskovits et al, 2018). (2) It responds with a single pulse to a step function and with multiple pulses during a continuous gradual increase in the stimulus concentration (**Figure 2A** and (Itskovits et al, 2018; Larsch et al, 2015, 2013)). (3) It shows an exact adaptation following a step function of the stimulus (**Figure 2A** and (Larsch et al, 2015; Itskovits et al, 2018)). (4) The mean frequency and the amplitude of the pulses, induced by smooth increasing stimuli, correlates with the first derivative of the gradient and also adapts to it (**Figure 2B** and (Itskovits et al, 2018)). Thus, the steeper the change in the gradient (higher first derivative), the higher are the frequency and the amplitude of the neural activity. On longer time scales, this activity adapts to the magnitude of the stimulus first derivative, such that the frequency and the amplitude of the pulses decrease if not facing increasing first derivative changes in the stimulus."*

(3) The authors discuss the "periodic skipping" of calcium pulses in the AWA neuron upon odor pulses at high frequencies (page 8). The skipping is not reproduced by the simulations of Supp. Fig. 1a-c. While the calcium pulses become attenuated, they do not disappear. Could the authors explain what might be missing in the model to fully account for this behavioral observation?

We now better explain how our model actually accounts for the periodic skipping. Appendix Figure S1C shows simulations in which nine consecutive stimulus steps result in responses to every other step (5 responses in total). We observe this skipping phenomenon for repetitive steps in which the off-step is sufficiently short and the on-step is sufficiently long to induce inhibition. In such a scenario, a strong inhibition following an on-step is maintained during the short off step interval, thus preventing a pulsatile response following a subsequent on-step. In Appendix Figure S1A-B, the off-steps are sufficiently long to prevent pulsatile skipping. These simulations fit the findings by Larsch et al (*Cell reports 2015, figure 2*) in which no periodic skipping is observed for 30-sec off and 30-sec on steps.
These explanations are now provided in the legend of Appendix Figure S1.

Likewise, the relaxation dynamics of the tax-6 mutant predicted by the model is much shorter than that observed experimentally in Fig 6a. What regulatory processe(s) might be missing to capture the relaxation kinetics more accurately in the tax-6 mutants?

Indeed. We find two reasons for this difference. The immediate abrupt decrease in the simulations is due to the fast inactivation of the VGCCs as modeled by Liu et al, 2018. We integrated the equations used in that manuscript to our model without changing parameters.

Following this fast decrease, in the simulations, calcium levels quickly reach an intermediate steady state, while in the experiments, their levels continue to gradually decrease. This is due to the interplay between the TAX-6-mediated calcium-dependant and the calcium-independent inhibitory processes. We simulated TRPV and VGCCs activity (S) as a switch, such that they transition

between on and off states depending whether the levels of active receptors cross a threshold (eq. 2). In the absence of TAX-6-mediated calcium-dependant inhibition, S remains on. During this time, calcium-independent processes gradually decrease levels of active receptors that lead to gradual decrease in S and calcium levels (as observed in the experiment). However, due to the switch-like nature of S in the model, this is not translated to gradual decrease in S (which stays on) and consequently, in calcium levels.

(4) Could you define what you mean by "adaptation" and "habituation"? Habituation is generally defined as a decrease in behavioral response that is independent of sensory adaptation. I am not convinced that this definition applies to the long-term loss of sensitivity reported following a prolonged odor pulse or a series of odor pulses. This phenomenon appears to fit within the realm of sensory adaptation, unless you expect it to depend on higher order processes that involve other neurons. You might need to revise your terminology accordingly.

When using the term ‘habituation’ we followed Larsch et al (*Cell reports 2015)* who defined this term as the decrease in the pulse amplitude following repeated steps of the stimulus.
We now explicitly define what we mean by habituation, on page 12:
*“*To analyze possible habituation, a reduction in the neural response between consecutive on-steps (Larsch et al, 2015), we inflicted a second short on-step following two minutes off-step.”

When using the term ‘adaptation’, we refer to two different features: (1) (exact) adaptation, in which calcium levels return to the pre-stimulus basal levels, and (2) adaptation of the pulsatile response (frequency and amplitude of the pulses) to the first derivative of the gradient. We now made an effort to explicitly explain these terms in the MS as we discuss them.

<u>Typos</u>:

- The reference to "Fechner" finishes by N.D. and not a date.

Fixed

- Some of the references that include multiple authors are cited as single authored.

We used the automated reference generator according to the EMBO press format, so such bugs are now fixed.

- Page 3, 3rd paragraph: should "none-active" receptors read "inactive"?

Fixed

Manuscript Number: MSB-2021-10514R
Title: A negative feedback loop in the GPCR pathway underlies efficient coding of external stimuli

Thank you for sending us your revised manuscript. We have now heard back from the three reviewers who were asked to evaluate your revised study. As you will see below, the reviewers think that the study has improved as a result of the performed revisions. However, reviewers #1 and #3 raise a few remaining concerns, which we would ask you to address in a final round of minor revisions.

We would also ask you to address some remaining editorial issues listed below.

Reviewer #1:

The authors have adequately addressed most of the previous reviewer comments. I think the manuscript is acceptable for publication once the following minor problems are solved.

1) Which promoter was used for the AWA-specific expression? I was not able to find the info in the Method section.
2) For the reference by Desrochers et al., year info is missing.
3) The paperpile link in the Reference section didn't work at all.

Reviewer #2:

The authors have done a very nice job addressing all of my comments. The paper is put into a better context, and the additional experiments strengthen their conclusions. This is a much improved study, and I have no further comments.

Reviewer #3:

I thank the authors for the discussion and detailed explanations they provided in their rebuttal letter. Their revised manuscript is mostly addressing the questions and concerns of my first report. I nonetheless remain unconvinced about the following points:

1. The authors explained in their rebuttal letter that they repeated behavioral experiments with the tax-6 mutant. They report that "tax-6 mutants were highly defective in chemotaxis. In fact, they hardly made direct trajectories, but rather moved in circles in all directions (hardly even leaving the starting point region)." I searched for these results in the revised manuscript, but I couldn't find them. The data are not in the rebuttal letter either. Where were these results presented? As argued by the authors, repeating the analysis of the tax-6 mutants is valuable because complete trajectories could be tracked and quantified. This analysis should be added to the manuscript.

2. The authors clarified that the parsimonious mathematical model for the response of AWA is not based on any parameter optimization. Instead, the aim was to "construct a 'toy' model that captures the fine dynamic features observed experimentally." And the authors admit that "it is plausible that the parameter's space includes additional ranges of possible values." In my view, the idea that the model is just 'toy' that can be tweaked by hand is inconsistent with the importance that this model holds in the manuscript. While a more rigorous search of the parameter space of the model might not change the overall results of the manuscript, it would have been a good practice in line with the field's standards. This would make the model potentially more useful to other researchers in the future.

3. In the discussion, the authors write "Studies in C. elegans worms revealed that a single sensory neuron type, AWA, cell-autonomously implements key features required for efficient chemotaxis: (1) It codes the ligand concentration in a logarithmic-like scale, so that neural responses remain similar across orders of magnitude of ligand concentration (Larsch et al, 2015; Itskovits et al, 2018)." The statement about the logarithmic sensitivity suggests that this result has been solidly demonstrated experimentally. By contrast, the authors stated in their response to reviewer #1 that logarithmic dependence on ligand concentration is a "conjecture". The fact that the logarithmic sensitivity would be a conjecture is not evident from the phrasing used in the manuscript. This point should be clarified.

Below are our detailed responses to the reviewer's comments:

Reviewer #1:

The authors have adequately addressed most of the previous reviewer comments. I think the manuscript is acceptable for publication once the following minor problems are solved.

1) Which promoter was used for the AWA-specific expression? I was not able to find the info in the Method section.

In the Methods section, we detail how we generated the strain and that we used the gpa-6 promoter:

“”ZAS489 [tax-6(p675); lite-1(ce314); pha-1(e2123); gpa-6::GCaMP3; pha-1::PHA-1; azrEx[gpa-6::TAX-6-DsRed; unc-122::GFP]] was generated as following: The TAX-6 coding sequence was amplified from N2 cDNA library using the following primers: forward - ATGGCCTCGACATCGGC; reverse - TTAGCTATTTGATGGACCATTTTG. It was then cloned into the pPD95.77 plasmid upstream and in frame to DsRed. Next, we fused the pcr segment of TAX-6-DsRed from the pPD95.77 plasmid to the gpa-6 promoter amplified from a genomic DNA (Zaslaver et al, 2015; Bokman et al, 2022). Finally, we injected the linear fragment to ZAS282 together with unc122::GFP. The resulting transgenic animal showed AWA-exclusive expression in the head, and in another cell in the tail.”

2) For the reference by Desrochers et al., year info is missing.

The year 2018 was added.

3) The paperpile link in the Reference section didn't work at all.

The links should be functional once converted and edited according to the Journal style.

Reviewer #2:

The authors have done a very nice job addressing all of my comments. The paper is put into a better context, and the additional experiments strengthen their conclusions. This is a much improved study, and I have no further comments.

Reviewer #3:

I thank the authors for the discussion and detailed explanations they provided in their rebuttal letter. Their revised manuscript is mostly addressing the questions and concerns of my first report. I nonetheless remain unconvinced about the following points:

1. The authors explained in their rebuttal letter that they repeated behavioral experiments with the tax-6 mutant. They report that "tax-6 mutants were highly defective in chemotaxis. In fact,

they hardly made direct trajectories, but rather moved in circles in all directions (hardly even leaving the starting point region)." I searched for these results in the revised manuscript, but I couldn't find them. The data are not in the rebuttal letter either. Where were these results presented? As argued by the authors, repeating the analysis of the tax-6 mutants is valuable because complete trajectories could be tracked and quantified. This analysis should be added to the manuscript.

We now provide the chemotaxis movies for wt and *tax-6* mutants (Movie EV2 and Movie EV3, respectively). We have also analyzed animal's trajectories and provide the full analyses in a new Appendix Figure S7 (also provided below). These results show that indeed *tax-6* mutants are defective in chemotaxis.

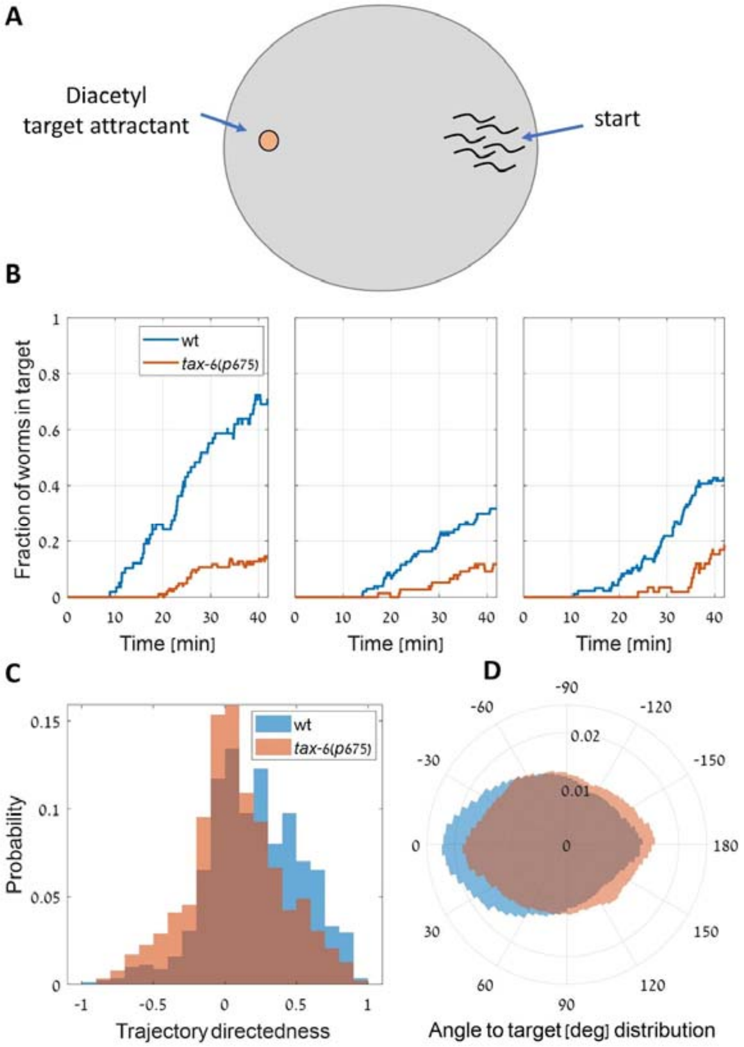

2. The authors clarified that the parsimonious mathematical model for the response of AWA is not based on any parameter optimization. Instead, the aim was to "construct a 'toy' model that captures the fine dynamic features observed experimentally." And the authors admit that "it is plausible that the parameter's space includes additional ranges of possible values." In my view, the idea that the model is just 'toy' that can be tweaked by hand is inconsistent with the importance that this model holds in the manuscript. While a more rigorous search of the parameter space of the model might not change the overall results of the manuscript, it would have been a good practice in line with the field's standards. This would make the model potentially more useful to other researchers in the future.

Indeed, it is usually a good practice to fit a model by rigorously scanning the parameter's space in search of a global minimum of a loss function between the experimental data and a model. However, we believe that in this study, such an approach will not be very helpful in gaining a better understanding of the system. This is due to the large variability in the pulse's characteristics (frequency, amplitude etc.) that is observed across the assayed animals. This significant variability requires using a different set of parameters for each animal to reliably describe its idiosyncratic pulsatile dynamics. In addition, our model is required to depict several response dynamics simultaneously (a sigmoidal function, a single step function, various multiple steps protocols and exponential/linear gradients). However, these experiments were not performed on the same worm, so there is no single dataset that we can use to fit all these different responses. Instead, we analyzed the structure of the parameter's space to show that it can support various features of the pulses while implementing the observed features (e.g. exact adaptation, adaptation to the first derivative etc.)

3. In the discussion, the authors write "Studies in C. elegans worms revealed that a single sensory neuron type, AWA, cell-autonomously implements key features required for efficient chemotaxis: (1) It codes the ligand concentration in a logarithmic-like scale, so that neural responses remain similar across orders of magnitude of ligand concentration (Larsch et al, 2015; Itskovits et al, 2018)." The statement about the logarithmic sensitivity suggests that this result has been solidly demonstrated experimentally. By contrast, the authors stated in their response to reviewer #1 that logarithmic dependence on ligand concentration is a "conjecture". The fact that the logarithmic sensitivity would be a conjecture is not evident from the phrasing used in the manuscript. This point should be clarified.

The experimental observations convincingly demonstrate that AWA response amplitudes remain within the same order of magnitude in response to several orders of magnitude change in the stimulus (Larsch et al, 2015; Itskovits et al, 2018). We refer to this phenomenon as "logarithmic-like" scaling (coding), since the underlying mechanism involves a logarithmic-like function that rescales orders of magnitude of the input to a much narrower range of the output. However, the exact functional transformation of the AWA input-output was not demonstrated and could in fact involve a more complicated form of such a logarithmic relationship. This is the reason why we referred to the logarithmic input-out relationship, or specifically the dependence of receptor activity to ligand concentration, as a conjecture.

We have now modified the manuscript to state that the coding is 'logarithmic-like'.
For example, in the beginning of the Results section:
"Studies in *C. elegans* worms revealed that a single sensory neuron type, AWA, cell-autonomously implements key features required for efficient chemotaxis: (1) It codes the ligand concentration in a logarithmic-like scale, so that neural responses remain similar across orders of magnitude of ligand concentration (Larsch et al, 2015; Itskovits et al, 2018)."

And also in the 2nd paragraph of the discussion:
"Notably, the GPCR negative feedback supports several important computational features, including: (1) Coding ligand concentration on a logarithmic-like scale, thus enabling adjusted responses across several orders of magnitude of the stimulus."

RE: MSB-2021-10514RR, A negative feedback loop in the GPCR pathway underlies efficient coding of external stimuli

Thank you again for sending us your revised manuscript. We are now satisfied with the modifications made and I am pleased to inform you that your paper has been accepted for publication.

EMBO Press Author Checklist

Corresponding Author Name: Alon Zaslaver	
Journal Submitted to: MSB	
Manuscript Number: 2021-10514	

USEFUL LINKS FOR COMPLETING THIS FORM
The EMBO Journal - Author Guidelines
EMBO Reports - Author Guidelines
Molecular Systems Biology - Author Guidelines
EMBO Molecular Medicine - Author Guidelines

Reporting Checklist for Life Science Articles (updated January
This checklist is adapted from Materials Design Analysis Reporting (MDAR) Checklist for Authors. MDAR establishes a minimum set of requirements in transparent reporting in the life sciences (see Statement of Task: 10.31222/osf.io/9sm4x). Please follow the journal's guidelines in preparing your
Please note that a copy of this checklist will be published alongside your article.

Abridged guidelines for figures
1. Data
The data shown in figures should satisfy the following conditions:
- the data were obtained and processed according to the field's best practice and are presented to reflect the results of the experiments in an accurate and unbiased manner.
- ideally, figure panels should include only measurements that are directly comparable to each other and obtained with the same assay.
- plots include clearly labeled error bars for independent experiments and sample sizes. Unless justified, error bars should not be shown for technical
- if n<5, the individual data points from each experiment should be plotted. Any statistical test employed should be justified.
- Source Data should be included to report the data underlying figures according to the guidelines set out in the authorship guidelines on Data

2. Captions
Each figure caption should contain the following information, for each panel where they are relevant:
- a specification of the experimental system investigated (eg cell line, species name).
- the assay(s) and method(s) used to carry out the reported observations and measurements.
- an explicit mention of the biological and chemical entity(ies) that are being measured.
- an explicit mention of the biological and chemical entity(ies) that are altered/varied/perturbed in a controlled manner.
- the exact sample size (n) for each experimental group/condition, given as a number, not a range;
- a description of the sample collection allowing the reader to understand whether the samples represent technical or biological replicates (including how many animals, litters, cultures, etc.).
- a statement of how many times the experiment shown was independently replicated in the laboratory.
- definitions of statistical methods and measures:
  - common tests, such as t-test (please specify whether paired vs. unpaired), simple χ2 tests, Wilcoxon and Mann-Whitney tests, can be unambiguously identified by name only, but more complex techniques should be described in the methods section;
  - are tests one-sided or two-sided?
  - are there adjustments for multiple comparisons?
  - exact statistical test results, e.g., P values = x but not P values < x;
  - definition of 'center values' as median or average;
  - definition of error bars as s.d. or s.e.m.

Please complete ALL of the questions below.
Select "Not Applicable" only when the requested information is not relevant for your study.

Materials

Newly Created Materials	Information included in the manuscript?	In which section is the information available? (Reagents and Tools Table, Materials and Methods, Figures, Data Availability Section)
New materials and reagents need to be available; do any restrictions apply?	Not Applicable	

Antibodies	Information included in the manuscript?	In which section is the information available? (Reagents and Tools Table, Materials and Methods, Figures, Data Availability Section)
For **antibodies** provide the following information:<br>- Commercial antibodies: RRID (if possible) or supplier name, catalogue number and or/clone number<br>- Non-commercial: RRID or citation	Not Applicable	

DNA and RNA sequences	Information included in the manuscript?	In which section is the information available? (Reagents and Tools Table, Materials and Methods, Figures, Data Availability Section)
**Short novel DNA or RNA including primers, probes**: provide the sequences.	Yes	Materials and Methods

Cell materials	Information included in the manuscript?	In which section is the information available? (Reagents and Tools Table, Materials and Methods, Figures, Data Availability Section)
**Cell lines:** Provide species information, strain. Provide accession number in repository **OR** supplier name, catalog number, clone number, and/**OR** RRID.	Not Applicable	
**Primary cultures:** Provide species, strain, sex of origin, genetic modification status.	Not Applicable	
Report if the cell lines were recently **authenticated** (e.g., by STR profiling) and tested for mycoplasma contamination.	Not Applicable	

Experimental animals	Information included in the manuscript?	In which section is the information available? (Reagents and Tools Table, Materials and Methods, Figures, Data Availability Section)
**Laboratory animals or Model organisms:** Provide species, strain, sex, age, genetic modification status. Provide accession number in repository **OR** supplier name, catalog number, clone number, **OR** RRID.	Yes	Materials and Methods
**Animal observed in or captured from the field:** Provide species, sex, and age where possible**.**	Not Applicable	
Please detail **housing and husbandry conditions**.	Not Applicable	

Plants and microbes	Information included in the manuscript?	In which section is the information available? (Reagents and Tools Table, Materials and Methods, Figures, Data Availability Section)
**Plants:** provide species and strain, ecotype and cultivar where relevant, unique accession number if available, and source (including location for collected wild specimens).	Not Applicable	
**Microbes:** provide species and strain, unique accession number if available, and source.	Not Applicable	

Human research participants	Information included in the manuscript?	In which section is the information available? (Reagents and Tools Table, Materials and Methods, Figures, Data Availability Section)
If collected and within the bounds of privacy constraints report on age, sex and gender or ethnicity for all study participants.	Not Applicable	

Core facilities	Information included in the manuscript?	In which section is the information available? (Reagents and Tools Table, Materials and Methods, Figures, Data Availability Section)
If your work benefited from core facilities, was their service mentioned in the acknowledgments section?	Not Applicable	

Design

Study protocol	Information included in the manuscript?	In which section is the information available? (Reagents and Tools Table, Materials and Methods, Figures, Data Availability Section)
If study protocol has been **pre-registered, provide DOI in the manuscript**. For clinical trials, provide the trial registration number **OR** cite DOI.	Not Applicable	
Report the **clinical trial registration number** (at ClinicalTrials.gov or equivalent), where applicable.	Not Applicable	

Laboratory protocol	Information included in the manuscript?	In which section is the information available? (Reagents and Tools Table, Materials and Methods, Figures, Data Availability Section)
Provide DOI OR other citation details if **external detailed step-by-step protocols** are available.	Not Applicable	

Experimental study design and statistics	Information included in the manuscript?	In which section is the information available? (Reagents and Tools Table, Materials and Methods, Figures, Data Availability Section)
Include a statement about **sample size** estimate even if no statistical methods were used.	Yes	In all figure legends
Were any steps taken to minimize the effects of subjective bias when allocating animals/samples to treatment (e.g. **randomization procedure**)? If yes, have they been described?	Not Applicable	
Include a statement about **blinding** even if no blinding was done.	Not Applicable	
Describe **inclusion/exclusion criteria** if samples or animals were excluded from the analysis. Were the criteria pre-established?<br><br>If sample or data points were omitted from analysis, report if this was due to attrition or intentional exclusion and provide justification.	Not Applicable	
For every figure, are **statistical tests** justified as appropriate? Do the data meet the assumptions of the tests (e.g., normal distribution)? Describe any methods used to assess it. Is there an estimate of variation within each group of data? Is the variance similar between the groups that are being statistically compared?	Yes	In all figures and in Methods section

Sample definition and in-laboratory replication	Information included in the manuscript?	In which section is the information available? (Reagents and Tools Table, Materials and Methods, Figures, Data Availability Section)
In the figure legends: state number of times the experiment was **replicated** in laboratory.	Yes	In figure legends
In the figure legends: define whether data describe **technical or biological replicates**.	Yes	In figure legends

Ethics

Ethics	Information included in the manuscript?	In which section is the information available? (Reagents and Tools Table, Materials and Methods, Figures, Data Availability Section)
Studies involving **human participants**: State details of **authority granting ethics approval** (IRB or equivalent committee(s), provide reference number for approval.	Not Applicable	
Studies involving **human participants**: Include a statement confirming that **informed consent** was obtained from all subjects and that the experiments conformed to the principles set out in the WMA Declaration of Helsinki and the Department of Health and Human Services Belmont Report.	Not Applicable	
Studies involving **human participants:** For publication of **patient photos**, include a statement confirming that consent to publish was obtained.	Not Applicable	
Studies involving experimental **animals**: State details of **authority granting ethics approva**l (IRB or equivalent committee(s), provide reference number for approval. Include a statement of compliance with ethical regulations.	Not Applicable	
Studies involving **specimen and field samples:** State if relevant **permits** obtained, provide details of authority approving study; if none were required, explain why.	Not Applicable	

Dual Use Research of Concern (DURC)	Information included in the manuscript?	In which section is the information available? (Reagents and Tools Table, Materials and Methods, Figures, Data Availability Section)
Could your study fall under dual use research restrictions? Please check biosecurity documents and list of **select agents and toxins** (CDC): https://www.selectagents.gov/sat/list.htm	Not Applicable	
If you used a select agent, is the security level of the lab appropriate and reported in the manuscript?	Not Applicable	
If a study is subject to dual use research of concern regulations, is the name of the **authority granting approval and reference number** for the regulatory approval provided in the manuscript?	Not Applicable	

Reporting

The MDAR framework recommends adoption of discipline-specific guidelines, established and endorsed through community initiatives. Journals have their own policy about requiring specific guidelines and recommendations to complement MDAR.

Adherence to community standards	Information included in the manuscript?	In which section is the information available? (Reagents and Tools Table, Materials and Methods, Figures, Data Availability Section)
State if relevant guidelines or checklists (e.g., **ICMJE, MIBBI, ARRIVE, PRISMA**) have been followed or provided.	Not Applicable	
For **tumor marker prognostic studies,** we recommend that you follow the **REMARK** reporting guidelines (see link list at top right). See author guidelines, under 'Reporting Guidelines'. Please confirm you have followed these guidelines.	Not Applicable	
For **phase II and III randomized controlled trials**, please refer to the **CONSORT** flow diagram (see link list at top right) and submit the CONSORT checklist (see link list at top right) with your submission. See author guidelines, under 'Reporting Guidelines'. Please confirm you have submitted this list.	Not Applicable	

Data Availability

Data availability	Information included in the manuscript?	In which section is the information available? (Reagents and Tools Table, Materials and Methods, Figures, Data Availability Section)
Have **primary datasets** been deposited according to the journal's guidelines (see 'Data Deposition' section) and the respective accession numbers provided in the Data Availability Section?	Yes	In github
Were **human clinical and genomic datasets** deposited in a public access-controlled repository in accordance to ethical obligations to the patients and to the applicable consent agreement?	Not Applicable	
Are **computational models** that are central and integral to a study available without restrictions in a machine-readable form? Were the relevant accession numbers or links provided?	Yes	in Github
If publicly available data were reused, provide the respective **data citations in the reference list**.	Not Applicable	
